# Transcriptome Profiling of the Pacific Oyster *Crassostrea gigas* Visceral Ganglia over a Reproduction Cycle Identifies Novel Regulatory Peptides

**DOI:** 10.3390/md19080452

**Published:** 2021-08-07

**Authors:** Emilie Réalis-Doyelle, Julie Schwartz, Cédric Cabau, Lorane Le Franc, Benoit Bernay, Guillaume Rivière, Christophe Klopp, Pascal Favrel

**Affiliations:** 1 UMR BOREA, UNICAEN, MNHN, CNRS-8067, IRD-207, Normandie Université, Sorbonne Université, Esplanade de la Paix, 14032 Caen, France; emilie.realis@inrae.fr (E.R.-D.); julie.schwartz@unicaen.fr (J.S.); lorane.lefranc@unicaen.fr (L.L.F.); guillaume.riviere@unicaen.fr (G.R.); 2 Plateforme Sigenae, BP 52627, UR875 Mathématiques et Informatique Appliquées de Toulouse, INRAE, 31326 Castanet-Tolosan, France; cedric.cabau@inrae.fr (C.C.); christophe.klopp@inrae.fr (C.K.); 3 PROTEOGEN Core Facility SF 4206 ICORE, UNICAEN, Esplanade de la Paix, 14032 Caen, France; benoit.bernay@unicaen.fr

**Keywords:** nervous system, transcriptome, neuropeptides, reproduction

## Abstract

The neuropeptides involved in the regulation of reproduction in the Pacific oyster (*Crassostrea gigas*) are quite diverse. To investigate this diversity, a transcriptomic survey of the visceral ganglia (VG) was carried out over an annual reproductive cycle. RNA-seq data from 26 samples corresponding to VG at different stages of reproduction were de novo assembled to generate a specific reference transcriptome of the oyster nervous system and used to identify differentially expressed transcripts. Transcriptome mining led to the identification of novel neuropeptide precursors (NPPs) related to the bilaterian Eclosion Hormone (EH), crustacean female sex hormone/Interleukin 17, Nesfatin, neuroparsin/IGFBP, prokineticins, and urotensin I; to the protostome GNQQN, pleurin, prohormones 3 and 4, prothoracotropic hormones (PTTH), and QSamide/PXXXamide; to the lophotrochozoan CCWamide, CLCCY, HFAamide, and LXRX; and to the mollusk-specific NPPs CCCGS, clionin, FYFY, GNamide, GRWRN, GSWN, GWE, IWMPxxGYxx, LXRYamide, RTLFamide, SLRFamide, and WGAGamide. Among the complete repertoire of NPPs, no sex-biased expression was observed. However, 25 NPPs displayed reproduction stage-specific expression, supporting their involvement in the control of gametogenesis or associated metabolisms.

## 1. Introduction

As sessile organisms living in estuaries and intertidal zones, Pacific oysters (*Crassostrea gigas*) are particularly resilient to highly stressful and widely changing environmental conditions [1,2,3]. They are exposed daily to large variations in food supply, oxygen, temperature, and salinity over the tidal cycle. They can also tolerate extended periods of emersion. To cope with these adverse conditions, oysters do not develop complex behavioral responses like many free-living animals do, but adapt their physiological processes and their metabolism. In animals, the central nervous system constitutes the main integrator of environmental and internal cues, but the way sensory signals or internal conditions are perceived and subsequently turned into suitable actions is poorly understood [4]. As neuron-secreted specific signaling peptides, neuropeptides (NPs) represent essential components of neural communication, and thus play a major role in these regulatory processes. They act as neurotransmitters, neuromodulators, neurohormones, and growth factors. As neuromodulators, they coordinate complex motor programs and modulate neuronal circuit functional outputs [4,5]. They can also be released into the circulatory system and serve as neurohormones to regulate the activity of distant organs and contribute to vital biological processes such as growth, reproduction and development, digestion, water and ionic balance, metabolism, energy homeostasis, and the immune response [6]. Therefore, extensive knowledge of the repertoire, spatiotemporal, and environment-induced expression patterns of NPs can significantly contribute to exploring animal physiological regulation and unravel the underlying mechanisms of the adaptive responses to a changing environment. Advances in the knowledge of NP diversity in Bilateria has tremendously increased with the development of genomic and transcriptomic resources, especially regarding non-chordate deuterostomes such as echinoderms [7,8,9] and non-conventional model species of protostomes [10,11,12,13,14]. In mollusks—a major representative phylum of Lophotrochozoa—the repertoire of NPs is now well characterized based on the interplay between genome/transcriptome mining approaches and mass spectrometry identification of mature NPs in the main class-level taxa, including Gastropoda [15,16,17,18], Cephalopoda [19], Bivalvia [20,21], as well as other taxa [22]. In *C. gigas*, a variety of neuropeptide precursors (NPPs)/peptide precursors (PPs) has been identified thanks to the mining of genomic and transcriptomic resources [20,23,24,25,26]. These works also led to the functional characterization of specific NP signaling systems involved in feeding [27,28], as well as in the regulation of water and ionic balance [29,30] and energy storage [31]. The adult nervous system of *C. gigas* consists of a pair of tiny cerebral ganglia connected to a pair of coalesced visceral ganglia by a cerebrovisceral connective. Barring a recent study dedicated to its development [32], the *C. gigas* nervous system has been poorly investigated. In this species, most transcriptomic analyses were dedicated to investigating stress responses [2,33], local adaptation [34], or sex determination and differentiation [35,36], Thus, none of the available *C. gigas* transcriptomic resources have been generated from the adult nervous system alone. As a consequence, NPP-encoding transcripts are probably diluted in the existing transcriptomes, and those with weak expression levels in nervous tissues probably went undetected. This is why we constructed RNA-seq libraries specific to *C. gigas* male and female visceral ganglia (VG) over a complete reproductive cycle. The purpose of this study was first to obtain a more comprehensive overview of the diversity of NPPs in this species. The second objective was to identify key neural regulators of the different reproductive stages and also potentially sex differentiation, as *C. gigas* is an irregular alternative hermaphrodite mollusk.

## 2. Results and Discussion

### 2.1. VG Transcriptome Sequencing and Assembly

The quality of the transcriptome was checked by verifying its compaction, read realignment rates, and protein content. The assembly produced 37,518 contigs (65,462,578 bases) with an N50 at 2543 and an N90 at 860. The mapping rates of each library ranged from 93.86% to 98.09% (mean 97.1% +/− 1.07%). The pairing rates ranged from 91.6% to 96.3% (mean 95.1% +/− 1.14%). The protein content was checked using BUSCO with the vertebrate reference protein set (vertebrata_odb9). Over 73% (1895/2586) of the proteins expected by BUSCO were found in unique or duplicated copies in the dataset. Considering that our transcriptome was built from a single tissue type, this result is quite satisfactory. The abundance of all the transcripts was normalized and calculated using the TPM method [37]. About half of the transcripts was considered as not expressed or expressed at very low levels (0 < TPM < 1), whereas a small proportion—less than 5%—was highly expressed (TPM > 60).

A total of 32,719 (87.21%) transcripts were associated with a BLAST hit, whereas 4799 (12.79%) were not annotated based on our BLAST search. The major part, corresponding to 2130 contigs (67.55%), matched with already recorded *C. gigas* sequences.

### 2.2. General Patterns of Transcript Expression in Oyster VG during Gametogenesis

**Figure 1:**
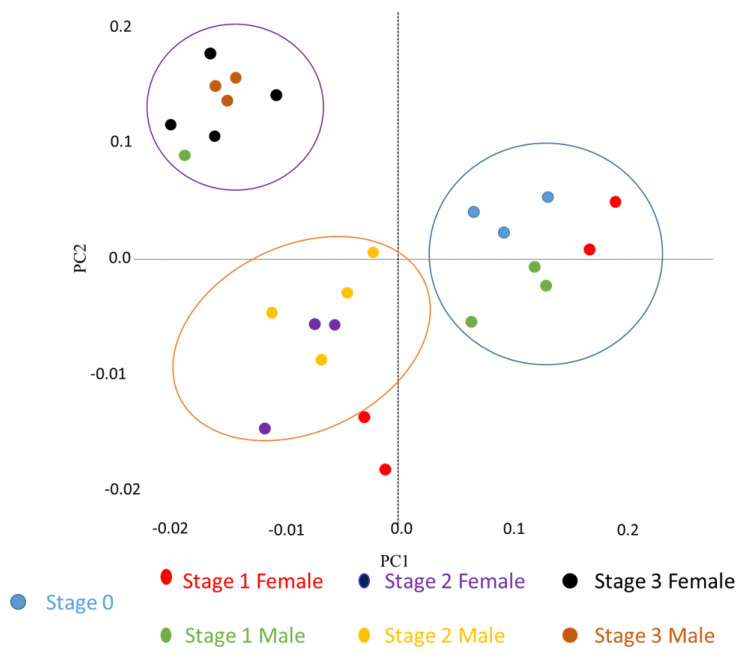
Principal component analysis (PCA) of expressed transcripts during gametogenesis. 2D score plot obtained by PCA of all 37,518 transcripts in the 26 pools of oyster visceral ganglia over a reproduction cycle.

The transcriptomes of the 26 male and female pools at different stages of reproduction were investigated using a statistical approach. A principal component analysis (PCA) was applied to the expression data of all 37,518 contigs to assess the internal consistency of the whole transcriptional dataset. The PCA was depicted with a plot in which similar transcriptional profiles clustered together (Figure 1). The first principal component (PC) explained 92.17% of the total variance. Other than two female stage-1 pools, a well-defined clustering was detected according to the gametogenesis stages. The overall segregation of the VG samples, according to the reproduction stage chronology along the PC1, strongly suggests the prevalence of a neural control of the oyster reproductive cycle. In contrast, no significant divergence in expression patterns between male and female VG was observed throughout the time course of gametogenesis. This implies that, in contrast to the gonads [36], the VG were sexually non-significantly distinct at the transcriptional level using this method. Since *C. gigas* is an alternative and irregular hermaphrodite, this result was somewhat surprising; we might have expected that transcriptome profile changes would reflect or would take part in the process of sex differentiation in this species. Interestingly, non-significant sex-biased expression differences in the nervous system were also observed in teleost fishes with various reproductive modes [38,39]. These observations may reflect that the nervous component acts as the triggering system of the reproduction timeline, whereas sex differentiation might rather be related to germinal stem cell identity factors which are still elusive to date in the oyster.

### 2.3. Identification of New NPP Transcripts

The NPPs previously identified in *C. gigas* were obtained by mining the genome sequences [3] or tissue transcriptomic resources [40,41]. Apart from the GigasDatabase [41], these resources did not include the nervous ganglia. In silico tBLASTn analysis of the assembled VG transcriptome generated in this study led to the identification of 96 transcripts encoding NPPs, including the 52 NPPs previously characterized in this species [20,24,25,26,30] and 44 newly identified NPPs (Supplementary Table S1). Developmental-stage-specific expression, tissue distribution, and expression level in the VG were determined over a whole reproductive cycle for these new NPPs (Supplementary Figure S1) by mining the GigaTON database [40] and the VG transcriptome database (this paper). In addition, mature NPs were investigated by MS (Supplementary Table S2). The new NPPs were grouped into four categories according to the sequences with which they were aligned (Supplementary Figure S1): (1) the oyster representatives of bilaterian NPPs, (2) oyster sequences belonging to protostome-specific neuropeptide families, (3) oyster homologs of annelid NPPs forming the lophotrochozoan families, and (4) oyster NPPs that may represent mollusk or even bivalve innovations.

### 2.4. Bilaterian Families

#### 2.4.1. Cragi-IL17/CFSH (Interleukin 17/Crustacean Female Sex Hormone)

**Figure 2:**
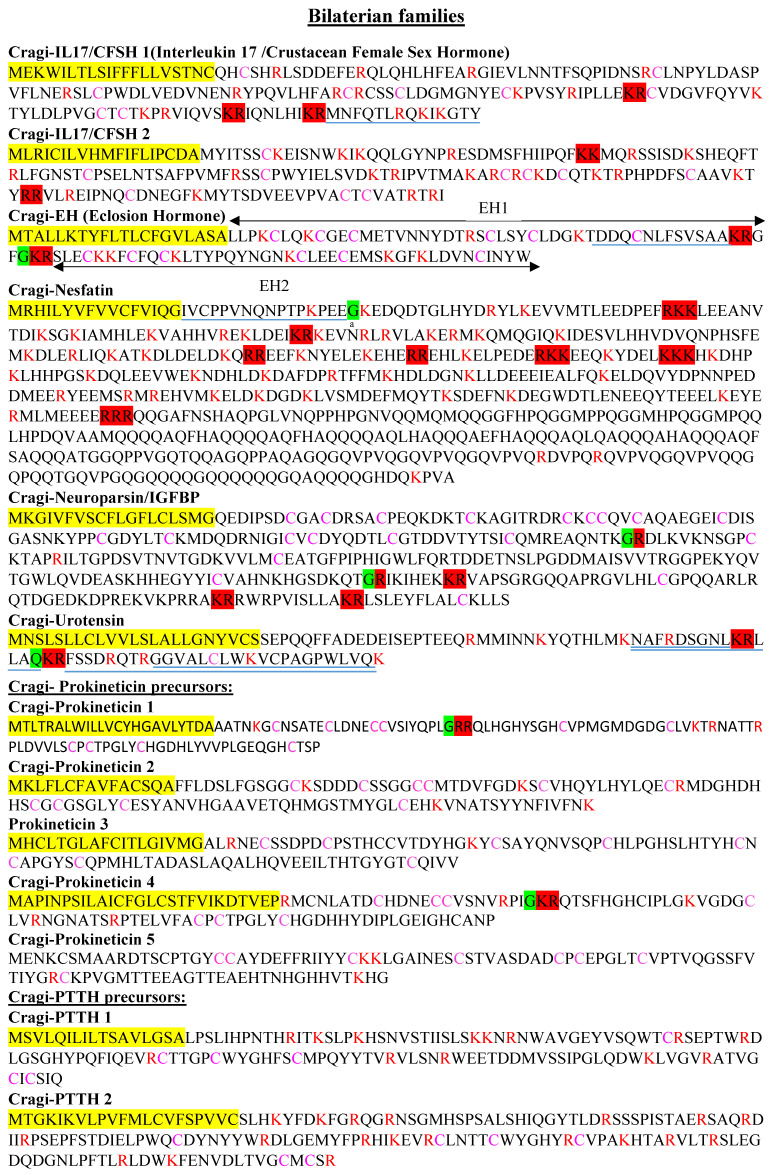

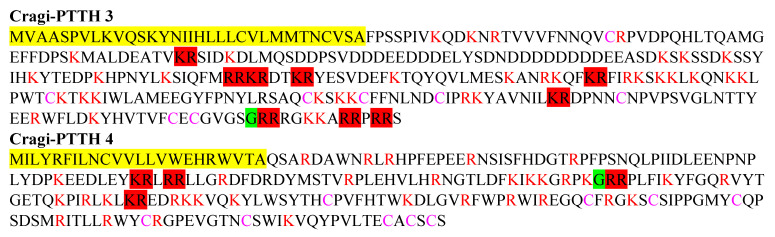
Novel bilaterian NPP types characterized in *C. gigas*. The predicted signal peptide is highlighted in yellow; the likely convertase processing sites are highlighted in red. The possible cleavages at a single arginine (R) or lysine (K) residues are written in red. The glycine residues likely to be converted into a C-terminal amide are highlighted in green. Cysteine residues are marked in purple. Mature NPs detected by MS are underlined by a blue line and the post-translational modifications: pyroglutamate (p) and C-terminal amide (a) are indicated.

A novel female sex-dominant neurohormone named crustacean female sex hormone (CFSH) was recently characterized from the eyestalk ganglia of blue crab (*Callinectes sapidus*). This new neurohormone positively regulates the development of adult-stage-dependent female reproductive structures [42]. In crayfish (*Procambarus clarkia*), the expression of the orthologous hormone was much higher in the ovary and was null in the testis [43]. The crustacean CFSH precursors typically comprise a signal peptide followed by a CFSH-related peptide separated from the CFSH mature peptide by a dibasic cleavage site [42]. The CFSH mature peptides display eight to six cysteine residues; the relative positions of the last six of these residues and of a number of other amino acid residues are shared [43]. A BLAST search of the oyster VG database using the *Carcinus maenas* CFSH sequence as a query yielded two precursors (Figure 2) that lacked the CFSH-related peptide; however, they harbored a mature CFSH peptide displaying the characteristic cysteine residues at the conserved positions. The two mature peptides predicted in oyster shared only 23% amino acid identity with each other, and had about 18% identity (38% similarity) with *C. maenas* CFSH. Interestingly, oyster CFSH peptides also present homology with vertebrate IL17 family members, suggesting that these proteins could have emerged from a common ancestral gene. Surprisingly, both oyster precursors were exclusively expressed—though at very low levels—in the VG. It remains speculative whether the presently characterized oyster proteins regulate reproduction-associated processes as CFSH does, or whether they mediate immunity functions in a similar manner to other homologous but distinct oyster IL-17 family members [44,45].

#### 2.4.2. Cragi-EH (Eclosion Hormone)

Eclosion hormones (EHs) are large peptides involved in the regulation of ecdysis behavior and other physiological changes associated with this complex process in insects [46,47] and crustaceans [48]. A complete primary structure of EH was first determined in the tobacco hornworm (*Manduca sexta*) [49]. Subsequently, EH representatives were discovered in arthropods. The finding of an EH precursor and EH guanylyl cyclase receptor orthologs in echinoderms [50] rationally dates back the origin of EH signaling in the ancestor of the Bilateria. EH signaling components have indeed been found in various lophotrochozoan phyla such as mollusks [22], annelids, and acoels [51], and also in cnidaria, but the receptor was not found in this latter phylum [51]. The oyster EH precursor oddly includes two EH ligands (Cragi-EH 1 and 2) separated by a dibasic cleavage site (Figure 2). The two ligands share only 24% sequence identity (49% similarity) but share the six cysteine residues involved in the formation of three disulfide bridges with the EH peptides of other species. As already stated for echinoderm EH [50], mature Cragi-EH peptides also exhibit some degree of sequence relatedness with arthropod ion transport peptides (ITPs), and with the crustacean hyperglycemic hormone (CHH), despite an incomplete alignment of the six cysteine residues. This suggests a possible common ancient evolutionary origin (Supplementary Figure S1).

Cragi-EH transcripts were mainly expressed in the female gonad, the mantle, and the digestive gland, and to a far lesser degree during late larval development. Cragi-EH expression was also detected in the VG, but no significant variation was observed throughout the reproductive cycle. MS analysis failed to identify these large peptides, except a peptide corresponding to the C-terminal region of Cragi-EH1—possibly a non-specific proteolytic product generated during the extraction step. In arthropods, EH is engaged in the complex actions and interactions among several peptide hormones, leading to ecdysis behavior [51,52]. Thus, it is striking to notice that, in addition to EH, all the other participants to the ecdysis hormonal cascade—the ecdysis triggering hormone (ETH)/pleurin (see below), the crustacean cardioactive peptide (CCAP), and bursicon—have evolutionarily related peptides in oysters [20]. This feature is shared by a number of Bilateria, and an ancestral role in the regulation of major life cycle transitions has been recently proposed [53].

#### 2.4.3. Cragi-Nesfatin

Nesfatin-1 (Nucleobindin-2-Encoded Satiety and FAT-Influencing proteiN-1) was discovered in the hypothalamus of rats as an anorexia-inducing factor [54]. This large regulatory peptide also regulates the energy balance in rodents and fish [55]. In fish, Nesfatin-1 has inhibitory effects on the hypothalamo–pituitary–ovarian (HPO) axis and, in turn, on reproduction [56]. Nesfatin-1 is encoded in the N-terminal region of the Nucleobindin 2 precursor, whose processing also generates two other peptides (Nesfatin-2 and 3) of unknown function. Except for *Drosophila melanogaster* [57], and more recently echinoderms [50], Nesfatin-encoding transcripts have not been described outside the chordate lineage. The oyster precursor displays a high degree of homology with vertebrate and non-vertebrate precursors, including the conservation of the prohormone convertase cleavage recognition sites delimiting the three mature Nesfatin peptides (Figure 2, Supplementary Figure S1). Although the sequence of the N-terminal region of Nesfatin-1 appears less conserved, the mid-region supporting the activity of Nesfatin-1 in mice [58] is highly conserved in the oyster precursor. Our MS approach was not dedicated to the characterization of very long peptides, so we did not characterize any mature Nesfatin peptides. However, the identification of a 16-amino-acid-amidated peptide generated from the N-terminal moiety of Cragi-Nesfatin-1 could represent a biologically active fragment (Figure 2). Cragi-Nesfatin-encoded transcripts were higher in the VG and during larval development. A lower level of expression was recorded in almost all adult tissues.

#### 2.4.4. Cragi-Neuroparsin/IGFBP

Neuroparsins are cysteine-rich polypeptide hormones that play important regulatory roles during development and in reproduction in insects [59]. They were initially characterized molecularly from the *corpora cardiaca* of the locust *Locusta migratoria* [60]. Although the neuroparsin gene is absent in *D. melanogaster* [61], most arthropods seem to harbor it [62]. Outside arthropod species, neuroparsin homologs have been identified in annelids, as well as in most classes of mollusks except for bivalves [22]. The present study shows the existence of one neuroparsin precursor in *C. gigas* and one in *Patinopecten yessoensis.* Both precursors display the majority of the canonical cysteine residues of this family of peptides (Figure 2, Supplementary Figure S1). As already mentioned for arthropod neuroparsins, Cragi-neuroparsin also has a significant degree of sequence similarity with the N-terminal hormone-binding domain of vertebrate insulin growth factor binding proteins (IGFBP) [62]. Cragi-neuroparsin was chiefly expressed during larval development and in adult tissues, especially in the adductor muscle, the mantle, the male and female gonads, and at lower levels in the VG. This pattern of expression is consistent with a plausible role of oyster neuroparsin in the regulation of developmental and reproductive processes. In other respects, the high expression in the mantle is in line with the identification of the nacre protein Perlustrin in abalone (*Haliotis laevigata*), another mollusk member of the IGFBP family with binding affinity for vertebrate IGF [63].

#### 2.4.5. Cragi-Urotensin

Urotensin II (UII) was first purified from the caudal neurosecretory system of the teleost (*Gillichthys mirabilis*) [64]. It was subsequently characterized in the vertebrate lineage, where it exerts a large array of behavioral effects and regulates endocrine, cardiovascular, renal, and immune functions [65]. Lophotrochozoan representatives of UII NPPs were characterized more recently [19,66], establishing the origin of this signaling system prior to the split between deuterostomes and protostomes. The Cragi-UII precursor is organized like its mollusk and vertebrate counterparts, with a UII-related peptide positioned at the C-terminal end (Figure 2, Supplementary Figure S1). MS analysis identified peptides covering the entire precursor. The UII-related peptide was detected as 27- and 19-amino-acid forms both containing the two cysteine residues required for the formation of a disulfide bridge. Cragi-urotensin transcripts were mainly expressed in the VG, and the corresponding peptides could be released in the hemolymph or play a modulatory activity on neuronal circuits as described for the *Aplysia californica* UII [66]. Cragi-UII transcripts were also detected in the first stages of larval development and only in the female gonad and the digestive gland of adults.

#### 2.4.6. Cragi-Prokineticin

Prokineticins are vertebrate cysteine-rich cytokines involved in a variety of biological processes [67]. In non-vertebrates, astakine—a prokineticin (PK) domain containing protein promoting differentiation and growth of hemopoietic stem cells in vitro—was discovered in crayfish (*Pacifastacus leniusculus*) [68]. This hematopoiesis-promoting activity was also observed in shrimp (*Penaeus monodon*) [69] and *C. gigas* [70]. Only one sequence—Cragi-prokineticin-1—was identified in the VG transcriptome of *C. gigas*. Four related protein-encoding-transcripts were found in the transcriptomes of different developmental stages or adult tissues. All sequences but Cragi-prokinecitin-5 exhibited a signal peptide sequence, suggesting that they are secreted (Figure 2). Alignment of the sequences with arthropod astakines and vertebrate prokineticins clearly showed that the ten cysteine residues implied in the formation of disulfide bonds and conferring a compact 3D structure were conserved [71] (Supplementary Figure S1). In contrast, non-vertebrate astakine/prokineticin precursors do not harbor the conserved N-terminal hexapeptide (AVITGA) that is crucial for bioactivity among vertebrates. Since this N-terminal hexapeptide is essential for the correct binding of prokineticins to their cognate receptors [72], non-vertebrate astakines probably operate distinctly. Consistently, it has to be mentioned that no phylogenetically related prokineticin receptor has been identified in protostomes [73]. Nevertheless, the completely unrelated membrane protein—the F1ATP synthase beta-subunit—was proposed as a potential receptor for astakine [74]. All oyster transcripts were expressed in the digestive gland and to a lesser extent in the male gonad. During development, a peak of expression was recorded in D-shaped and umbo larvae, a pattern consistent with the appearance of functional hemocytes [75]. Although Cragi-prokineticin-1 was slightly expressed in the VG, perhaps it was not of a neural origin; maybe it originated from the connective tissue sheaths or from contaminated VG-neighboring tissues.

#### 2.4.7. Cragi-Trunk/PTTH

The prothoracicotropic hormone (PTTH) is a peptide neurohormone that was molecularly identified for the first time in the silk moth (*Bombyx mori*). PTTH induces the synthesis and the secretion of the steroid hormone 20-hydoxyecdysone from the prothoracic glands and stimulates the molting process [76]. PTTH appears as an arthropod-specific NP, but its paralogous extracellular signaling molecule Trunk is widely distributed among metazoans, including lophotrochozoan and Deuterostomia [77], but also cnidaria and Ctenophora species [51]. In *D. melanogaster*, Trunk signaling is responsible for the specification of the most anterior and posterior regions of the embryo [78]. Both PTTH and Trunk signal via the same tyrosine kinase receptor called Torso [79]. This signaling pathway is also implicated in other processes such as the regulation of body size via the control of insulin signaling [80] and in light avoidance in *D. melanogaster* [81]. PTTH and Trunk precursors typically comprise a signal peptide and an N-terminal domain separated from the C-terminal cysteine-rich mature peptide by a proteolytic cleavage site [78,82]. We observed two types of precursors of the oyster PTTH/Trunk precursor homologs. Cragi-PTTH1 and 2 precursors displayed a signal peptide followed by the cysteine-rich mature peptide, while Cragi-PTTH 3 and 4 precursors had an additional peptide sequence with potential dibasic proteolytic cleavage sites (Figure 2, Supplementary Figure S1). Cragi-PTTH1 and 2 were exclusively expressed in the VG, though at higher levels for Cragi-PTTH2. In addition to the VG, Cragi-PTTH3 and 4 were highly expressed in most adult tissues, and were expressed across most developmental stages.

### 2.5. Protostome Families

#### 2.5.1. Cragi-Fulicin

**Figure 3:**
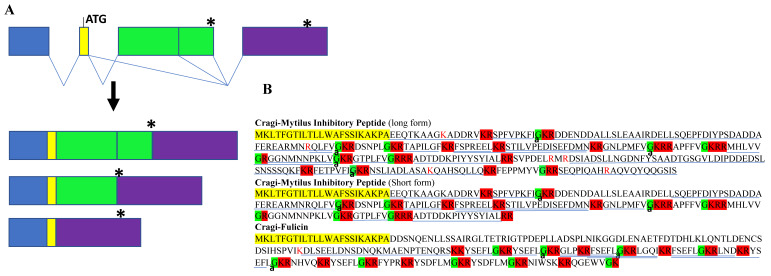
Fulicin /Mytilus inhibitory peptide in *C. gigas*: A. Organization of the gene (LOC105328755, linkage group 8, cgigas_uk_roslin_v1) and the three mature mRNAs generated by alternative splicing. * indicates a stop codon. B. Amino acid sequence of the three NPPs. Cragi-Mytilus Inhibitory Peptide (long form): CGG_contig_16327 and XP_034305796.1; Cragi-Mytilus Inhibitory Peptide (Short form): CHOYP_PRQFV.1.2 and XP_011428065.1; Cragi-Fulicin: XP_034305797.1. Colors and symbols as in Figure 2. Underlined are the peptides detected by MS and the C-terminal amide (a) is indicated.

This molluscan pentapeptide containing a D-amino acid residue was first purified from the central ganglia of the African giant snail (*Achatina fulica Ferussac*) [83]. Fulicin-related peptides (EFLGa peptides) have also been characterized in annelids [10,12]. One *C. gigas* contig containing the sequence of fulicin peptides was retrieved in this study. Oddly enough, this contig had an open reading frame encoding a Mytilus inhibitory-related peptide (PxFV/Iamide peptide) precursor [84], while the sequence encoding the fulicin precursor was out of frame in the 3′ untranslated region. The screening of the genome led to the identification of a gene (LOC105328755) characterized by a complex maturation of its primary mRNA (Figure 3). The use of an alternative 3′splice junction or the skipping of an exon generated three transcripts: two of them encoded a long and a short MIP/peptide precursor, while the third one encoded the precursor of fulicin, as in the *Capitella teleta* EFLGamide-encoding gene, the annelid ortholog of fulicin [12]. The Cragi-fulicin precursor encoded peptides with the (F/Y)S(E/D)FL(M/ø) amide signature, as well as one copy of QGEWVamide. Most of these peptides and their extended forms corresponding to partially processed peptides were detected by mass spectrometry, confirming the expression of the fulicin-encoding transcripts in the VG. Although their sequence differed from the sequence of the vertebrate thyrotropin-releasing hormone (TRH), orthologous peptides of annelid fulicin (EFLGamide) represent annelid TRH orthologs that specifically bind to the annelid ortholog of thyrotropin-releasing hormone receptors [85]. In *Caenorhabditis elegans*, the fulicin-related peptides (G/A)(R/N)ELFamide also activate a TRHR ortholog to promote growth and play a role in the regulation of reproduction [86]. Protostomian peptides are also present in a number of ecdysozoan species (Supplementary Figure S1). They share a common core signature E-[L/F]-[L/F/V] that is distinct from the signature of Deuterostomian TRHs [7]. In gastropod mollusks, fulicin appears to play a role in male copulatory behavior [83] and female egg-laying behavior [87].

#### 2.5.2. Cragi-GNQQN 

**Figure 4:**
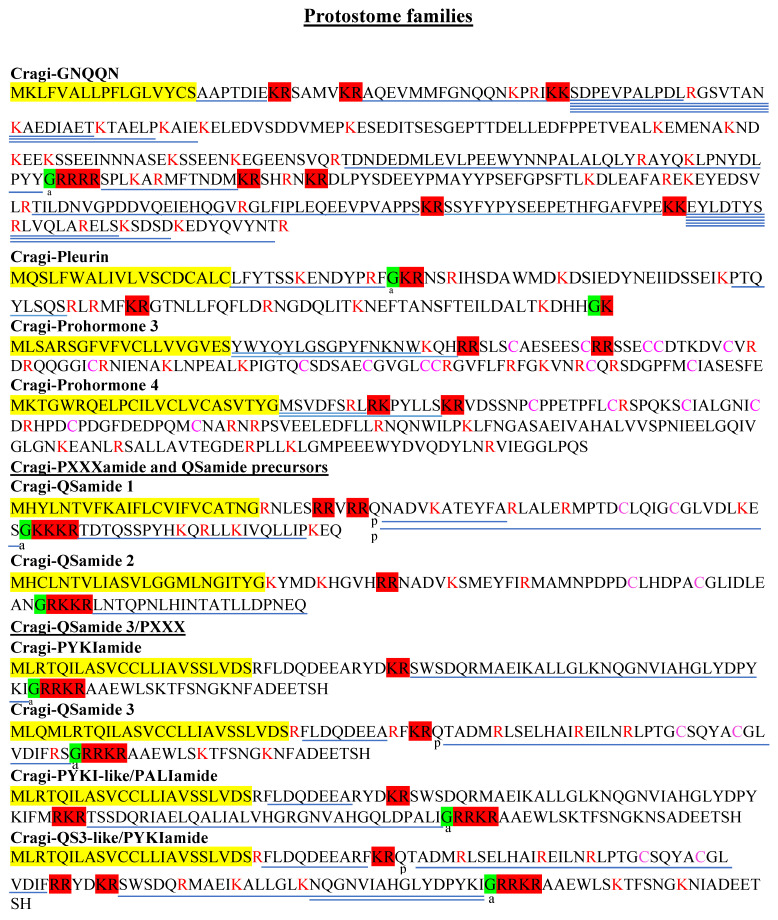
Novel protostome NPP types characterized in *C. gigas*. Colors and symbols as in Figure 2.

GNXQN NPPs were first described in annelids, bivalves, and gastropods [10,21]. Recent findings established a close evolutionary relationship between insect prohormone-2 and GNXQN precursors, pointing the origin of these families back to the last common protostomian ancestor [51]. Honeybee (*Apis mellifera*) prohormone-2 harbors the QNQQN sequence feature but shows no conservation of the cleavage sites and only limited sequence homology with the lophotrochozoan precursors (Supplementary Figure S1). The Cragi-GNQQN NPP also encodes a series of other peptides that we characterized by MS analysis, including a large amidated peptide also present in the other bivalve precursors, but sharing only limited sequence identity (Figure 4, Supplementary Figure S1). Cragi-GNQQN transcripts were mostly expressed during larval development and at much lower levels in most adult tissues, including the VG.

#### 2.5.3. Cragi-Pleurin

Initially identified in the snail *Helix lucorum* as a neurotransmitter mediating the withdrawal reaction [88], the corresponding NPP in *A. californica* was named pleurin owing to its localized expression in the right pleural ganglia [89]. Orthologous NPPs have also been found in *Lottia gigantea* and in the gray garden slug *Deroceras reticulatum* [16]. The *L. gigantea* precursor encodes four copies, whereas the *A. californica* and *D reticulatum* precursors contain three copies of the mature NPs characterized by a C-terminal PRXamide sequence. In bivalve mollusks, both Cragi-pleurin and *P. yessoensis* [21] precursors only predict one copy of the PRXamide peptide (Figure 4). In contrast to other species, in which the “X” amino-acid is (I/L/V/M) amide, the oyster NP ends by an Famide, and thus represents a new member of the RFamide family of NPs in mollusks [90]. Cragi-pleurin exhibited only a very low level of expression in the VG. Pleurin-encoding sequences have also been found in the annelid *Capitella teleta* and in *Alvinella pompejana* nucleotide sequence databases [73]. Similarity searches showed that pleurin peptides also share a PRX amide signature with the ecdysozoan ecdysis-triggering hormone (ETH) [73]. The ETH precursor also had a similar organization, especially regarding the position of the PRX peptide sequence right after the signal peptide. Since ETH receptors have been phylogenetically identified in mollusks [51], it would be relevant to investigate their functional connection with pleurin. 

#### 2.5.4. Cragi-Prohormone 3 

A peptidomic survey of honey bee *A. mellifera* brain identified a new peptide [ITGQGNRIF] encoded at the C-terminal end of the prohormone-3 precursor [91], also named ITG-like prohormone. Proteins displaying similarities to this precursor are present in arthropods, although they do not all contain the ITGQGNRIF-related peptide detected in honeybee samples. The Cragi-prohormone 3 transcript encoded a signal peptide, two short peptides that were detected by MS, and an 84-amino-acid cysteine-rich peptide (Figure 4). In contrast to the annelid sequence, the oyster and scallop sequences only shared 12 of the 16 conserved cysteine residues with other arthropod prohormone-3 (Supplementary Figure S1). Cragi-prohormone-3 transcripts were mainly expressed in the VG and in a few adult tissues such as the mantle, the gills, and the gonads.

#### 2.5.5. Cragi-Prohormone-4

Prohormone-4 was first identified after the characterization of a new de novo sequenced peptide [IDLSRFYGHFNT] from honey bee (*A. mellifera*) brain [91]. The IDLSRFYGHFNT-containing precursor is overall well conserved in arthropods [92]. Corresponding precursor sequences were recently characterized in the transcriptomes of the venom gland of marine cone snails [93], as well as in other mollusk species [22]. The *C. gigas* prohormone-4 precursor exhibited a typical organization with an N-terminal signal peptide flanked by two predicted short peptides, an LDL-receptor class A domain containing six conserved cysteines, followed by a long C-terminal cysteine-free peptide (Figure 4, Supplementary Figure S1). MS analysis identified the two short predicted peptides [MSVDFSRL] and [PYLLS]: the first one is the counterpart of the original *A. mellifera* peptide [IDLSRFYGHFNT], and the second one only occurs in lophotrochozoan precursors. Although the oyster transcripts were found in most adult tissues, they were high in the mantle and in the VG, further confirming the NP nature of these peptides. No difference was found between the male and female nervous systems, in contrast with the situation in the lobster *Sagmariasus verreauxi* [92]. In *A. mellifera*, the mature peptide was differentially abundant in the brains of pollen and nectar foragers [94], suggesting the involvement of prohormone-4 peptides in behavior and/or food intake [95]. Prohormone-4 also appears highly related to task transition of honeybee workers [96].

#### 2.5.6. Cragi-PXXXamide and QSamide Precursors

**Figure 5:**
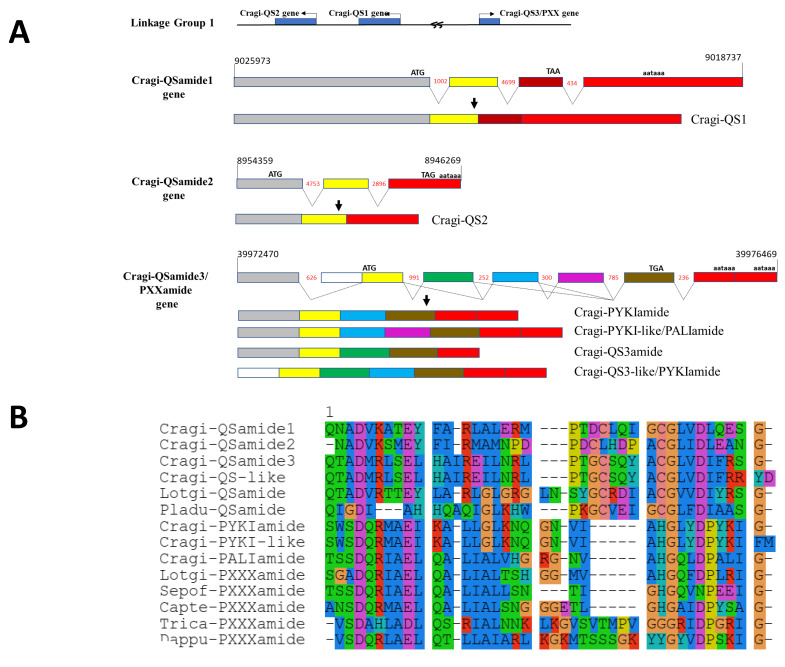
Organization of the QSamide /PXXXamide encoding genes in *C.gigas*: A. Cragi-QSamide1, Cragi-QSamide2, and Cragi-QSamide3/PXXXamide genes and the mature mRNA produced. B. Alignment of mature QSamide and PXXXamide neuropeptide sequences of *C. gigas* with related sequences from other species. Amino acids with similar physicochemical properties are of the same color. Capte: *Capitella teleta*, Dappu: *Daphnia pulex*, Lotgi: *Lottia gigantea*, Sepof: *Sepia officinalis*, Trica: *Tribolium castaneum*.

The PXXXamide precursor was first characterized from cuttlefish (*Sepia officinalis*), and homologous precursors were subsequently identified in a set of protostome species [19]. The main mature peptide shares the C-terminal PXXXamide signature with its relatives. Interestingly, this family of peptides also exhibits some homology with the QS peptide family initially characterized in the annelid *Platynereis dumerlii* [10] (Figure 5), and had only been identified in Lophotrochozoa until now. As the PXXXamide precursors and QSamide precursors also exhibit a similar organization, the common origin of these two families is reasonably well founded. The relationship between these two families was further illustrated in *C. gigas* by examining the organization of the genes encoding this variety of precursor. Although only two genes encoded QSamide peptide types (Cragi-QSamide 1 and CragiQSamide 2), another gene (the Cragi-QSamide 3/PXXXamide gene) located on the same chromosome showed a complex pattern of differential splicing of a single precursor mRNA, leading to transcripts encoding either a QSamide peptide (Cragi-QSamide3) or a PXXXamide peptide (Cragi-PYKIamide, Cragi-PYKI-like, and Cragi-PALIamide or Cragi-QS3-like and Cragi-PIKIamide) (Figure 4 and Figure 5). Overall, the three genes and the different transcripts—except Cragi-QSamide 2 and Cragi-PIKI-like/PALIamide—were detected in the VG and expressed at similar levels (Supplementary Figure S1). Cragi-QSamide 1 was predominantly expressed in the larval developmental stages. Cragi-QSamide 3, Cragi-PYKIamide, and Cragi-QS3-like/PYKIamide, were expressed too, but at much lower levels. In adult tissues, all transcripts but Cragi-PYKI-like/PALIamide were mainly expressed in the digestive gland and the gonads. All mature peptides but Cragi-QSamide2 were detected by MS, reflecting the relative level of expression of the transcripts in the VG. The biological function of QSamide and PXXXamide is still unknown. A PXXXamide was recently found to activate an ortholog of a vertebrate parathyroid hormone receptor (PTHR) in the red flour beetle (*Tribolium castaneum*) [97]. Considering that a PTHR ortholog is female-gonad-specific in *C. gigas* [98], the oyster PXXXamide/PTHR signaling system can be expected to regulate some key steps of female gametogenesis, with possible major outcomes in terms of female fecundity.

### 2.6. Lophotrochozoan Families

**Figure 6:**
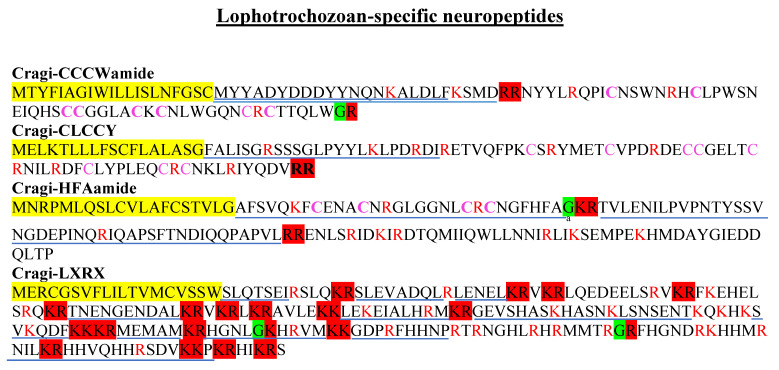
Novel lophotrochozoan NPP types characterized in *C. gigas*. Colors and symbols as in Figure 2.

New oyster NPPs previously characterized in *P. dumerilii* were clearly identified. They are considered as Lophotrochozoan-specific NPs because they are only found in annelid, mollusk, or platyhelminth phyla [10]. They included Cragi-CCWamide, Cragi-CLCCY, Cragi-HFAamide, and Cragi-LXRX (Figure 6). Although this latter NPP was highly and almost strictly expressed in the VG, the other ones displayed only a low expression in the VG. Cragi-CCWamide was mainly expressed in the digestive gland and in post-metamorphosis juveniles. Cragi-HFA appeared specifically expressed in the larval stages (Supplementary Figure S1), and at much lower levels in the mantle.

### 2.7. Mollusk Families

**Figure 7:**
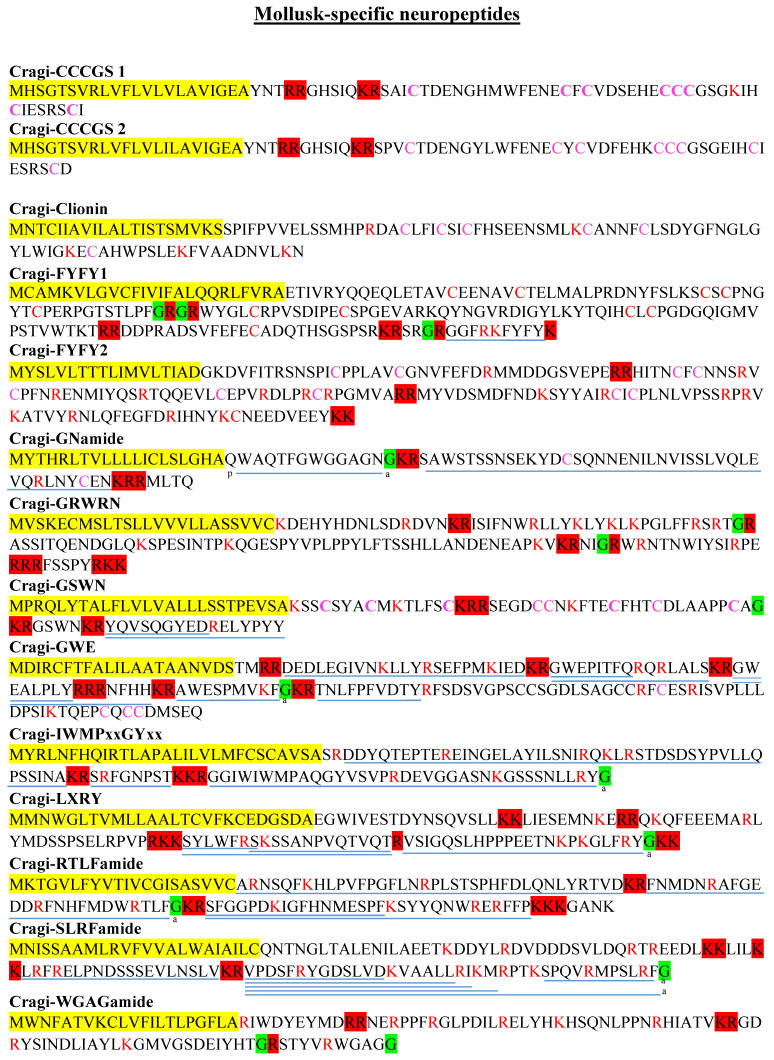
Novel mollusk-specific NPPs characterized in *C. gigas*. Colors and symbols as in Figure 2.

Cragi-CCCGS. Alternative spliced transcripts of a single gene (LOC105346269) code for two cysteine-rich secreted peptides only characterized in *C. gigas* so far. The Cragi-CCCGS sequences made up almost 20% of the mature peptides, and contained 8 cysteine residues out of 42 amino acids (Figure 7). Compared to most NPP transcripts, Cragi-CCCGS mRNA had an extremely low level of expression in the VG. Cragi-CCCGS2 transcripts had higher levels in the female gonad, the digestive gland and the mantle, and only in D larvae and post-metamorphosis juveniles during development. No related sequence was identified in any other species. Some secreted cysteine-rich peptides display antimicrobial [99,100] or neurotrophic activity [101]. We cannot rule out that Cragi-CCCGS peptides play such roles and are induced only after microbial challenge or injury. 

Cragi-clionin. Clionin precursor was initially identified from the gastropod mollusk *Tritonia tetraquetra* (ABU82762) (unpublished data). This cysteine-rich peptide was further characterized in the cephalopod mollusk *S. officinalis*, where its encoding transcript was strictly expressed in mature oocytes [19]. In *C. gigas*, Cragi-clionin was moderately though exclusively expressed in the VG, and slightly more expressed in stage-3 males (Figure 7 and Supplementary Figure S1).

Cragi-FYFY. This NPP was first detected from the transcriptome of the scallop (*P. yessoensis*) nervous system [21]. It potentially yields a long mature peptide, displaying 10 cysteines and a C-terminal peptide with the FYFY motif. We found two related NPPs in *C. gigas* and one in *L. gigantea.* The cysteine pattern of the long peptide was strictly conserved among the different mollusk sequences. In contrast, the terminal FYFY peptide was only predicted in the Cragi-FYFY1 precursor but not in the Cragi-FYFY2 precursor (Figure 7 and Supplementary Figure S1). Interestingly, the corresponding peptide in the *L. gigantea* precursor had 41 copies but only displayed the FYmotif. The mature oyster C-terminal FYFY peptide was detected by MS, with low expression of Cragi-FYFY1 in the VG compared to Cragi-FYFY 2. Moreover, Cragi-FYFY 2 was also detected in the mantle and during larval development. 

Cragi-GNamide. This NPP was first found in scallop (*P yessoensis*) [21]. It had only one related sequence in *C. gigas* and may represent a bivalve-specific NP (Figure 7). In *C. gigas*, Cragi-GNamide transcripts were only detected in the VG (Supplementary Figure S1). The Cragi-GNamide precursor encoded two peptides: pQTFGWGGAGNamide and a 41-amino-acid peptide. Both peptides were detected by MS (Figure 7).

Cragi-GRWRN. This NPP only shows homologies with a *C. virginica* sequence, and with the V-amide precursor sequence of *P. yessoensis* to a lesser extent [21] (Figure 7 and Supplementary Figure S1). Intriguingly, the mature V-amide peptide was not present in oyster precursors, but the GRWRN sequence was found conserved in all three precursors. The Cragi-GRWRN gene was weakly and quasi exclusively expressed during development. Significantly higher expression was observed in the VG of stage-3 males and females. 

Cragi-GSWN. This newly characterized precursor is defined by a 16-amino-acid C-terminal peptide detected by MS, a potential cysteine-rich amidated peptide, an N-terminal cysteine-rich peptide, and a short GSWN peptide (Figure 7). Homologous precursor sequences have been found in the bivalve mollusks *C. virginica* and *P. yessoensis,* as well as in the gastropod mollusks *L gigantea* and *A. californica*. Sequence alignment emphasized the strict conservation of the position of the cysteines and of the GSWN sequence. The Cragi-GSWN gene was weakly expressed in the VG (Supplementary Figure S1).

Cragi-GWE. This precursor encodes four N-terminal peptides, three of which start with the “G/AWE” motif, as well as a single C-terminal cysteine-rich peptide (Figure 7). A related precursor named GW has been characterized in *P. yessoenssis* [21]. However, in contrast with its *C. gigas* and *C. virginica* counterparts, it has a distinct organization and only partial identity, except for the “GW” motif (Supplementary Figure S1). Cragi-GWE transcripts were expressed in the VG and in most adult tissues and larval stages, but at lower levels. Most predicted mature NPs were detected by MS.

Cragi-IWMPxxGY. This NPP was initially identified in scallop *P. yessoensis* [21]. In *C. gigas*, all the predicted mature peptides were characterized by MS, including the C-terminal peptide harboring the IWMPxxGY and F/LRYamide features (Figure 7). The Cragi-IWMPxxGY gene was moderately expressed in the VG and during development. The male gonad, and to a lesser extent the female gonad, and the digestive gland also expressed the corresponding gene (Supplementary Figure S1).

Cragi-LXRY generates a 23-amino-acid C-terminal amidated sequence that we detected by MS, and potentially four other peptides (Figure 7). It represents a homolog of *P. yessoensis* LRYamide [21] and shares the LXRYamide sequence with it (Supplementary Figure S1). The encoding gene was only expressed in the VG. No homologous precursor was found in other mollusks. 

Cragi-RTLFamide represents a new precursor generating three/four main mature peptides all detected by MS (Figure 7). Only one related NPP was retrieved from *L. gigantea* sequences. Sequence homology was mainly restricted to the N-terminus of the oyster 24-amino-acid (RTLF) amidated peptide, but the corresponding *L. gigantea* peptide had a distinct C-terminus and was not amidated (Supplementary Figure S1). Cragi-RTLFamide transcripts were mainly expressed in the VG, in the mantle, and during the larval stages. All mature peptides predicted from the oyster precursor were characterized by MS.

Cragi-SLRFamide. This NPP is a new member of the RF family of NPs. It appears to be specific to bivalve mollusks, as no related sequence has been identified from sequence resources of other mollusks yet. In *C. gigas*, this arginine-rich precursor protein generates multiple truncated peptides detected by MS, including the C-terminal SLRFamide peptide that displays high sequence identity across the different bivalve precursors (Figure 7 and Supplementary Figure S1). The Cragi-SLRFamide gene was mostly expressed during larval development and at lower levels in adult tissues, including the VG.

Cragi-WGAGamide. Its precursor potentially encodes four NPs (Figure 7). The two possibly amidated C-terminal peptides are highly similar to the *C. virginica* and *P. yessoensis* C-terminal peptides (Supplementary Figure S1), but none of these peptides were detected by MS. The encoding transcripts were only expressed at a very low rate in the VG. This possibly explains the absence of detection of mature peptides in VG extracts.

### 2.8. Differentially Expressed NPP Transcripts during a Reproductive Cycle

**Figure 8:**
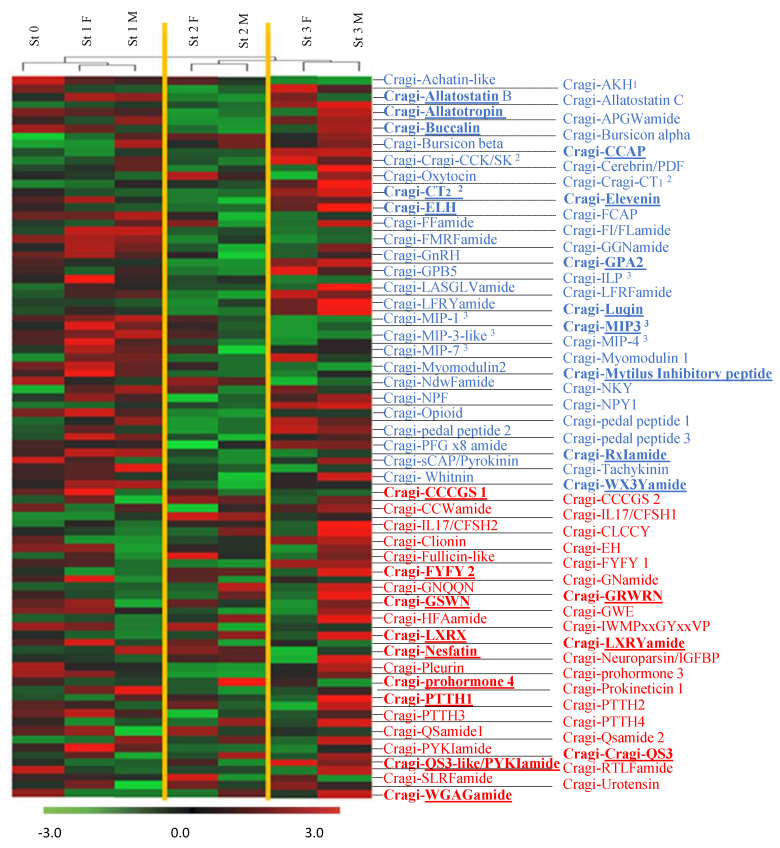
Heat map of transcripts encoding NPPs expressed over a reproductive cycle in the visceral ganglia. Three sample branches are observed, mainly clustering stage 0/1, stage 2, and stage 3. The variations in transcript abundance are specified with a color scale, in which shades of red represent higher transcript expression and shades of green represent lower transcript expression. St3: stage 3; St2: stage 2; St1: stage 1; St0: stage 0. F: Female, M: Male. In blue, the neuropeptide precursors already characterized in *C. gigas* [20] or recently investigated: ^1^: [25], ^2^: [28,30], ^3^: [24,25]. In red, the newly characterized NPPs. Bold and underlined are the differentially expressed neuropeptide encoding transcripts.

**Figure 9:**
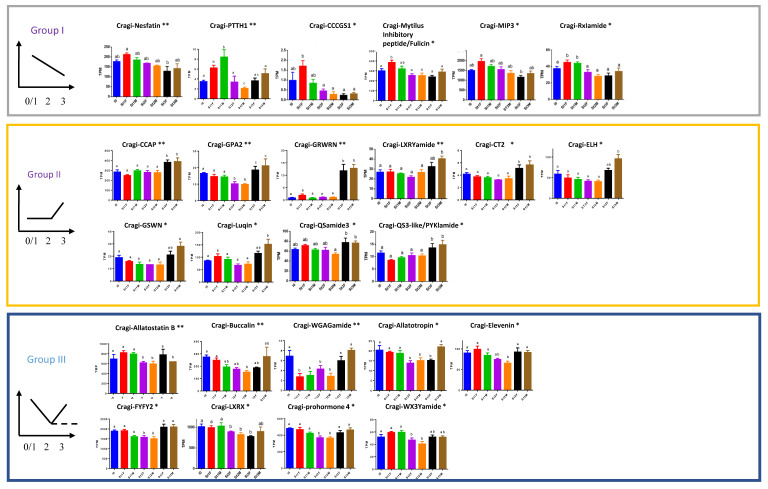
Expression profile of differentially expressed neuropeptide precursor encoding transcripts over the reproduction cycle in male and female oysters. Each value is the mean +SEM. Results were statistically tested using a one-way ANOVA, * *p* < 0.05 or ** *p* < 0.01 using software R. Samples with significant statistical difference are marked with distinct letters.

A hierarchical clustering was performed on VG samples for the full collection (96) of characterized *C. gigas* NPP genes based on their relative expression levels. Three major clusters of samples were defined corresponding to the first stages of gametogenesis (stage 0, female and male stage 1), male and female stage 2, and male and female at the mature and spawning stage (stage 3) (Figure 8). No sex-biased differential expression of the NPP-encoding genes was observed in the whole set of genes. A focused statistical analysis of the expression levels of the 96 NPP transcripts finally identified 25 differentially expressed NPPs throughout a complete reproductive cycle (Figure 9). These NPPs were sorted into three main groups as follows: group I gathered the NPPs with the highest expression at the start of the reproductive cycle (stages 0 and 1), followed by a continuous decline until stage 3; group II gathered the NPPs with the highest expression at the mature and spawning stage (stage 3); group III gathered the NPPs displaying the lowest expression at stage 2. A high expression of a given NPP in the VG at a given reproductive stage means that it is involved in the positive or negative regulation of gametogenesis or in the energy balance occurring during this stage, but does not provide any evidence about the way it is implied. NPs can indeed work as neurotransmitters/neuromodulators to control the release of neurohormones, or to participate to functional neural circuits that control feeding and the nutritional balance; this way, they indirectly affect the energy reserves allocated to reproduction. They may also directly regulate target tissues, but neural projections from the VG to the gonads have not been confirmed. Alternatively, NPs can be released in the circulatory system and, as neuroendocrine factors, they regulate the activity of distant target tissues. In this case, the expression of the neurohormone or its specific receptors in the gonadic tissues would represent an additional indicator of a role of the given NP in the regulation of reproduction.

### 2.9. NPPs Differentially Expressed in the First Stages of Gametogenesis

It is particularly interesting to note that NPs known in other species to regulate the energy balance, such as Nesfatin [55], to exert a dual control of the nutritional status and reproductive effort in oyster (Cragi-MIP3) [24,102], or to be involved in the control of insulin signaling (PTTH) [80] were upregulated in group I of differentially expressed NPPs. These stages are characterized by the replenishing of the storage tissues. In parallel, stage 1 corresponds to a period of germ line proliferation, which is controlled by insulin signaling in some invertebrate species [103,104]. With respect to its suggested potential growth factor activity, CCCGS1 may also be involved in germ cell proliferation. Among the other NPPs differentially expressed at these stages, Cragi-*Mytilus* inhibitory peptide (MIP)/fulicin precursor-derived peptide family members have been identified as reproduction-associated peptides. They regulate the contraction of the penis retractor muscle [83,84,105] and the female reproductive tractus [87] of the snail *Achatina fulica*. A role of the fulicin and MIP/FVamide peptides in reproduction has also been postulated in other animal phyla [86]. The bivalve Cragi-RxIamide peptide, with no obvious similarity to known peptides [20], also appears to contribute in reproduction-associated processes.

### 2.10. NPPs Differentially Expressed in the Mature Spawning Stage

Group II NPPs were more expressed in stage 3, corresponding to the final step of the gamete maturation process and to spawning. This group included Cragi-CCAP (Crustacean Cardioactive Peptide), Cragi GPA2 (Glycoprotein Hormone α subunit), Cragi-GRWRN, Cragi-LXRYamide, Cragi-CT2 (Calcitonin), Cragi-ELH (Egg-Laying Hormone), Cragi-GSWN, Cragi-Luqin, Cragi-QSamide3, and Cragi-QS3like/PYKIamide (Figure 9). In *C. gigas*, the two Cragi-CCAP and Cragi-CT2 signaling systems play a role in water and ionic balance by regulating the activity of the gills [29,30]. Their involvement in the regulation of gill cilia activity could explain their contribution to spawning [1]. Interestingly, in other mollusks, CCAP triggers spawning in the Sydney rock oyster (*S. glomerata*) [106] and controls oocyte transport and egg-capsule secretion in cuttlefish (*S. officinalis*) [107]. That Cragi-ELH is involved in spawning appears rational given the role of this NP as an ovulation hormone in gastropod mollusks [108,109]. However, no ELH activity has been investigated in other mollusk classes yet. Intriguingly, no ELH-related sequence has been retrieved from cephalopod databases [19,22], suggesting that ELH might not be crucial for mediating the emission of gametes in non-gastropod mollusks. As ELH represents a homolog of chordate corticoliberin (CRH) and of ecdysozoan diuretic hormone 44 (DH44), Cragi-ELH could contribute to stress mediation or water and ion regulation. Cragi-GPA2 and Cragi-GPB5 form a potential heterodimer and are members of the glycoprotein hormone family [110]. GPA2/GPB5 signaling is possibly involved in ionoregulation and osmoregulation in mosquitos [111] or development in *Aplysia californica* [112] and *D. melanogaster* [113]; it also controls germline development and fertility in *C. elegans* [114] and reduces male fertility in *Aedes aegypti* [115]. In *C. gigas*, only GPA2 was differentially expressed in the gonads of both males and females, in contrast with its higher expression in the testes of male flies [113]. The absence of coregulated expression of the partner subunit transcripts was also found during development in *D. melanogaster* [113], suggesting that each subunit may work independently or form homodimers. However, this hypothesis was recently set aside [116]. Consistent with the increased level of Cragi-Luqin during stage 3, Luqin negatively regulates the egg-laying producing cells in *Lymnaea stagnalis* [117], and Luqin immunoreactive fibers have been detected in the genital ganglion of *A. califormica* [118]. Likewise, the contribution of Cragi-QS3-like/PYKIamide to reproduction appears coherent given its expression in the gonads (Supplementary Figure S1) and the specific expression of its putative cognate receptor [98] in the gonads of female oysters. The expression patterns of the newly characterized peptides Cragi-GRWRN, Cragi-LXRYamide, Cragi-GSWN, and Cragi-QSamide3 also select theses NPs as interesting candidates to mediate reproduction or associated processes during stage 3.

### 2.11. NPPs Expressed in the First Stages of Gametogenesis and in the Mature Spawning Stage

Group III NPPs were characterized by decreased expression in both males and females during stage 2. This decrease may have lifted inhibitory activities and allowed for the maturation process of the gametes to be initiated during stage 2. On the other hand, NPPs may play a dual role at the spawning period and during the first stages of a novel reproductive cycle. Of the various NPPs differentially expressed in group II, some are known to mediate reproductive functions. Buccalin peptides induce spawning and promote gonad development in *S. glomerata* [106]. Allatostatin B/ Myoinhibitory peptides exhibit a muscle modulatory activity, are implied in a variety of feeding-related functions in insects, and play a role in their reproductive system [119] (for a review). In addition, the knocking down of the allatostatin B/ myoinhibitory peptide gene resulted in a moderate reduction in egg-laying in *D. melanogaster* [120]; this gene could be involved in the regulation of egg laying in *S. officinalis* [19]. Allatotropin is expressed in male and female gonads of the fall armyworm (*Spodoptera frugiperda*), suggesting a role in reproduction [121]. Despite their presence in a variety of mollusks, no biological function has been assigned to this family of peptides yet. Regarding the other NPPs in group III, there is a paucity of reports on the physiological functions of the widely distributed protostome peptide Elevenin [12,19,20,122,123], and on those of the lophotrochozoan peptide LXRX and the molluscan peptides WGAGamide and Wx3Yamide. Prohormone-4 stands apart, and could be implied in food intake by the honeybee (*A. mellifera*) [95]. The present study constitutes a first hint of their possible involvement in the mediation of reproduction and associated functions in mollusks. 

## 3. Materials and Methods

### 3.1. Animal and Tissue Sampling

**Figure 10:**
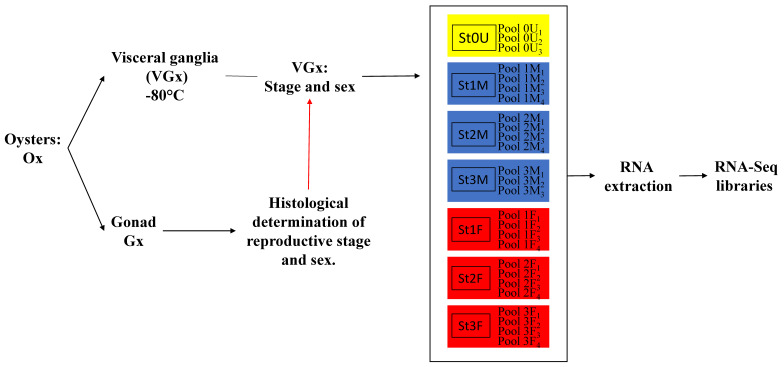
Sampling protocol: For each individual oyster (Ox), the gonad (Gx) and the VG (VGx) were dissected out. VGx were stored at −80 °C until use. To establish the sex and the reproduction stage, Gx was histologically examined according to Rodet et al. [124]. Thus, VGx was associated with a reproduction stage and a sex. Pools of VGx from 6 animals of the same stage and the same sex were generated: 3 pools for sexually undifferentiated stage 0 (St0U); 4 pools for male stages 1 and 2 (St1M, St2M); 3 pools for male stage 3 (St3M); and 4 pools for female stages 1, 2, and 3 (St1F, St2F, St3F). Total RNA was extracted from each pool and RNA-Seq libraries were constructed.

Two-year-old adult oysters, *C. gigas*, were purchased from a local oyster farm (Normandie, France). Visceral ganglia (VG) were dissected out from animals at different reproductive stages. All the collected samples were individually placed or stored at −80 °C until use. For differential expression studies, visceral ganglia collected from 6 animals of the same sex and the same reproduction stage were mixed to generate 26 pools, as described (Figure 10). The minimum number of replicates per conditions was chosen in order to allow statistical tests to be carried out. Reproduction stages were determined by histological analysis of gonad sections as described by Rodet et al. [124], according to Lubet’s classification [125]. The first stage (stage 0) corresponds to the sexual resting stage; at this stage the sex of an individual cannot be determined as only small clusters of self-renewing stem cells can be observed scattered in the vesicular conjunctive tissue (VCT). Stage 1, corresponding to gonial multiplication, is characterized by poorly developed gonadal tubules surrounded by a large matrix of vesicular connective tissue. Stage 2 corresponds to the gonadic maturation stage with the development of the tubules and the regression of VCT. In males, all germline cells can be observed (from spermatogonia to spermatozoa); in females, oocytes are blocked in prophase I and vitellogenesis occurs; oocytes of different sizes are present. Stage 3 is the sexual maturity stage, with the gonad showing its maximum size with tubules full of mature germinal cells.

### 3.2. RNA Extraction 

Tissue samples in Tri-reagent TM (Ref: 93289, Sigma-Aldrich, St. Louis, MO, USA) were scattered and homogenized using a syringe (0.9 mm). Total RNA was isolated according to the manufacturer’s instructions. The recovered RNA was then further purified on Nucleospin RNAII columns (Ref: 740955.50S Macherey-Nagel, Hoerdt, France).

### 3.3. Illumina Sequencing 

Library construction and sequencing were conducted at the Genome Quebec Innovation Center (McGill University, Montréal, QC, Canada).

Total RNA was quantified using a NanoDrop Spectrophotometer ND-1000 (NanoDrop Technologies, Wilmington, DE, USA) and its integrity was assessed on a 2100 Bioanalyzer (Agilent Technologies). Libraries were generated from 250 ng of total RNA as follows: mRNA enrichment was performed using the NEBNext Poly(A) Magnetic Isolation Module (New England BioLabs, Ipswich, MA, USA). cDNA synthesis was achieved with the NEBNext RNA First Strand Synthesis and NEBNext Ultra Directional RNA Second Strand Synthesis Modules (New England BioLabs, Ipswich, MA, USA). The remaining steps of library preparation were conducted using the NEBNext Ultra II DNA Library Prep Kit for Illumina (New England BioLabs, Ipswich, MA, USA). Adapters and PCR primers were purchased from New England BioLabs. Libraries were quantified using the Kapa Illumina GA with Revised Primers-SYBR Fast Universal kit (Kapa Biosystems Roche, Basel Switzerland). Average size fragment was determined using a LabChip GX (PerkinElmer, Waltham, MA, USA) instrument. The libraries were normalized and pooled at 3nM and then denatured in 0.05N NaOH and neutralized using HT1 buffer. ExAMP was added to the mix following the manufacturer’s instructions. The pool, then at 360 pM, was loaded on a Illumina cBot (Illumina, San Diego, CA, USA) and the flowcell was run on a HiSeq 4000 for 2 × 100 cycles (paired-end mode). A phiX library was used as a control and mixed with libraries at 1% level. The Illumina control software was HCS HD 3.4.0.38 and the real-time analysis program was RTA v. 2.7.7. Program bcl2fastq2 v2.20 (Illumina, San Diego, CA, USA) was then used to demultiplex samples and generate fastq reads.

### 3.4. Mass Spectrometry Analysis

#### 3.4.1. Sample Preparation for Mass Spectrometry Analysis

Twenty visceral ganglia were extracted in 0.1% trifluoroacetic acid (TFA) at 4 °C and centrifuged for 30 min at 35,000× *g* at 4 °C. The supernatants were concentrated on ChromafixC18 solid phase extraction cartridges (Macherey-Nagel, Hoerdt, France). Then, the recovered samples were evaporated. For nano-LC fragmentation, peptide samples were first desalted and concentrated onto a µC18 Omix (Agilent, Technologies Co., Ltd., Palo Alto, CA, USA) before analysis. The chromatography step was performed on a NanoElute (Bruker Daltonics, Billerica, MA, USA) ultra-high pressure nano flow chromatography system. Approximatively 200 ng of each peptide sample was concentrated onto a C18 pepmap 100 (5 mm × 300 µm i.d.) precolumn (Thermo Scientific, Waltham, MA, USA) and separated at 50 °C onto a reversed phase Reprosil column (25 cm × 75 μm i.d.) packed with 1.6 μm C18-coated porous silica beads (Ionopticks, St, Fitzroy, VIC, Australia). Mobile phases consisted of 0.1% formic acid, 99.9% water (*v*/*v*) (A), and 0.1% formic acid in 99.9% ACN (*v*/*v*) (B). The nanoflow rate was set at 400 nL/min, and the gradient profile was as follows: from 2 to 15% B within 60 min, followed by an increase to 25% B within 30 min, and further to 37% within 10 min, followed by a washing step at 95% B and re-equilibration. 

#### 3.4.2. Mass Spectrometry Analysis

MS experiments were carried out on an TIMS-TOF pro mass spectrometer (Bruker Daltonics, Billerica, MA, USA) with a modified nano electrospray ion source (CaptiveSpray, Bruker Daltonics, Billerica, MA, USA). The system was calibrated each week and mass precision was better than 1 ppm. A 1400 spray voltage with a capillary temperature of 180 °C was typically employed for ionizing. MS spectra were acquired in the positive mode in the mass range from 100 to 1700 *m*/*z*. In the experiments described here, the mass spectrometer was operated in PASEF mode without exclusion of single charged peptides. A number of 10 PASEF MS/MS scans were performed during 1.25 s from charge range 1–8. 

#### 3.4.3. Peptide Sequencing and Protein Precursor Identification

The fragmentation pattern was used to determine the peptide sequence. Database searching was performed using the Peaks X+ software package (Bioinformatics Solutions Inc., Waterloo, ON, Canada). A homemade database corresponding to the whole set of formerly and newly identified NPPs in *C. gigas* was used.

The variable modifications allowed were as follows: C-Carbamidomethyl, methionine oxidation, N-terminal Q-pyroglutamate, and C-terminal amidation. No enzyme digestion was selected. Mass accuracy was set to 30 ppm and 0.05 Da for MS and MS/MS mode, respectively. Data were filtered according to an FDR of 0.1%.

### 3.5. In Silico Studies

#### 3.5.1. De Novo RNA-Seq Data Assembly 

RNA-seq data were assembled following two assembly strategies with the DRAP pipeline v1.91 [126]. The first strategy pooled raw datasets by sample and the 7 samples (F1, F2, F3, I0, M1, M2, and M3) were assembled independently with runDrap using Oases with kmers 25, 31,37, 43, 49, 55, 61, 65, 69. The individual contig sets filtered by FPKM (fragments per kilobase per million mapped reads) over one were then merged with runMeta and filtered again by FPKM over one to produce the reference contig set. The second strategy pooled raw datasets by stage and the 4 stages (stages 0 to 3) were assembled independently before being merged following the same procedure. The assembly metrics of both resulting contig sets were compared using runAssessment, the third DRAP module. With a number of contigs slightly higher but with best read mapping rates, there were a number of matching *C. gigas* proteins with 80% identity and 80% coverage and BUSCO metrics; the assembly by stage was therefore chosen as the reference contig set. The reference contig set was aligned on UniProt Swiss-Prot, RefSeq and protein databases (NCBI *Crassostrea gigas* GCA_000297895, NCBI *Lottia gigantea* GCF_000327385, NCBI *Octopus bimaculoides* GCF_001194135, Ensembl *Danio rerio* GRCz10, Ensembl *Homo sapiens* GRCh38) using BLASTX [127] for annotation, and processed with InterProScan [128] to collect structural and functional annotations. The read sets were realigned on the contigs with BWA-MEM version 0.7.12-r1039 (standard parameters) [129]. The contig read counts were generated using the BAM files with samtools idxstats version 1.3.1 (standard parameters) and merged into a unique expression file with Unix Bash commands. The BAM file was processes with GATK version 3.0-0-g6bad1c6 (standard RNA-Seq parameters) [130] in order to find variants. All annotations, variations, and expression measures were uploaded to RNAbrowse [131]. This transcriptome shotgun assembly (TSA) project has been deposited at DDBJ/EMBL/GenBank under the accession GIUV00000000, BioProject (PRJNA662446). The version described in this paper is the first version, GIUV01000000.

#### 3.5.2. Global Analysis of the Transcriptome

Short read archives (SRA) corresponding to RNA expressed in the VG of males and females at different stages of reproduction can be accessed under the accession number (PRJNA662446) https://www.ncbi.nlm.nih.gov/sra/PRJNA662446 (accessed on 6 August 2021). VG sample Reads were aligned to the *C. gigas* VG TSA (GIUV00000000) as reference transcriptome using BWA v0.7.12. Estimated read counts for each sequence were calculated by using the TPM (transcripts per kilobase per million reads) method to provide a normalized comparison of gene expression between all sample [132]. A principal component analysis (PCA) of the VG transcriptome data was applied using the FactoMineR package software (v.3.6.3) [133]. 

#### 3.5.3. Neuropeptide Precursor Searches 

To complete the *C. gigas* repertoire of NPPs [20,24,25,26,30], protostomian NPP protein sequences previously reported in the literature were used as queries in tBLASTn searches to identify homologous sequences in the *C. gigas* VG transcriptome database. Default parameters were used and an E-value threshold of maximum 1.3 was accepted, providing the target sequence displayed the features of NPP sequences (presence of a signal peptide, potential proteolytic cleavage sites and a precursor length of maximum 150 amino acid residues). Signal peptide was predicted using SignalP 4.1 (www.cbs.dtu.dk/services/SignalP, accessed on 6 August 2021), and propeptide cleavage sites were predicted at mono- and dibasic consensus sites. Multiple sequence alignments were performed by Clustal omega using Seaview software [134]. Gene organization was obtained by screening the new Refseq genome assembly (BioProject: PRJEB35351) (www.ncbi.nlm.nih.gov/assembly/GCF_902806645.1, accessed on 6 August 2021) [135] or Ensembl genomes database [136].

#### 3.5.4. Search for Differentially Expressed Transcripts during a Reproduction Cycle

Expression data corresponding to the contigs encoding *C. gigas* newly and formerly identified NPPs were retrieved. A hierarchical clustering (Euclidean distance) was applied using the Fastcluster package software [137]. This analysis assembled experimental samples together based on overall expression profile similarity. The transcript abundance variations were visualized by a heat map with a color scale, in which shades of red represented higher transcript expression and shades of green represented lower transcript expression. Expression levels of NPP encoding transcripts in the VG between different samples at different reproduction stages were compared using a one-way ANOVA followed by a Tukey post hoc test.

## 4. Conclusions

Through focused in silico mining of the transcriptome of the VG of the oyster *C. gigas*, the present study provides an overview of the NPPs/PPs expressed in this species, with the characterization of 44 new NPPs. Our results confirm that NPs hitherto presumed to be specific to ecdysozoan species—e.g., orthologs of ecdysis-regulated NPs or arthropod reproduction-regulated hormones—are present in *C. gigas,* confirming their early evolutionary origin. We also showed the structural proximity of lophotrochozoan QS-amide NPs with the protostomian PXXXamide NPs and identified novel, so far unknown, molluscan NPs. This study highlights the complexity of the repertoire of *C. gigas* NPs, which is probably useful to this sessile animal with limited behavioral responses for acclimating to environmental changes and supporting its high capacity for resilience. Moreover, among the NPPs differentially expressed over an annual reproductive cycle, some are known to mediate reproduction and associated processes in other species, but new candidates have also emerged. It will be of great interest to address the functionality of these NPs in reproduction. In an aquaculture context, better knowledge of regulators underlying reproduction may also prove valuable for the most important aquaculture shellfish worldwide.

## Data Availability

www.ncbi.nlm.nih.gov/sra/PRJNA662446 (accessed on 6 August 2021).

## Supplementary Materials

The following are available online at https://www.mdpi.com/article/10.3390/md19080452/s1, Supplementary Table S1: List and accession numbers of *C. gigas* NPP transcripts in, respectively the VG transcriptome (present paper: VG database: data retrieved from TSA (GIUV00000000), and in the transcriptomes of adult tissues and developmental stages (GigaTON database: http://gigaton.sigenae.org/, accessed on 6 August 2021 (Rivière et al., 2015) as well as in NCBI. NPP labelled in blue: NPP identified in previous studies, NPPs labelled in red: newly identified NPPs in this study. Supplementary Table S2: Identification by mass spectrometry of newly characterized mature neuropeptides. List of peptides molecularly characterized by nLC-fragmentation tandem MS analysis of oyster visceral ganglia extracts. The peptide sequence was validated according to a significance threshold of Mascot probability-based score >20 and checked manually to confirm or contradict the Mascot assignment. PTM: post-translational modification. PPM: expresses a mass fraction (1 ppm = 1 mg/kg). Supplementary Figure S1: Novel NPPs characterized in C. *gigas*. A: Alignment of *C. gigas* NPP sequences with the sequence of related precursors from other species. Amino acids with similar physicochemical properties are in the same color. Signature sequence features are indicated in red above the sequences. B Expression pattern of NPP transcripts during development (data retrieved from GigaTON, Rivière et al., 2015); Oo: Oocytes; Mor: Morula; Bla: Blastula; Gas: Gastrula; Tro: Trochophore larvae; D-sha: D-shaped larvae; Umb: Umbo larvae; Ped: Pediveliger larvae; Juv: Juvenile. C: Expression pattern of NPP transcripts in adult tissues (data retrieved from GigaTON, Rivière et al., 2015). MU: Adductor muscle; FGO: Female gonad (mix stages); MGO: Male gonad (mix stages); Gills; Hem: Hemolymph; LPA: Labial palps; Man: mantle; Dgl: Digestive gland. D Expression pattern of NPP transcripts in the visceral ganglia over a cycle of reproduction.
