# Supplementary material for: Transcriptome Profiling of the Pacific Oyster Crassostrea gigas Visceral Ganglia over a Reproduction Cycle Identifies Novel Regulatory Peptides
Source: Mar Drugs. 2021 Aug 7;19(8):452. doi: 10.3390/md19080452 (PMC8398477; doi:10.3390/md19080452)
Supplement: Supplementary file 1 [file marinedrugs-19-00452-s001.zip › Supplementary files/supplementary data1.pdf]

Supplementary material for:

Transcriptome profiling of the Pacific oyster Crassostrea gigas visceral ganglia over a reproduction cycle identifies novel regulatory peptides.

Emilie Realis-Doyelle1#, Julie Schwartz1, Cedric Cabau2, Lorane Le Franc1, Benoit Bernay3, Guillaume Rivière1, Christophe Klopp2, Pascal Favrel1*.
1 UMR BOREA, Normandie Université, UNICAEN, MNHN, CNRS-8067, IRD-207, Sorbonne Université, Esplanade de la Paix, 14032 CAEN cedex, France.
2 INRAE, UR875 Mathématiques et Informatique Appliquées de Toulouse, Plateforme SIGENAE, BP 52627, 31326Castanet-Tolosan Cedex, France.
3UNICAEN, SF 4206 ICORE, PROTEOGEN Core Facility, Esplanade de la Paix, 14032 CAEN cedex, France.
*Corresponding author: pascal.favrel@unicaen.fr

<u>Supplementary figure 1</u>:

Novel (neuro)peptide precursors characterized in *C. gigas*: Expression during development, in adult tissues and in the visceral ganglia over a cycle of reproduction.

A: Alignment of *C. gigas* NPP sequences with the sequence of related precursors from other species. Amino acids with similar physicochemical properties are in the same color. Signature sequence features are indicated in red above the sequences.
B Expression pattern of NPP transcripts during development (data retrieved from GigaTON, Rivière et al. 2015); Oo: Oocytes; Mor: Morula; Bla: Blastula; Gas: Gastrula; Tro: Trochophore larvae; D-sha: D-shaped larvae; Umb: Umbo larvae; Ped: Pediveliger larvae; Juv: Juvenile.
C: Expression pattern of NPP transcripts in adult tissues (data retrieved from GigaTON, Rivière et al. 2015). MU: Adductor muscle; FGO: Female gonad (mix stages); MGO: Male gonad (mix stages); Gills; Hem: Hemolymph; LPA: Labial palps; Man: mantle; Dgl: Digestive gland.
D Expression pattern of NPP transcripts in the visceral ganglia during the cycle of reproduction..

Bilaterian family

Cragi-IL17/CFSH (Interleukin 17 /Crustacean Female Sex Hormone)

A

```
                    1
C._gigas_1          ---------- -------MEK WILTLSIFFF LLVSTNCQHC ---------- ------SHRL SDDE------ ----------
C._gigas_2          ---------- -------MLR ICIL-VHMFI FLIPCDAMYI TS-------- -------SCK EISN------ ----------
P._yessoensis       ---------- ---------- ----MVVSVC VHTTFTCGTP -ARV---NLA IQLRSARNSI SP-------- ----------
L._gigantea         ---------- ---------- --MTMLVILC VLLGLVDSKS LEKV---LLN QKSRNADDCQ IPDN------ ----------
A._californica      MLPSLHIS-- -QTQVSLLLK LSIG-LLLYC ---ACR---- LTSA---GRP GSLGRAQQCR ESDE------ ----------
S._paramamosain_1b  MRSSTTFRSS LRCLIAVFLG ISVL------ -LLQEKSEAT LSV--PSYQQ PFLGS---GW -EETIPKHMK WSDEAIRIMK
S._paramamosain_2a  ---------- -------MLS LVLL------ -LNDSEEEAE ASRA---KSQ GL-KD----I STRAPPTFVK R---AKTCAN
C._maenas_1         MKQNGTSLRL TSFILFLFLE C--------- -------SVA FTPVYPSRVQ HFLQYLQQER QEDGLPL--- ----TTQSPK
L._vannamei_1a      MA--APRPQH LLCLS----- LSAL------ -VVAVFASPT FASARFSPRP PFLYH---ER -EKE-----E QPKPRAESPK
D._rerio            MSSALNLR-- -----FLMVA CMMG-LV--- ---------- LISF---GAE GASVRSDQKN KN-------- ----------
H._sapiens          ---------- --------MT LLPG-LLFLT WLHTCLAHHD PSLR---GHP HSHGTP-HCY SAEE------ ----------

                   81
C._gigas_1          ----FER--- -------QL- ---------- --------QH LHFEARGIEV LNNTF----- -SQPIDNSRC LNPYLDASPV
C._gigas_2          ----WKIKQQ LGYNPRE--- --S----DM- S-FHIIPQFK KMQRSSISDK SHEQFTR--- LFGNSTCPSE ----LNTSAF
P._yessoensis       ---------- ---------- ---------- E-SLLLTARE -----SDRHR SNVPTTIL-- EGETNVCPEN LNELDNTMYT
L._gigantea         ----ITE--- -------LIE SLTEIKRN-- K-SFLIPVQE SIYEVCDRRK SEIPF----- -VTPTNCPEN VTSRR---GG
A._californica      ---------- ---------- --------N- L-AAFVAQLN NI---SFRPS SGEPNTIRFQ HDLNSDCPS- --RWGAISND
S._paramamosain_1b  RKQEDLSTDE LQYFSEEQVD EASRVEYKVV PDPVIYTSQI IHKGVNCSSI K-------TD LHKNHITPEL --QLHPEWIH
S._paramamosain_2a  QNQSRCRRGQ VSMIPANQVK QGWEEDYTSV PDVLIQFSQK QAEETVCNDL S-------VQ LFRV-DLSEH --YLEPVWVK
C._maenas_1         RSSIIGHMNS IPYRTREQVM EGMMDTYVLV PRAIISSTSK LHHDVNCSEY REV-----NK IHGNRFEANF --NIRPTWLH
L._vannamei_1a      RSRDDFASDL VQFFSEEQVQ EATKIQYKSV PEPVIYTSQV LQQGVSCSTL H-------SE MHENYIKPEL --RLRPEWIY
D._rerio            ---------- ---------- ---------- ---------- ------SHPE ADHSYRL--- -VLDA--EF- --KASTNPIH
H._sapiens          ----LP---- LGQAPPHLLA RGAKWGQAL- P-VALVSSLE AA---SHRGR HERP------ -SATTQCPVL --RPEEVLEA

                  161
C._gigas_1          FLNERSLCPW DLVEDVNEN- --RYPQVLHF ARCRCSSCLD GMG--N---Y ECKPVSYRIP LLE-KRCVD- -------GVF
C._gigas_2          PVMFRSSCPW YIELSVDKT- --RIPVTMAK ARCRCKDCQT KTR--PHPDF SCAAVKTYRR VLREIPNQCD NE-----GFK
P._yessoensis       PPRHRSTCPY YMVSEHNPN- --RFPSILTS VRCRCQQCLE AGG--RSLEN VCEPVCVTEV VLMQEECDN- -------GVF
L._gigantea         KLNQRSVCPW DMVQEYLPG- --YFPSVINV ARCR--LCER CSG--VNSTT SCQPIVYRIP VLKLTGCVS- -------GFY
A._californica      GLHNISLCPW TYHINTDYN- --RYPNDILY ARCSCGWCSS QLS--VGPRS RCYPLYHSYQ VIRRE-CDR- -------GQY
S._paramamosain_1b  TSQLIGTCPT HYVTRELPPM --YSPSVVVE AVCTCTGSKC SR---DGH-- QCLPVSRHIP VWVRQ----- -------GPN
S._paramamosain_2a  EIVHLGMCPS KLQTRSFGKD --VWPSTIVE AKCLCNNQPC SN---LGGDF RCQAVRKPIR TWVRH----- -------VEK
C._maenas_1         RSKTISTCPT QYVERQIQGP FPIQPVTILE AKCVCEGSQC SQ---DGS-- ICVAVKYRLP VWIRV----- -------DSD
L._vannamei_1a      ESKLIGDCPT HYVTRELPSI --YSPSTVLE AVCACGGSQC SE---DGH-- QCVPVSRHVP VWVRR----- -------GPN
D._rerio            PINNDSISPW TYMFTHNES- --LYPTSIAE AKCSLTGCLI DGV--EVQDY ESKPIYTQIM VLRRIRGEK- -------PNY
H._sapiens          DTHQRSISPW RYRVDTDED- --RYPQKLAF AECLCRGCID ARTGRETAAL NSVRLLQSLL VLRRRPCSRD GSGLPTPGAF

                  241
C._gigas_1          QYVKTYLDLP VGCTCTKPRV IQVSKRIQNL HIKRMNFQTL RQKIKGTY
C._gigas_2          MYTSDVEEVP VACTCVATRT RI-------- ---------- --------
P._yessoensis       QYNPTEKQIQ VACTCARRRE EESSSDNEGT TPI------- --------
L._gigantea         TYEKKYKKRA VGCTCARRKT MRARRRNRV- ---------- --------
A._californica      RYTYTHESLP IACVCSHHAA SVVDIE---- ---------- --------
S._paramamosain_1b  FHVLDVEELT VACACVRRPS VGGNFIFASA VHSK------ --------
S._paramamosain_2a  FMP-VQEMVT VGCVCVQRTS PEGKYARPAI EA-------- --------
C._maenas_1         GYTTDTVELT VACACAKNPS RDGGYVDLSE N--------- --------
L._vannamei_1a      LHVLDVEELT VACACARRPS AGGNFIFSAA VE-------- --------
D._rerio            SFKLEYKTIA VGCTCVRPYV EQL------- ---------- --------
H._sapiens          AFHTEFIHVP VGCTCVLPRS V--------- ---------- --------
```

D

Cragi-IL17/CFSH1

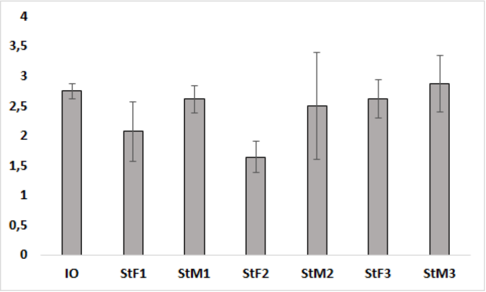

Cragi-IL17/CFSH2

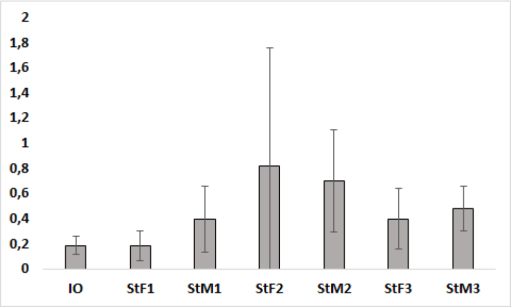

A: Alignment of *Cragi-IL17/CFSH1*(CGG_contig_09983 genome scaffold 678 232727 to 233272 (+)), *Cragi-IL17/CFSH2* (CGG_contig_07061 genome scaffold160 103046 to 103573 (+), *P. yessoensis* (XP_021370734.1), *L. gigantea* (XP_009051410.1), *A. californica*(XP_005097238.1), *S. paramamosain_1b* (Veenstra, 2016), *S. paramamosain_2a* (Veenstra, 2016) , *C. maenas_1* (Veenstra, 2016) , *L. vannamei_1a* (Veenstra, 2016) *CFSH*, *D. rerio* (NP_001018623.1), *H. sapiens* (AAH69152.1) IL17 sequences.

D: Expression pattern of *Cragi-IL17/CFSH1*(CGG_contig_09983) and *Cragi-IL17/CFSH2* (CGG_contig_07061) encoding genes

Cragi-EH (Eclosion Hormone)

A

```
                     1
C._gigas_EH1         ---------- ---------- ---------- ---------- ---------- ----------
C._gigas_EH2         ---------- ---------- ---------- ---------- ---------- ----------
M._sexta             MAGKVTV-AF FMFAM----- ---------- ---------- ---------- ------IAFL
D._melanogaster      MNCKPLILCT FVAVA----M C--------- ---------- ---------- -LVHFGNALP
B._mori              MANKLTA-VI VVALA----- ---------- ---------- ---------- ------VAFM
O._victoriae         -------MKT VMVSCLVLLL IGESFGAALL DIDEVDDT-- ---------- -----NAAFA
D._melanogaster_ITP  MCSRNIKISV VLFLVLIP-I F--------- ---------- ---------A ALPH--NHNL
M._rosenbergii_CHH   MI-RSSVMGP TMFLVVLL-L IA-THQTSAW SVDGLARIEK LLSTSSSASA ASPTRGQALN

                    61
C._gigas_EH1         ---------- ---------- LLPKCLQKCG ECMETVNNYD -TRSCLSYCL DGKTDD---Q
C._gigas_EH2         ---------S LECKK----- -------FCF QCKLTYPQYN -GNKCLEECE MSKGFKLDVN
M._sexta             AN-FG--YVE CNPAIATGYD PMEICIENCA QCKKMLGAWF EGPLCAESCI KFKGKLIP-E
D._melanogaster      AI-SH--YTH KRFDSMGGID FVQVCLNNCV QCKTMLGDYF QGQTCALSCL KFKGKAIP-D
B._mori              VN-LD--YAN CSPAIASSYD AMEICIENCA QCKKMFGPWF EGSLCAESCI KARGKDIP-E
O._victoriae         LNRLVQRRSD GNDVLMERER RRKSCLVECV TCSRYTL--L PTTDCYEGCN KPTKSKLA--
D._melanogaster_ITP  SKRSN--FFD LECKGIFNKT MFFRLDRICE DCYQLFRETS IHRLCKKDCF DSKWFG---E
M._rosenbergii_CHH   LKKRA--ILD QSCKGIFDRE LFKKLDRVCD DCYNLYRKPY VAIDCREGCY QNLVFR---Q

                   121
C._gigas_EH1         CNLFSV-SAA ---G------ -FGKR----- -----
C._gigas_EH2         CINYW----- ---------- ---------- -----
M._sexta             CEDFASI--- --APFLNKL- ---------- -----
D._melanogaster      CEDIASI--- --APFLNALE ---------- -----
B._mori              CESFASI--- --SPFLNKL- ---------- -----
O._victoriae         -LNTWT---- ------ACKG MLGGQ----- -----
D._melanogaster_ITP  CLKVLLIPEE EISNLQHFLR VVNGSPISFN MGPQT
M._rosenbergii_CHH   CIQDLQLMDQ -LDEYANAVQ IVGK------ -----
```

B C D

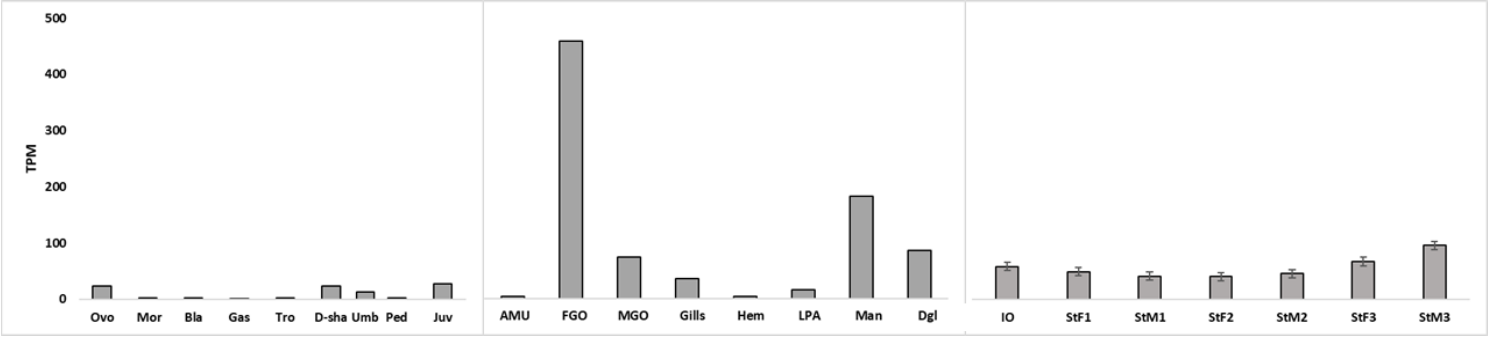

A : Alignment of *C. gigas* (CGG_contig_11351), *M.sexta*(AAA29310.1), *D.melanogaster* (AAF55423.1), *B.mori* (NP_001037307.1), *O.victoriae*(ASK86246.1) EH, *D. melanogaster ITP* (NP_611931.3) and *M. rosenbergii* (AAF29534.1) CHH sequences.

B : Expression pattern of *Cragi-EH* (CHOYP_contig_053689) encoding gene during development

C : Expression pattern in adult tissues.

D: Expression pattern of *Cragi-EH* (CGG_contig_11351) encoding gene in the visceral ganglia during the cycle of

A

Cragi-Nesfatin

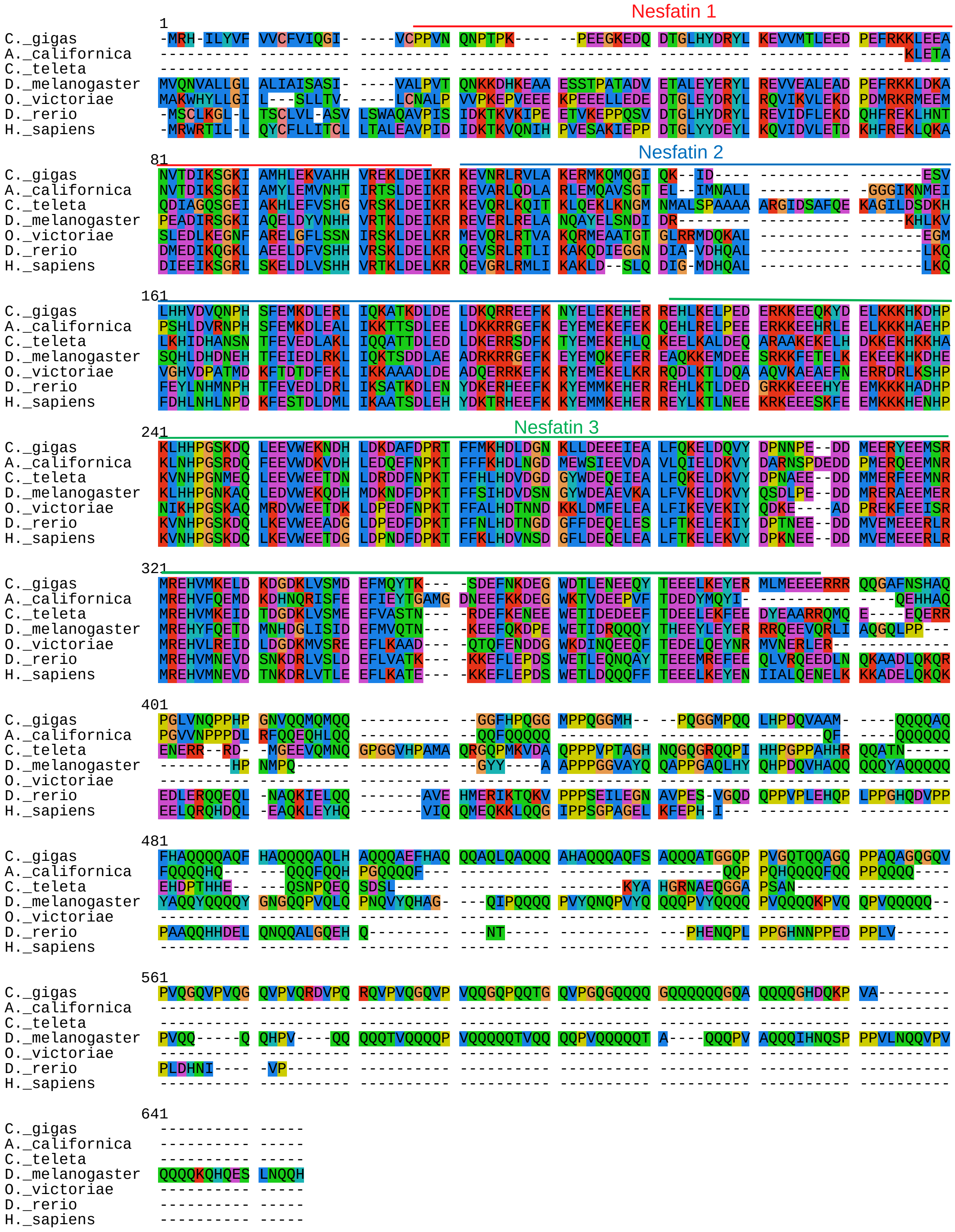

B C D

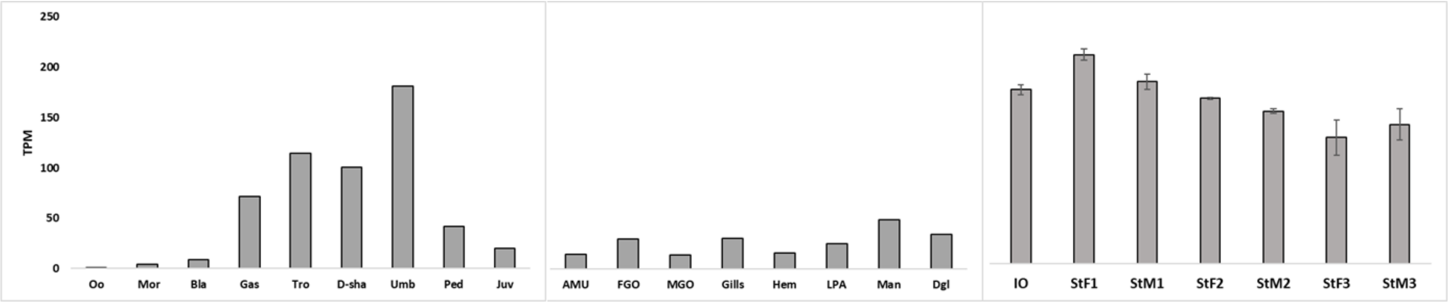

A : Alignment of *C. gigas* (CGG_contig_14701), *A. californica* (XP_012940282.1), *C. teleta* (ELT97554.1) , *D. melanogaster (*AF044203). ; *O. victoriae* (ASK86275.1); *D. rerio* (NP_958901.1) and *H. sapiens sp* (P80303.3) nesfatin sequences.
B : Expression pattern of Cragi-Nesfatin encoding gene (CHOYP-NUCB1.1.1) during development.
C : Expression pattern in adult tissues.
D : Expression pattern of Cragi-Nesfatin (CGG-contig-14701) encoding gene in the visceral ganglia during the cycle of reproduction.

Cragi-Neuroparsin/IGFBP

A

```
               1
C._gigas       --------MK GIV--FVSCF LGFLCLSMGQ ----EDIPSD CGAC---DRS ACPEQKDKTC KAGITRDRCK
P._yessoensis  ---------- -MG--VSMKY FGVLCLLCAA ----VWAEEE CGEC---NRN ECPALDTSKC LANYKRDHCN
L._gigantea    ---------M RIL--FVLGA WA--LLGFVS ----ASPNTR CNDC---KKE LCPKLSRSEC LTGIVKDGCD
S._gregaria    -----MKPAA A----LAAAT LLIAVILFHR ----AEANPI SRSC---EGA NC-VVDLTRC EYGEVTDFFG
D._magna       MHQKNTHPFK SAI--WPFFI SAFFIVFFSR ----VNCAPR CTAC---ADK KD-CLPPKNC IYGVVKDYCG
S._salar       -----MLGLY KKLTLLAAMS LSLLTSLAQS SPVVGPEPIR CAPCTQEKLD ECPAISP-DC KQVLREPGCG
H._sapiens     ---------M SEVPV--ARV WLVLLLLTVQ VGVTAGAPWQ CAPCSAEKLA LCPPVSA-SC SEVTRSAGCG

               71
C._gigas       CCQVCAQAEG EICDISGASN KYPPCGDYLT CKMDQD---- -----RNIGI CVCDYQDTLC GTD-------
P._yessoensis  CCLVCMQHEL QPCDLPNRPH QYGECGDYLT CSP-VD---- -----DNVGQ CTCVNQEVIC GSD-------
L._gigantea    CCDVCARMES QTCDLPETPY VNGKCGDGLS CEET------ -----EFGQV CVCEHDQIIC GSN-------
S._gregaria    -RKVCAKGPG DKCGGP--YE LHGKCGDGMD CRCGVC---- -----SG--- --CSMQSLEC FFF-------
D._magna       -RDICAKGPG HRCGGK--WN SLGTCGEGLF CSCNRC---- -----GG--- --CSLITLEC FNL-------
S._salar       CCLACALEKG ASCGVY---- -TAHCAQGLK CSPRPGDPRP LHSLTRGQAI CTEDQGQEEV E----GVPDH
H._sapiens     CCPMCALPLG AACGVA---- -TARCARGLS CRALPGEQQP LHALTRGQGA CVQESDASAP HAAEAGSPES

               141
C._gigas       --------DV TYTSICQMRE AQ-------- -------NTK --GRDLKVKN SGPCKT---- -APRILTGPD
P._yessoensis  --------GL TYDNLCQLYQ EN-------- -------GNR --DEVVIVKS FGKCKTETSA SKPVITSGPQ
L._gigantea    --------NV TYNNMCGLMA DM-------- -------RRA GLKSELEVKF VGPCDA---- -GAKIVSSPM
S._gregaria    --------EG AAPNSC---- ---------- ---------- ---------- ---------- ----------
D._magna       --------TC I--------- ---------- ---------- ---------- ---------- ----------
S._salar       SS-------- -LTYFLGLNT PFDTKNEGAQ ESI----KAK VNTIRKKLVE QGPCHIELHA ALDKITSSQQ
H._sapiens     PESTEITEEE LLDNF-HLMA PSEEDHSILW DAISTYDGSK ALHVTNIKKW KEPCRIELYR VVESLAKAQE

               211
C._gigas       SVTNV----- ------TGDK VVLMCEATGF PIPHIGWLFQ RTDDETNSLP GDDMAISVVT RGGPEKYQVT
P._yessoensis  DIVNG----- ------TGSD VAMLCEATGN PLPYILWLFT RADGTTINMP GADKDVSMNL RGGPEENQVT
L._gigantea    YTKNS----- ------TGGT VILSCEAVGN PTPHIAWLYT RADGETFSVP GDDEFTLTAA RGGPGRYQIT
S._gregaria    ---------- ---------- ---------- ---------- ---------- ---------- ----------
D._magna       ---------- ---------- ---------- ---------- ---------- ---------- ----------
S._salar       ELGEKFTNFY LPNCDKHGFY KAKQCESSLV GPPARCWCVF SWNG------ -KKIPGSNYL LGGLECQLE-
H._sapiens     TSGEEISKFY LPNCNKNGFY HSRQCETSMD GEAGLCWCVY PWNG------ -KRIPGSPEI RGDPNCQMY-

               281
C._gigas       GWLQVDEASK HHEGYYICVA HNKHGSDKQT GRIKIHEKKR VAPSGRGQQA PRGVLHLCGP QQARLRQTDG
P._yessoensis  GWLQVMNIAK KHEGDYTCIA KNTEGDVNDT GRLKVVETID IAEETGNEA- ---------- ----------
L._gigantea    GWVQIEGLRK THEGDYTCIV QNKYKKDTAK ARVKVVPRK- ---------- ---------- ----------
S._gregaria    ---------- ---------- ---------- ---------- ---------- ---------- ----------
D._magna       ---------- ---------- ---------- ---------- ---------- ---------- ----------
S._salar       --L------- ---------- ---------- ---------- ---------- ---------- ----------
H._sapiens     --FNVQN--- ---------- ---------- ---------- ---------- ---------- ----------

               351
C._gigas       EDKDPREKVK PRRAKRRWRP VISLLAKRLS LEYFLALCKL LS
P._yessoensis  ---------- ---------- ---------- ---------- --
L._gigantea    ---------- ---------- ---------- ---------- --
S._gregaria    ---------- ---------- ---------- ---------- --
D._magna       ---------- ---------- ---------- ---------- --
S._salar       ---------- ---------- ---------- ---------- --
H._sapiens     ---------- ---------- ---------- ---------- --
```

B C D

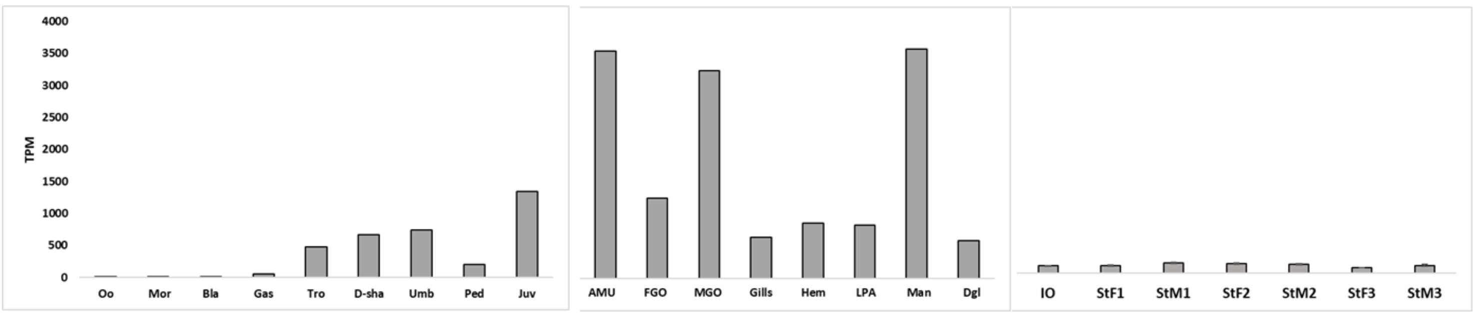

A : Alignment of *C. gigas* (CGG_contig_00821, EKC32441, XM_011444842.3), *P. yessoensis* (XP_021371917.1), *L.* gigantea (XP_009066688.1), *S. gregaria* (CAC38869.1), *D. magna* (XP_032796889.1) neuroparsins, *S. salar* (NP_001117096.1) and *H. sapiens* (AAA52784.1) IGFBP sequences .
B : Expression pattern of Cragi-Neuroparsin encoding gene (CHOTP-LOC769726.1.1) during development.
C : Expression pattern in adult tissues.
D : Expression pattern of Cragi-Neuroparsin (CGG-contig-00821) encoding gene in the visceral ganglia during the cycle of reproduction.

Cragi-Urotensin

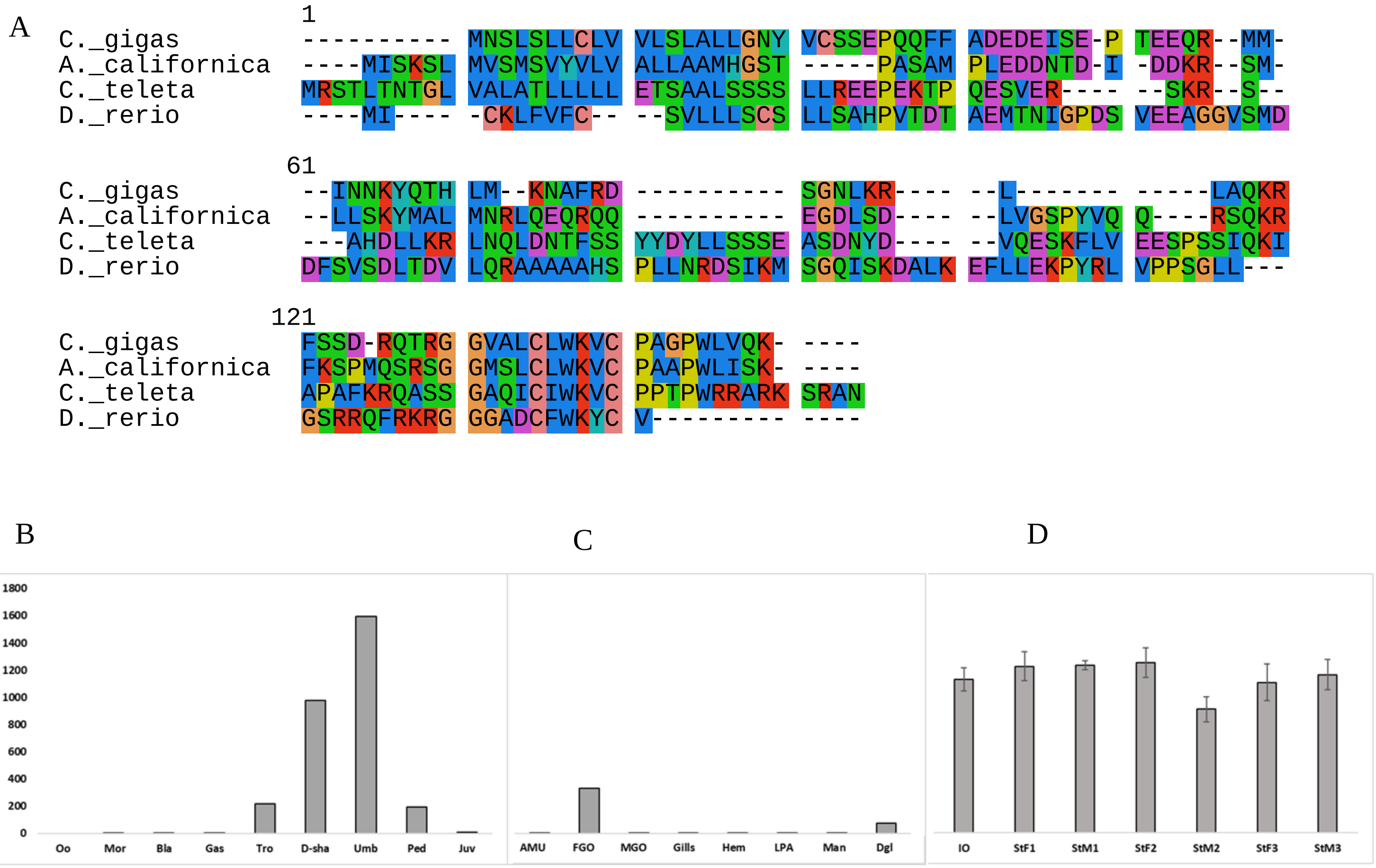

A : Alignment of *C. gigas* (CGG_contig_30764, CGG_contig_09857, CGG_contig_22724, CGG_LOC105343318), *A. californica* (XP_012945633.1), *C. teleta* (ELU12290.1) and *D. rerio* (XP_005157985.1) urotensin II sequences.
B : Expression pattern of Cragi-Urotensin encoding gene (CHOYP-contig-004921) during development.
C : Expression pattern in adult tissues.
D : Expression pattern of Cragi-Urotensin (CGG-contig-30764) encoding gene in the visceral ganglia during the cycle of reproduction.

A

Cragi-Prokineticin

```
               1
C._gigas_1     MTLTRALWIL LVCYHGA-VL ---------- ---------- ---------- ---------- ----YTDAAA
C._gigas_2     -------MKL FLCFAVFACS ---------- ---------- ---------- ---------- -QAFFLDSLF
C._gigas_3     ---------- MHCLTGLAFC ---------- ---------- ---------- ---------- -ITLGIVMGA
C._gigas_4     MA-PINPSIL AICFGLCSTF ---------- ---------- ---------- ---------- ----VIKDTV
C._gigas_5     ---------- ---------- ---------- ---------- ---------- ---------- -------MEN
P._dumerilii   -----MANIF HVCLLLMVFA TIQGIPSKRG RSRPLFVGKR YPPYMPYRRT DYHGKRGRLQ LLFVGKRQPT
A._mellifera   ----MMTSIF EISFLLFVLA Y--------- ---------- ---------- ---------- ----PCHAQN
P._vannamei    ---------M AVSSAVRMLA AAC------- ---------- ---------- ---------- -LVVSAVGMR
D._rerio       -----MTS-- NISFFLCLL- ---------- ---------- ---------- ---------- -LVSRGWTAV

              71
C._gigas_1     TNKGCNSATE CLDNECCVSI YQPLGRRQLH GHYSGHCVPM GMDGDGCLVK TRNATT---- ---------R
C._gigas_2     GSGGCKSDDD CSSGGCCMTD VF-------- GDKSCVH-QY LHYLQECRMD GHDHH----- ----------
C._gigas_3     LRNECSSDPD CPSTHCCVTD YH-------- GKYC--S-AY QNVSQPCHLP GHSLH----- ----------
C._gigas_4     EPRMCNLATD CHDNECCVSN VRPIGKRQT- -SFHGHCIPL GKVGDGCLVR NGNATS---- ---------R
C._gigas_5     KCSMAARDTS CPTGYCCAYD EF-------- FRIIYYCKKL GAINESCSTV ---AS----- ----------
P._dumerilii   YEMSCRTDLD CPSSHCCLAD LE-------- -NSSGTCTPL RHMEQICTTS S----F---- ---------Y
A._mellifera   DYIKCQTSSE CQPNHCCTLG SV-------- RYSIPQCKPM QGKGEVCRPT NSSTTFNVTL GYPDGSSLKI
P._vannamei    RLGDCSSSAD CGPGACCTVG FN-------- RYSVPQCTPL GDLGDWCRIM NPPRE--LNL AYPNGLQIVL
D._rerio       ITGACEKDSQ CGGGMCCAVS LW-------- IRSLRMCIPM GQEGEDCHPM SHKVPF---- ---------F

             141
C._gigas_1     PLDVVLSCPC TPGLYCHGDH LYVVPLG--- ---------- -EQGHCTSP- ---------- ----------
C._gigas_2     ----S--CGC GSGLYCESYA NVHGAAVE-- ----TQHMGS TMYGLCEHKV NA---TSYYN FIVFNK----
C._gigas_3     ----TYHCNC APGYSCQPMH LTADASLAQA LHQVEEILTH TGYGTCQIVV ---------- ----------
C._gigas_4     PTELVFACPC TPGLYCHGDH HYDIPLG--- ---------- -EIGHCANP- ---------- ----------
C._gigas_5     ----DADCPC EPGLTCVPTV QGSS--F--- ---------V TIYGRCKPVG MT---TEEAG TTEAEHTNHG
P._dumerilii   DGISYPMCPC ASGLSCAISP -----EK--- ---------- -RFGICTSSW DEEEGEEQYN Y---------
A._mellifera   EDVYFIFCPC IDGLSCEKGI ---------- ---------- -----CKEKN ---------- ----------
P._vannamei    TDTYHGMCPC RPELVCSRAS -----S---- ---------- ----TCQLPN ESTLDQEDNS LYRD------
D._rerio       GKRLHHTCPC LPNLACITTA -----DG--- ---------- --KSKCLPPF ----PFQDQY L---------

             211
C._gigas_1     -------
C._gigas_2     -------
C._gigas_3     -------
C._gigas_4     -------
C._gigas_5     HHVTKHG
P._dumerilii   -------
A._mellifera   --------
P._vannamei    --------
D._rerio       --------
```

B

C

D

Cragi-Prokineticin 1

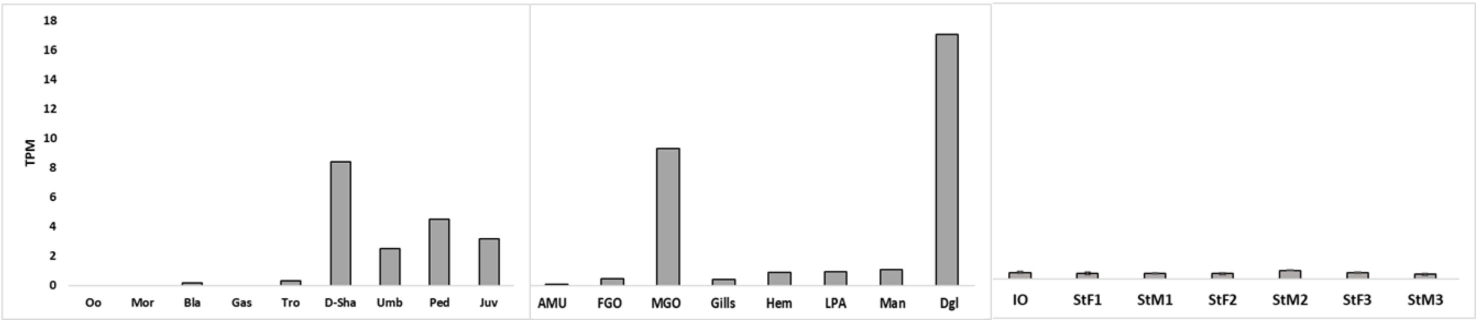

Cragi-Prokineticin 2

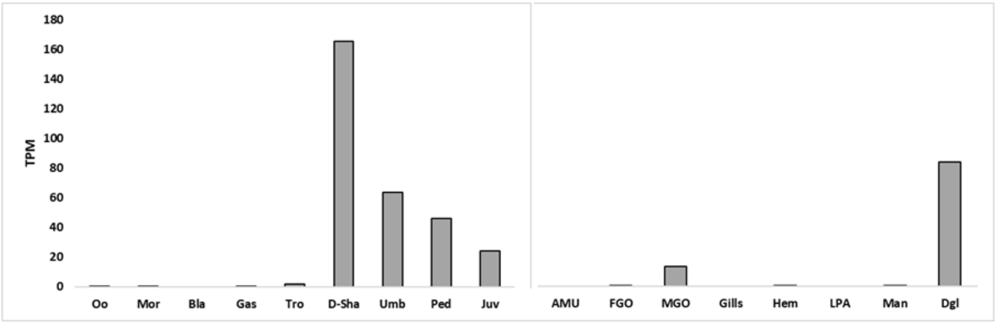

B C D

Cragi-Prokineticin 3

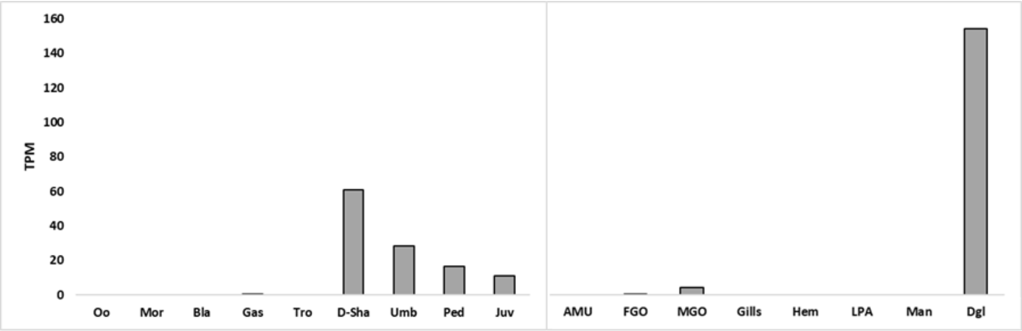

Cragi-Prokineticin 4

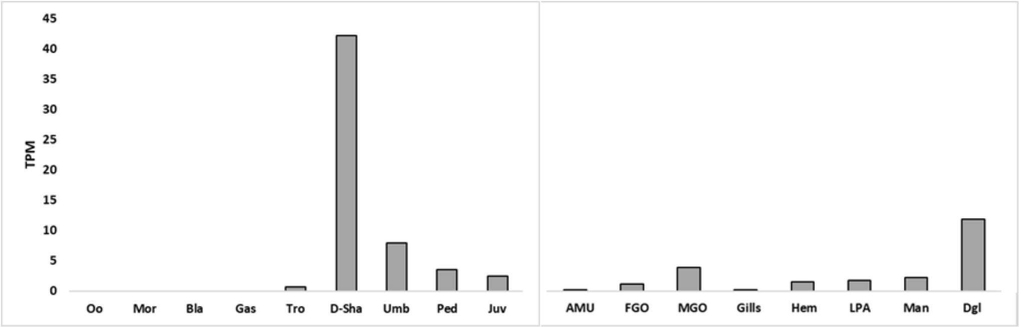

Cragi-Prokineticin 5

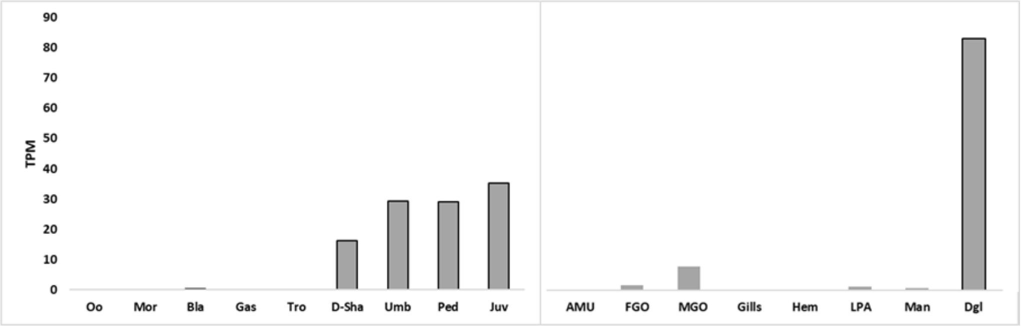

A : Alignment of *C. gigas 1* (CGG_contig_16708), *C. gigas 2* (CHOYP_contig_004850*), *C. gigas 3* (CHOYP_contig_003956*), *C. gigas 4* (CHOYP_contig_004051*), *C. gigas 5* (CHOYP_BM8F.2.2, EKC19072.1 P. yessoensis (XP_021370383.1), *P. dumerilii* (Conzelmann et al. 2013), *A. mellifera* (XP_003250271.1), *P. vannamei* (ADM53424.1) astakine and *D. rerio* (ACV04795.1) Prokinecitin sequences. * Data retrieved from GigaTON (Rivière et al, 2015)
B : Expression pattern of Cragi-Prokineticin 1 (CHOYP_contig_002587); Cragi-Prokineticin 2 (CHOYP_contig_014127); Cragi-Prokineticin 3 (CHOYP_contig_003956); Cragi-Prokineticin 4 (CHOYP_contig_004051) and Cragi-Prokineticin 5 (CHOYP_BM8F.2.2) encoding genes during development.
C : Expression pattern in adult tissues.
D : Expression pattern of Cragi-Prokineticin1 (CGG_contig_16708) encoding gene in the visceral ganglia during the cycle of

Cragi-Trunk/PTTH

A

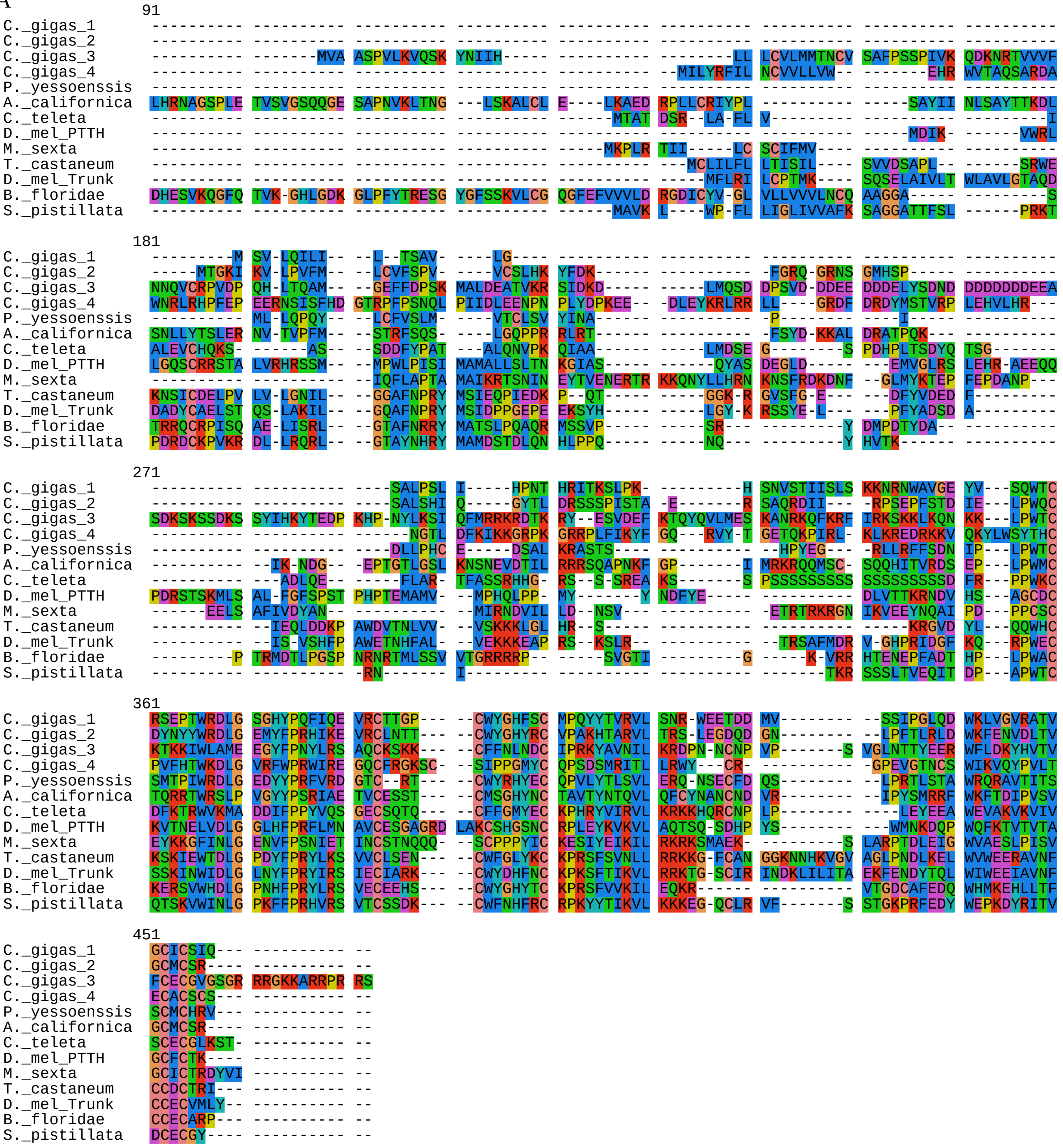

Cragi-PTTH 1

B

C

D

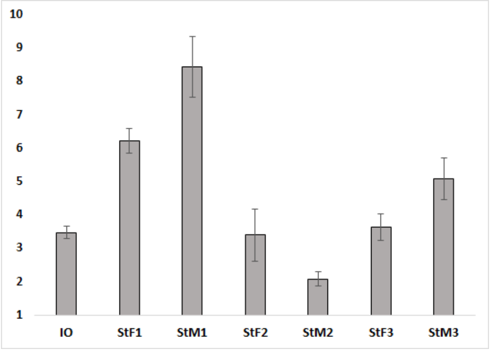

B

C

D

Cragi-PTTH 2

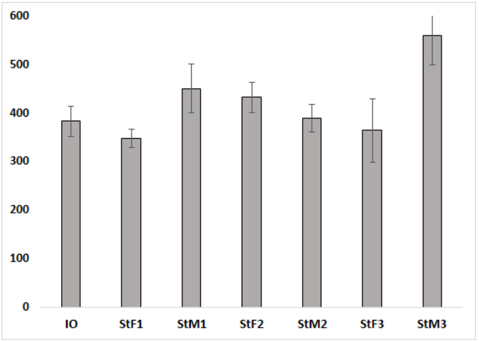

Cragi-PTTH 3

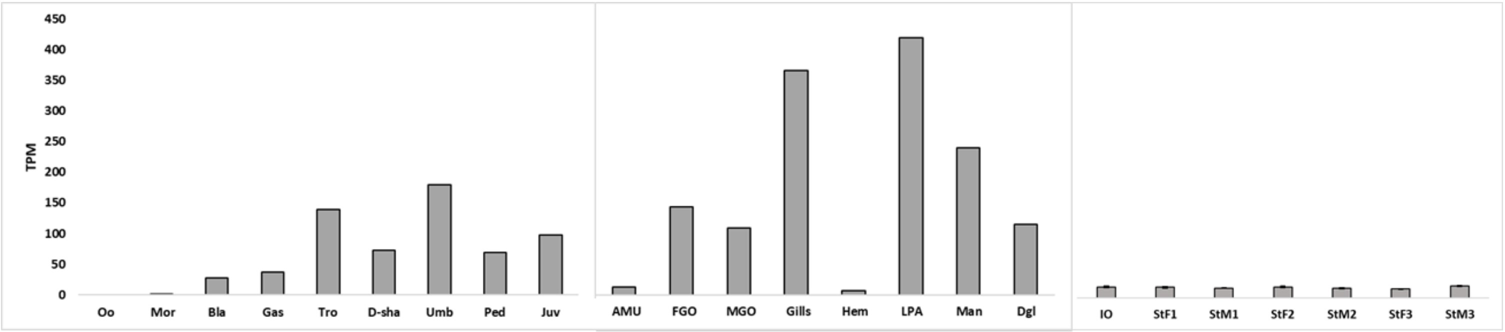

Cragi-PTTH 4

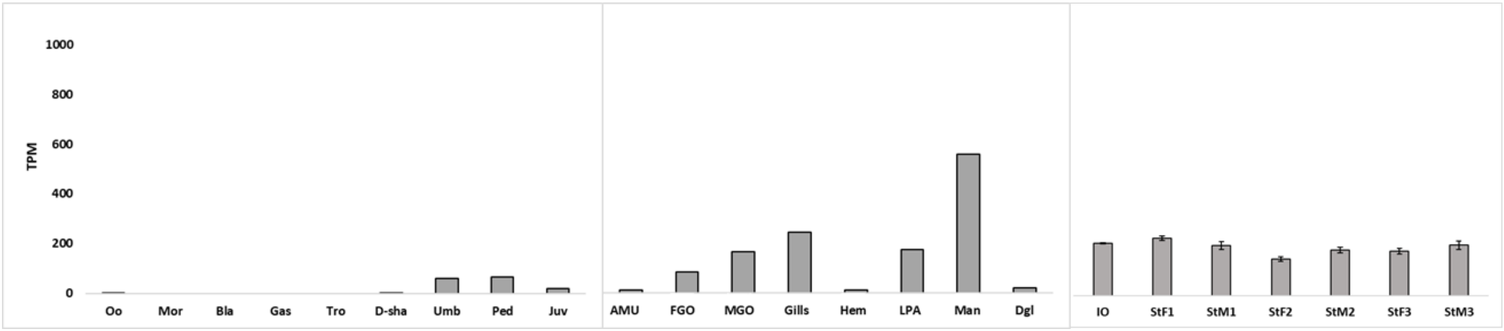

A : Alignment of *C. gigas* PTTH1 (CGG_contig_11546, genome Scaffold257 [810982 to 811595 + 810776 to 810900 + 810895 to 810992 + 814723 to 814836), *C. gigas* PTTH2 (CGG_contig_28778; EKC39796; XP_011450369.1), *C. gigas* PTTH3 (CGG_contig_24835, genome Scaffold43954 [239163 to 239624 + 238782 to 239042]) , *C. gigas* PTTH4 (CGG_contig_10820 EKC21705, XP_011448209.1), *P. yessoensis* (XP_021368706.1), *A. californica* (XP_012942268.1), *C. teleta* (ELU11356.1), *D. mel* (NP_001285550.1), *M. sexta* (AAG14368.1) , *T. castraneum* (XP_971946.1) PTTH sequences, *D. mel* (AAF52896.1) , *B. floridae* (XP_002586716.1) and *S. pistillata* (PFX31008.1) trunk sequences .
B : Expression pattern of Cragi-PTTH 3(CHOYP_TRK.1.1), PTTH 4 (CHOYP_BRAFLDRAFT_113863.1.1) encoding genes during development.
C : Expression pattern in adult tissues.
D : Expression pattern of Cragi-PTTH 1(CGG_contig_11546), PTTH 2 (CGG_contig_28778), PTTH 3 (CGG_contig_24835), PTTH 4 (CGG_contig_10820) encoding genes in the visceral ganglia during the cycle of reproduction.

Protostome families

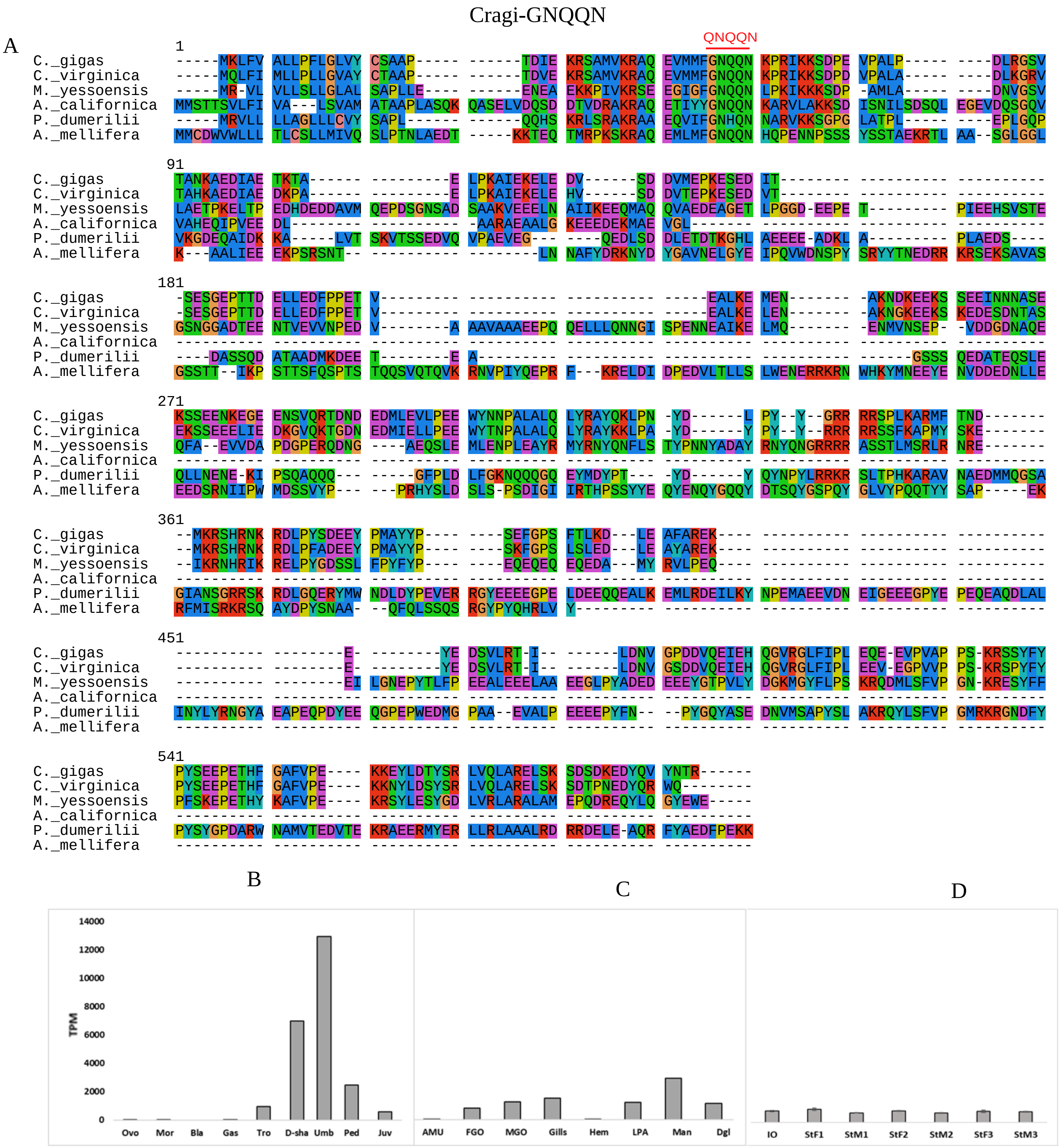

A : Alignment of *C. gigas* (CGG_contig_21126), *C. virginica* (XP_022294073.1), *P. yessoensis* (AXN93526.1 GNQQNxP), *A. californica* (XP_012937567.1), *P. dumerilii* (AHB62367.1) and *A. mellifera* (NP_001161308) GNQQN sequences.

B : Expression pattern of Cragi-GNQQN encoding genes (CHOYP-contig-035767) during development.

C : Expression pattern in adult tissues.

D : Expression pattern of Cragi-GNQQN encoding gene (CGG_contig_21126) in the visceral ganglia during the cycle of reproduction.

Cragi-Pleurin

A

```
               1
C._gigas       --MQSLFWAL IVLVSCDCAL CLFYTSSKEN DYPRFGKRNS RIHSDAWM-- ---DKDSIED
P._yessoensis  MSPHSILTLL TTFAIIQLAK AIFYTNKEGN DFPRIGKRRH LYQSEAWS-- ---RDSVPSD
A._californica MQSHVSSSLL ALCILTGSAC AMFYTKGSDS DYPRIGRRSF YTTGNGNHYP RIGRRDSPGA

              61
C._gigas       --YN------ --------EI IDSSEIKPTQ YL-SQSRL-- ---------- ----------
P._yessoensis  --GL------ --------PG ETDRQMEPMY NQ-DEGEIPI TS-------- ----------
A._californica AAGTDILYPS AASGFSDFPA SASAAKRGIF TQSAYGSYPR VGRRSQAGDD GSQEGLQTAR

             121
C._gigas       ---------- ---------- -------RMF KRGTNLLFQF LDRNGDQLIT KNEFTANSFT
P._yessoensis  ---------- ---------- -RREHQNPMS SYSKAMYYRA LGLNGYRAFS KNKVQASPNN
A._californica SKIFQKITGL DGPVEEELGD GSAERLEADN QIGLELMFLA FDLDGDQALS KSEFKSGMEK

             181
C._gigas       EILDALTKDH HGK
P._yessoensis  GIRGTRTYY- ---
A._californica FRRHNPLC-- ---
```

D

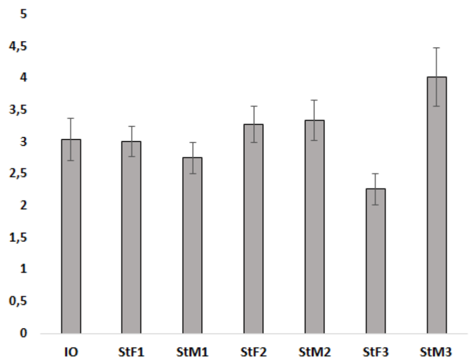

A : Alignment of *C. gigas* (CGG_contig_22966), P. *yessoensis* (AXN93514.1) and *A. californica* (NP_001191504.1) pleurin sequences.

D: Expression pattern of Cragi-Pleurin encoding gene (CGG_contig_22966) encoding gene in the visceral ganglia during the cycle of reproduction.

Cragi-Prohormone 3

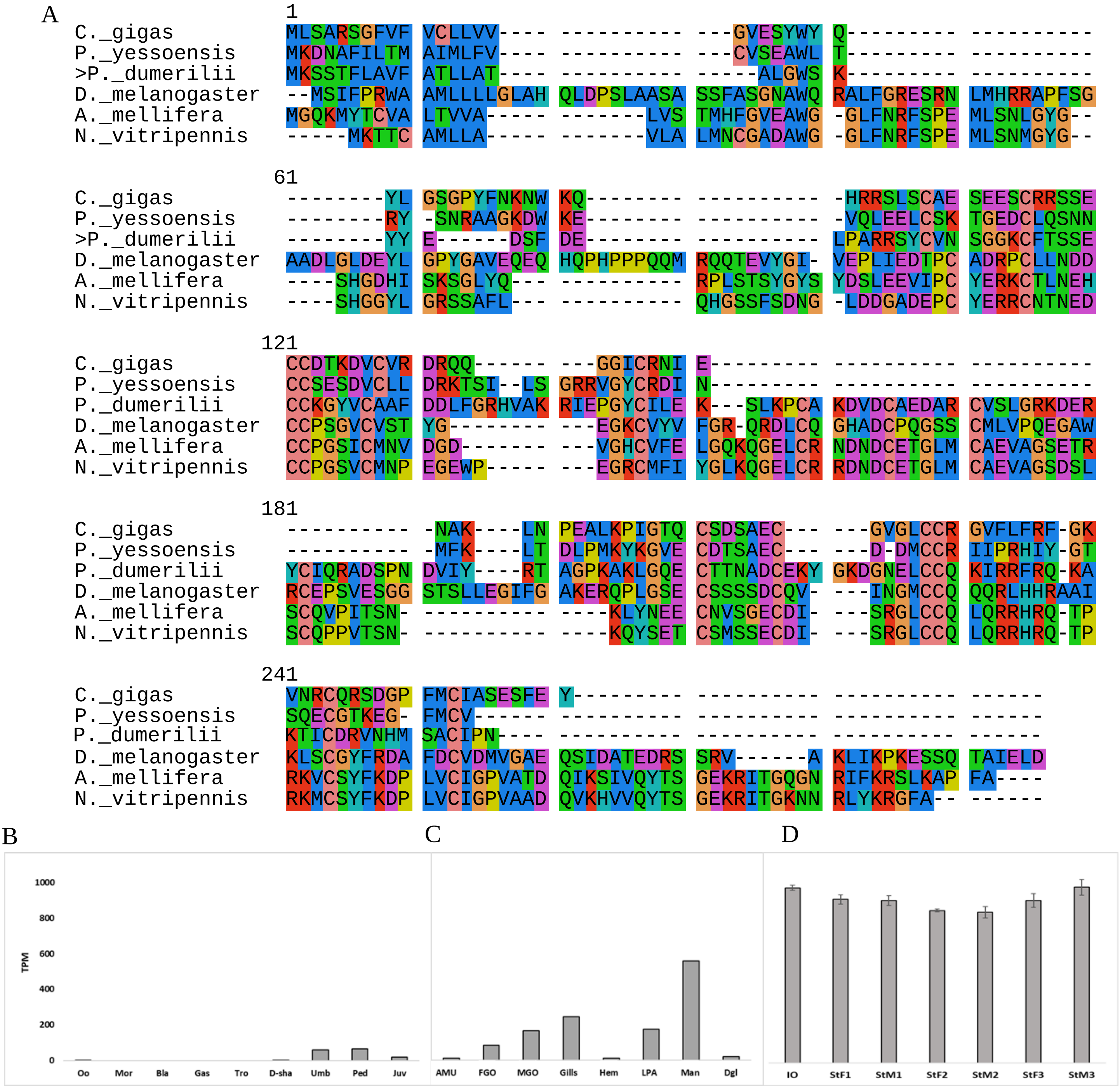

A : Alignment of *C. gigas* (CGG_contig_31012, XP_011427435.1) and *P. yessoensis* (XP_021368853.1), *P. dumerilii* (AHB62385.1) , *D. melanogaster* (ABO52824.1), *A. mellifera* (XP_001122204.2) and *N. vitripennis* (XP_001604833.1) prohormone 3 sequences.
B : Expression pattern of Cragi-Prohormone 3 encoding gene (CHOYP-contig-038859) during development.
C : Expression pattern in adult tissues.
D : Expression pattern of Cragi-Prohormone 3 encoding gene (CGG_contig_31012) in the visceral ganglia during the cycle of reproduction.

Cragi-Prohormone 4

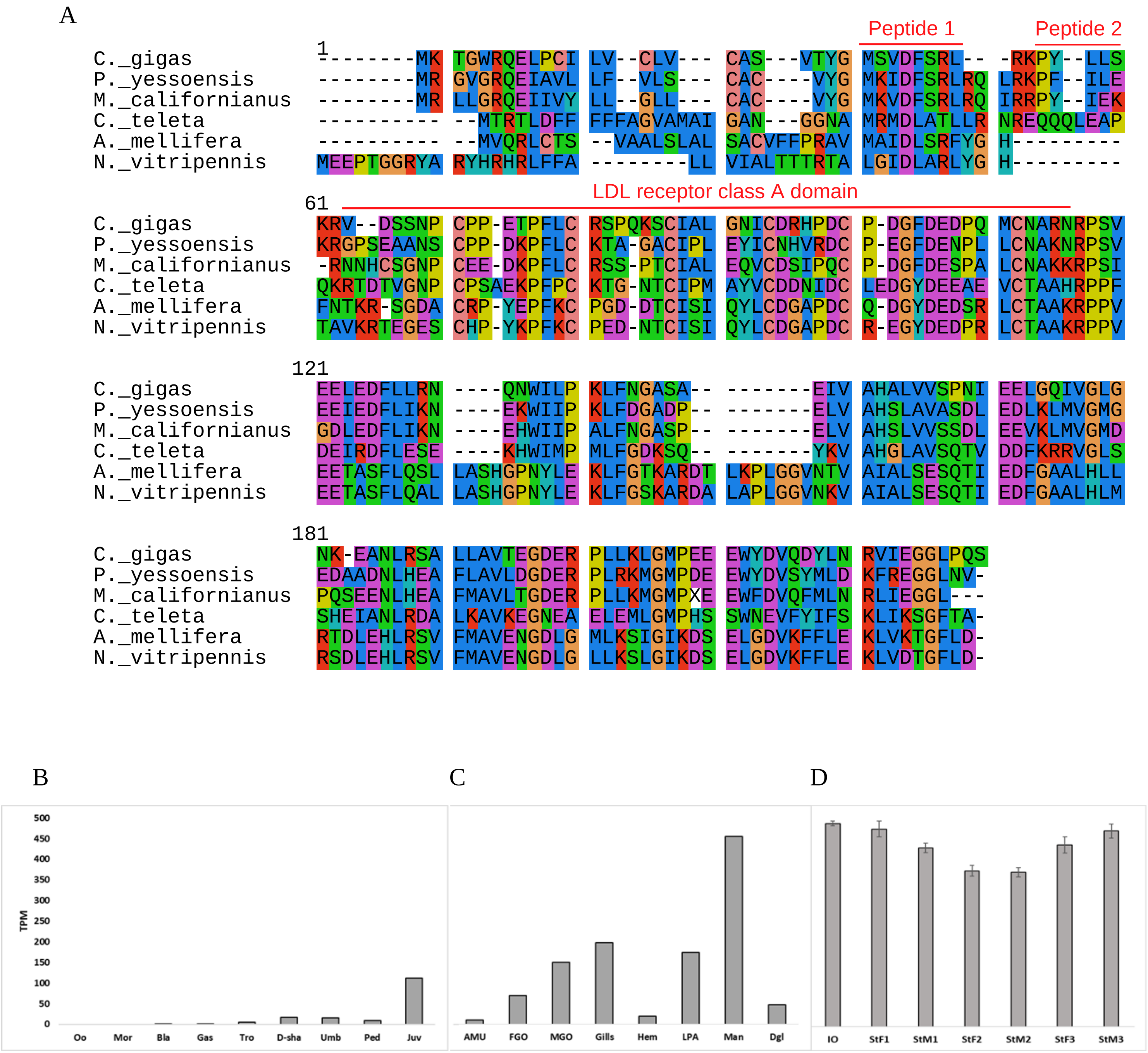

A : Alignment of *C. gigas* (CGG_contig_28466, CGG_contig_14367, CU997943.1), *P. yessoensis* (XP_021370383.1), *M. californianus* (GE760547) , *C. teleta* (EY614440), *A. mellifera* (XP_392764.1) and *N. vitripennis* (XP_001603909) prohormone 4 sequences.
B : Expression pattern of Cragi-Prohormone 4 encoding gene (CHOYP-LOC658640.1.2) during development.
C : Expression pattern in adult tissues.
D : Expression pattern of Cragi-Prohormone 4 encoding gene (CGG_contig_28466, CGG_contig_14367) in the visceral ganglia during the cycle of reproduction.

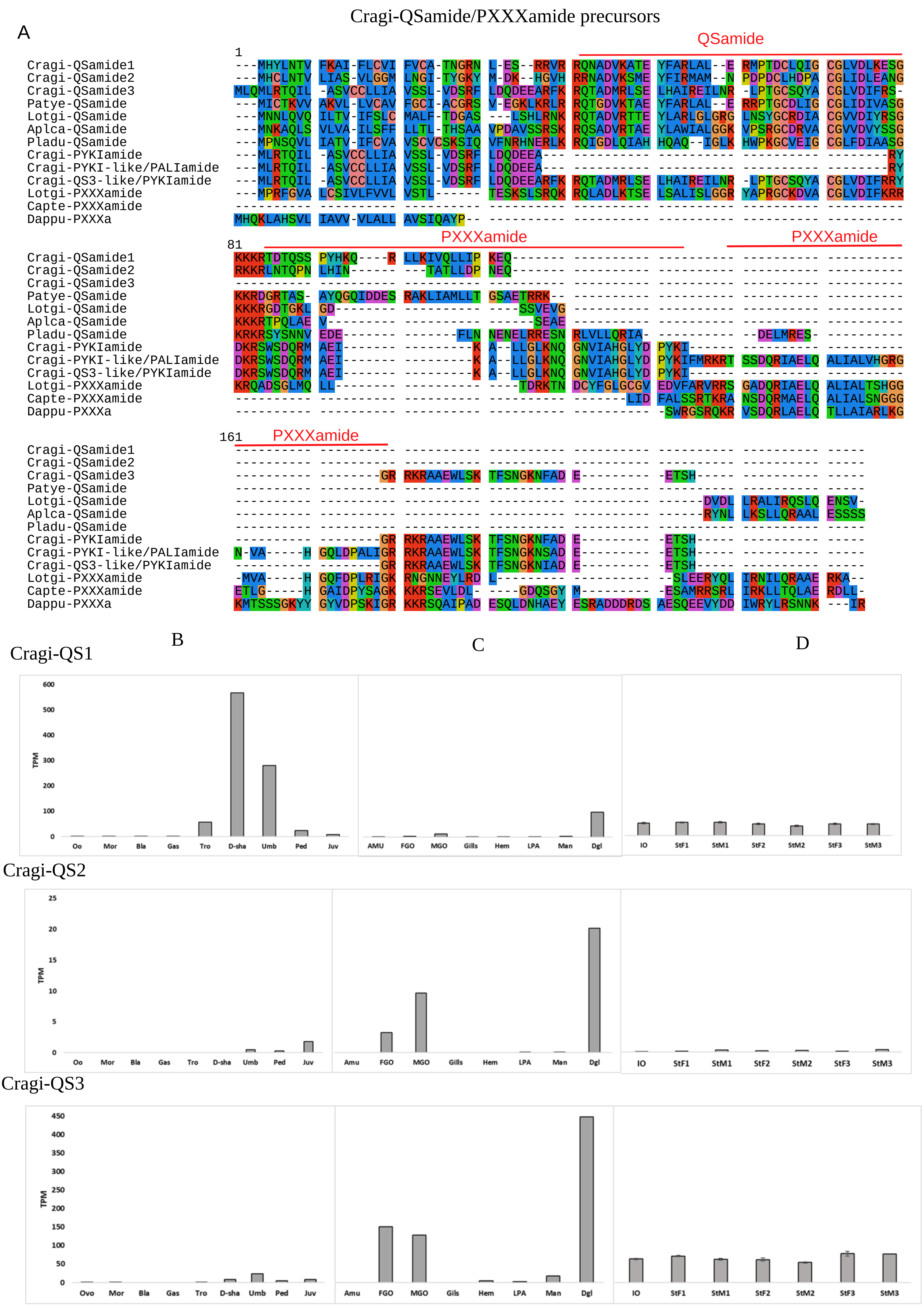
Cragi-QSamide/PXXXamide precursors
A
QSamide
1
Cragi-QSamide1 ---MHYLNTV FKAI-FLCVI FVCA-TNGRN L-ES--RRVR RQNADVKATE YFARLAL--E RMPTDCLQIG CGLVDLKESG
Cragi-QSamide2 ---MHCLNTV LIAS-VLGGM LNGI-TYGKY M-DK--HGVH RRNADVKSME YFIRMAM--N PDPDCLHDPA CGLIDLEANG
Cragi-QSamide3 MLQMLRTQIL -ASVCCLLIA VSSL-VDSRF LDQDEEARFK RQTADMRLSE LHAIREILNR -LPTGCSQYA CGLVDIFRS-
Patye-QSamide ---MICTKVV AKVL-LVCAV FGCI-ACGRS V-EGKLKRLR RQTGDVKTAE YFARLAL--E RRPTGCDLIG CGLIDIVASG
Lotgi-QSamide ---MNNLQVQ ILTV-IFSLC MALF-TDGAS ---LSHLRNK RQTADVRTTE YLARLGLGRG LNSYGCRDIA CGVVDIYRSG
Aplca-QSamide ---MNKAQLS VLVA-ILSFF LLTL-THSAA VPDAVSSRSK RQSADVRTAE YLAWIALGGK VPSRGCDRVA CGVVDVYSSG
Pladu-QSamide ---MPNSQVL IATV-IFCVA VSCVCSKSIQ VFNRHNERLK RQIGDLQIAH HQAQ--IGLK HWPKGCVEIG CGLFDIAASG
Cragi-PYKIamide ---MLRTQIL -ASVCCLLIA VSSL-VDSRF LDQDEEA--- ---------- ---------- ---------- --------RY
Cragi-PYKI-like/PALIamide ---MLRTQIL -ASVCCLLIA VSSL-VDSRF LDQDEEA--- ---------- ---------- ---------- --------RY
Cragi-QS3-like/PYKIamide ---MLRTQIL -ASVCCLLIA VSSL-VDSRF LDQDEEARFK RQTADMRLSE LHAIREILNR -LPTGCSQYA CGLVDIFRRY
Lotgi-PXXXamide ---MPRFGVA LCSIVLFVLL VSTL------ TESKSLSRQK RQLADLKTSE LSALISLGGR YAPRGCKDVA CGLVDIFKRR
Capte-PXXXamide ---------- ---------- ---------- ---------- ---------- ---------- ---------- ----------
Dappu-PXXXa MHQKLAHSVL IAVV-VLALL AVSIQAYP-- ---------- ---------- ---------- ---------- ----------
81
PXXXamide
PXXXamide
Cragi-QSamide1 KKKRTDTQSS PYHKQ----R LLKIVQLLIP KEQ------- ---------- ---------- ---------- ----------
Cragi-QSamide2 RKKRLNTQPN LHIN------ ---TATLLDP NEQ------- ---------- ---------- ---------- ----------
Cragi-QSamide3 ---------- ---------- ---------- ---------- ---------- ---------- ---------- ----------
Patye-QSamide KKRDGRTAS- AYQGQIDDES RAKLIAMLLT GSAETRRK-- ---------- ---------- ---------- ----------
Lotgi-QSamide KKKRGDTGKL GD-------- ---------- ----SSVEVG ---------- ---------- ---------- ----------
Aplca-QSamide KKKRTPQLAE V--------- ---------- ------SEAE ---------- ---------- ---------- ----------
Pladu-QSamide KRKRSYSNNV EDE------- -------FLN NENELRRESN RLVLLQRIA- ---------- --DELMRES- ----------
Cragi-PYKIamide DKRSWSDQRM AEI------- ---------K A--LLGLKNQ GNVIAHGLYD PYKI------ ---------- ----------
Cragi-PYKI-like/PALIamide DKRSWSDQRM AEI------- ---------K A--LLGLKNQ GNVIAHGLYD PYKIFMRKRT SSDQRIAELQ ALIALVHGRG
Cragi-QS3-like/PYKIamide DKRSWSDQRM AEI------- ---------K A--LLGLKNQ GNVIAHGLYD PYKI------ ---------- ----------
Lotgi-PXXXamide KRQADSGLMQ LL-------- ---------- ----TDRKTN DCYFGLGCGV EDVFARVRRS GADQRIAELQ ALIALTSHGG
Capte-PXXXamide ---------- ---------- ---------- ---------- -------LID FALSSRTKRA NSDQRMAELQ ALIALSNGGG
Dappu-PXXXa ---------- ---------- ---------- ---------- ---------- -SWRGSRQKR VSDQRLAELQ TLLAIARLKG
161
PXXXamide
Cragi-QSamide1 ---------- ---------- ---------- ---------- ---------- ---------- ---------- -----
Cragi-QSamide2 ---------- ---------- ---------- ---------- ---------- ---------- ---------- -----
Cragi-QSamide3 ---------- --------GR RKRAAEWLSK TFSNGKNFAD E--------- -ETSH----- ---------- -----
Patye-QSamide ---------- ---------- ---------- ---------- ---------- ---------- ---------- -----
Lotgi-QSamide ---------- ---------- ---------- ---------- ---------- ------DVDL LRALIRQSLQ ENSV-
Aplca-QSamide ---------- ---------- ---------- ---------- ---------- ------RYNL LKSLLQRAAL ESSSS
Pladu-QSamide ---------- ---------- ---------- ---------- ---------- ---------- ---------- -----
Cragi-PYKIamide ---------- --------GR RKRAAEWLSK TFSNGKNFAD E--------- -ETSH----- ---------- -----
Cragi-PYKI-like/PALIamide N-VA-----H GQLDPALIGR RKRAAEWLSK TFSNGKNSAD E--------- -ETSH----- ---------- -----
Cragi-QS3-like/PYKIamide ---------- --------GR RKRAAEWLSK TFSNGKNIAD E--------- -ETSH----- ---------- -----
Lotgi-PXXXamide -MVA-----H GQFDPLRIGK RNGNNEYLRD L--------- ---------- --SLEERYQL IRNILQRAAE RKA--
Capte-PXXXamide ETLG-----H GAIDPYSAGK KKRSEVLDL- ----GDQSGY M--------- -ESAMRRSRL IRKLLTQLAE RDLL-
Dappu-PXXXa KMTSSSGKYY GYVDPSKIGR KKRSQAIPAD ESQLDNHAEY ESRADDDRDS AESQEEVYDD IWRYLRSNNK ---IR
Cragi-QS1
B
C
D
TPM
600
500
400
300
200
100
0
Oo Mor Bla Gas Tro D-sha Umb Ped Juv
AMU FGO MGO Gills Hem LPA Man Dgl
IO StF1 StM1 StF2 StM2 StF3 StM3
Cragi-QS2
TPM
25
20
15
10
5
0
Oo Mor Bla Gas Tro D-sha Umb Ped Juv
Amu FGO MGO Gills Hem LPA Man Dgl
IO StF1 StM1 StF2 StM2 StF3 StM3
Cragi-QS3
TPM
450
400
350
300
250
200
150
100
50
0
Ovo Mor Bla Gas Tro D-sha Umb Ped Juv
Amu FGO MGO Gils Hem LPA Man Dgl
IO StF1 StM1 StF2 StM2 StF3 StM3

Cragi-PYKI amide

B

C

D

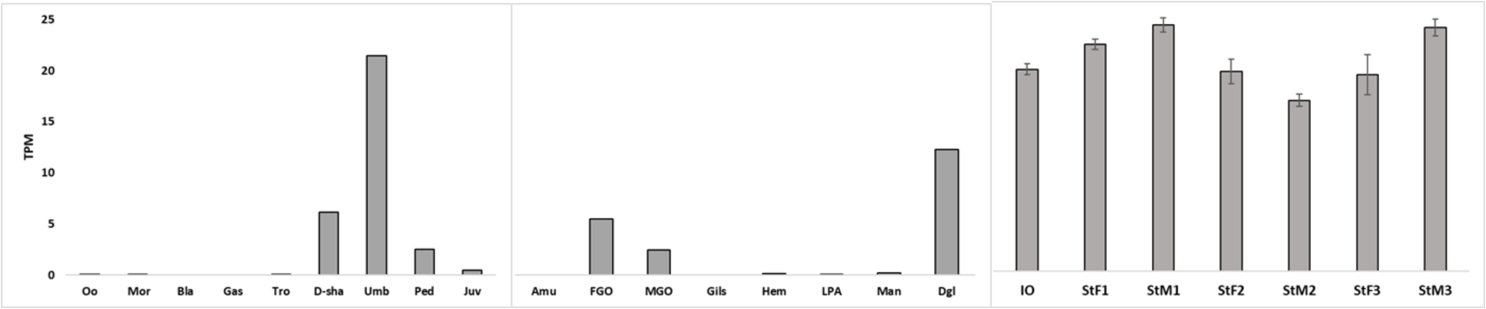

Cragi- QS3-like/PYKIamide

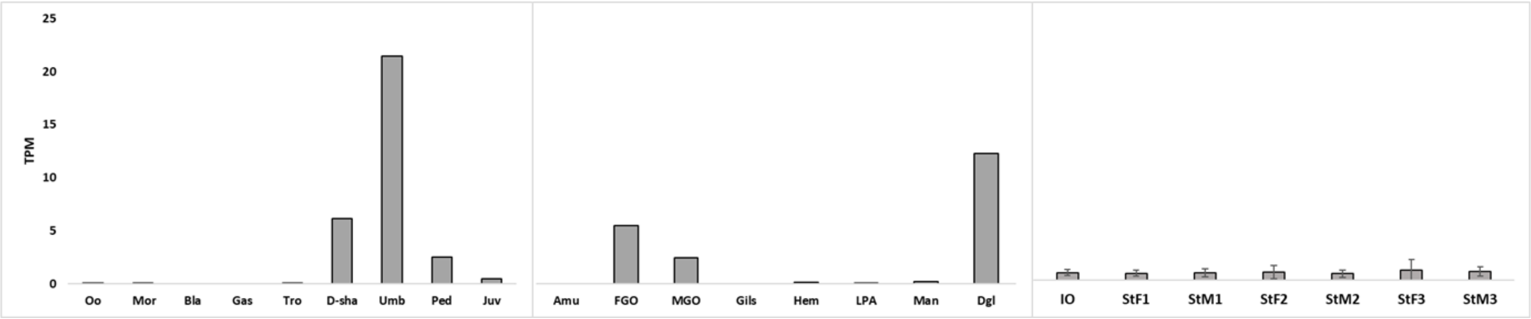

A : Alignment of *C. gigas* Cragi-QSamide1 (CGG_contig_22528, CHOYP_contig_003053, ERK20562, CGI_10005831), Cragi-QSamide2 (CGG_contig_22774, CHOYP_contig_042522) , Cragi-QS3 (CGG_contig_22898, CHOYP_contig_043914), *P. yessoensis* (XP_021353029.1), *L. gigantea* (XP_009051664.1), *A. californica* (XP_012937765.1) and *P. dumerilii* (AHB62379.1) QSamide sequences and Cragi-PYKI amide (CGG_contig_15711, CHOYP-contig-025841) Cragi-PIKI-like/PALIamide (XR-01142063.4), Cragi-QS3-like/PYKIamide (CGG_contig_23609, CHOYP_contig_004027), *L. gigantea* (ESO97822.1), *C. teleta* (ELT91968.1) and *D. pulex* (EFX77601.1) PXXXamide sequences.
B : Expression pattern of Cragi-QSamide1 (CHOYP_contig_003053), Cragi-QSamide2 (CHOYP_contig_042522), Cragi-QSamide3 (CHOYP_contig_043914), Cragi-PYKIamide (CHOYP-contig-025841) and Cragi-QS3-like/PYKIamide (CHOYP-contig-004207) encoding genes during development.
C : Expression pattern in adult tissues.
D : Expression pattern of Cragi-QSamide1(CGG_contig_22528), Cragi-QSamide2 (CGG_contig_22774), Cragi-QSamide3 (CGG_contig_22898), Cragi-PYKIamide (CGG_contig_15711) and Cragi-QS3-like/PYKIamide (CGG_contig_23609) encoding transcripts in the visceral ganglia during the cycle of reproduction.

Lophotrochozoan-specific neuropeptides

Cragi-CCWamide

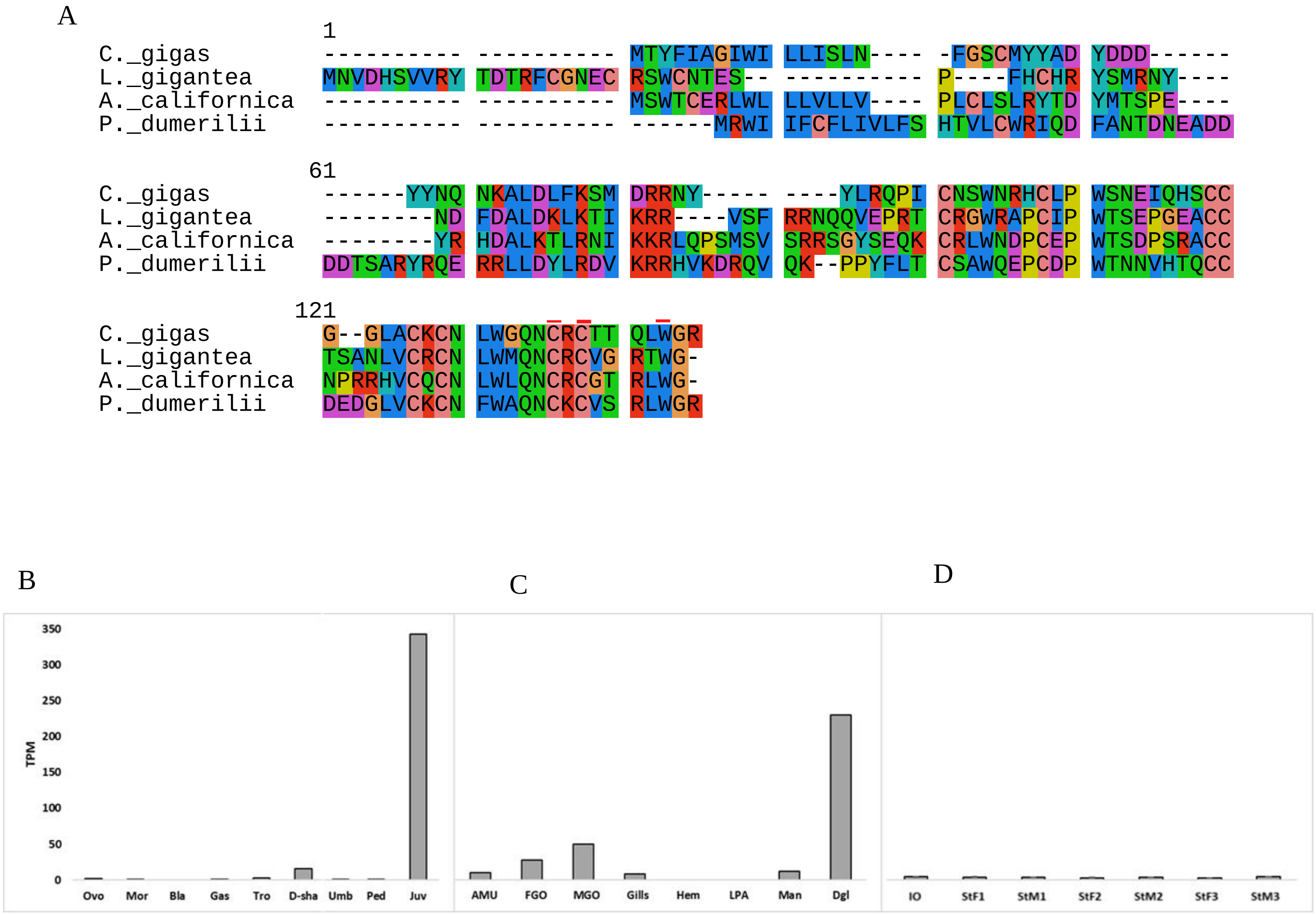

A : Alignment of *C. gigas* (CGG_contig_20696), *L. gigantea* (XP_009055999.1), *A. californica* (XP_005090647.1) and *P. dumerilii* (FP002705) CCW sequences.
B : Expression pattern of Cragi-CCWamide encoding gene (CHOYP-contig-025497) during development.
C : Expression pattern in adult tissues.
D : Expression pattern of Cragi-CCWamide (CGG-contig-20696) encoding gene in the visceral ganglia during the cycle of reproduction.

Cragi-CLCCY

A

```
                1
C._gigas        ---------- ---------- -MELKTL--- LLFSC--FLA LASGFAL--I --SGRSSSGL P----YYLKL
P._yessoensis   ---------- ---------- -MGVTTNLLL CLISCSLYIS LAHGQRIPFI RYLRTGGGGY PVMERVYLKS
P._dumerilii    ---------- --------MS SIYKMALLVL CALLL---IA LASSRPSATN Q--------- ---EEEVDQ-
C._teleta       MECRCRPGSI EGLGNTHRRN PMMTLNLLIV VLVSC---IS VADQKRHVDT R--------- ---DASWTWA

               71
C._gigas        PDRDIRETVQ FPKCSRYMET CVPDRDECCG ELTCRNILRD FCLYPLEQCR CNKLRIYQDV RR------
P._yessoensis   SGR-DREPEP EAKCGKPMDS CIPDVTECCN NMQCLNKAHS YCLYPLEMCL CISPTSHNVD ML------
P._dumerilii    --ANVARLKR RTKCGFQLEA CDPYENSCCP GMKCANLFGG YCLSGIEKCM CLPTSYGV-- --------
C._teleta       MSKRPRIAKS KHVCLSQAAE CHPFLDKCCP GMSCMNLIRG FCIYGRETCI CQSRRLHDSF YRVGRPYF
```

D

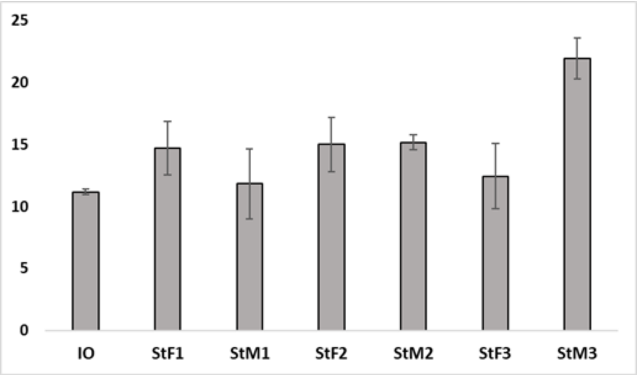

A : Alignment of *C.gigas* (CGG_contig_18747),P. *yessoensis* (XP_021368903.1), *P. dumerilii* (AHB62377.1) and *C. teleta* (ELU16055.1) CLCCY sequences.

D : Expression pattern of Cragi-CLCCY encoding gene (CGG_contig_18747) in the visceral ganglia during the cycle of reproduction.

Cragi-HFAamide

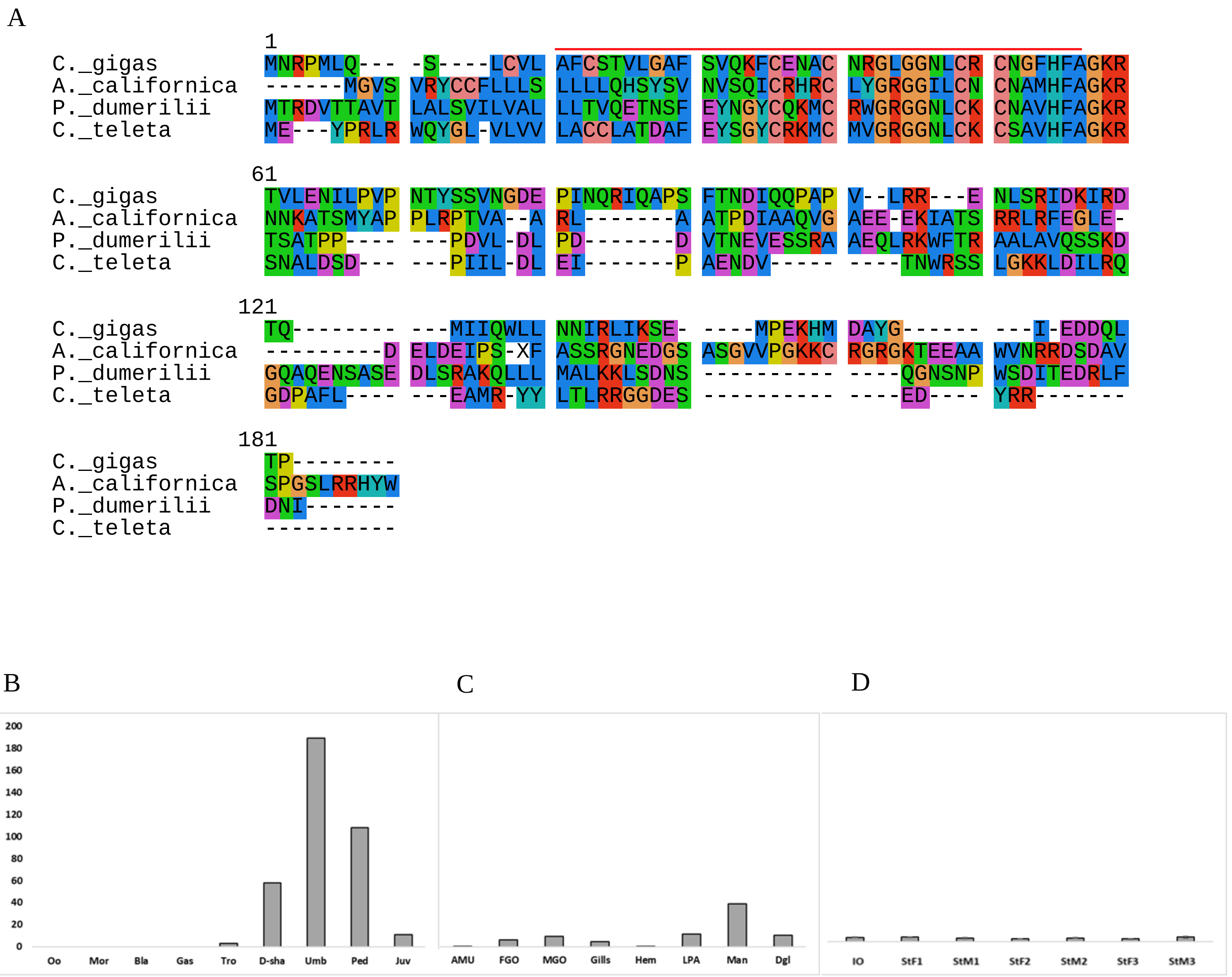

A : Alignment of *C. gigas* (CGG_contig_26304), *A. californica* (EB352987.1), *P. dumerilii* (PMID:24359412) and *C. teleta* (ELU01627.1) sequences.
B : Expression pattern of Cragi-HFAamide encoding gene (CHOYP-contig-035470) during development.
C : Expression pattern in adult tissues.
D : Expression pattern of Cragi-HFAamide encoding gene (CGG_contig_26304) in the visceral ganglia during the cycle of reproduction.

Cragi-LXRX

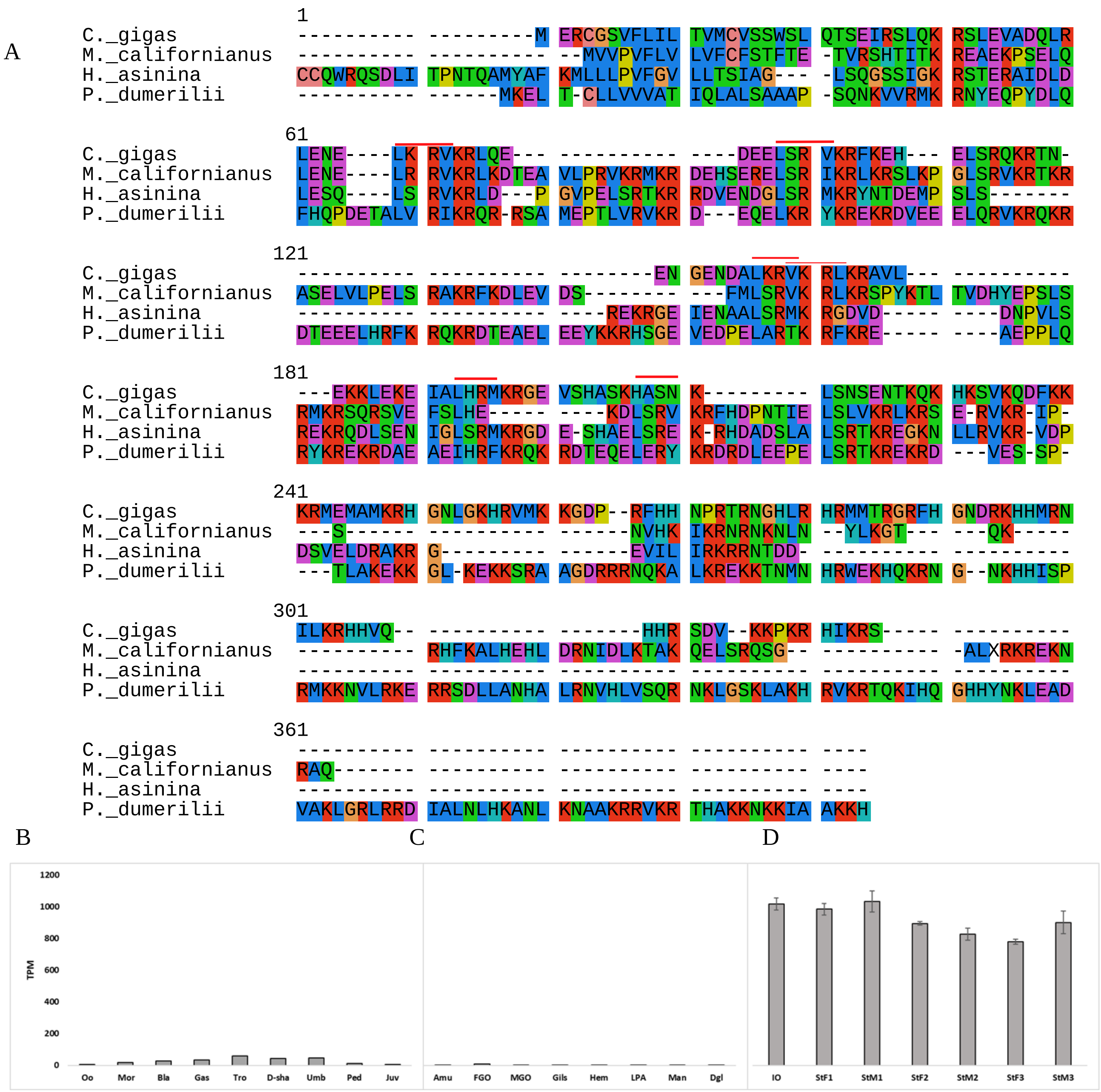

A : Alignment of *C. gigas* (CGG_contig_08893), *M. californianus* (ES394341.1), *H. asinina* (GT276618) and *P. dumerilii* (Conzelmann et al. 2013) LXRX sequences.
B : Expression pattern of Cragi-LXRX encoding gene (CHOYP-SS1G-08041.1.1) during development.
C : Expression pattern in adult tissues.
D : Expression pattern of Cragi-HFAamide encoding gene (CGG_contig_08893) in the visceral ganglia during the cycle of reproduction.

Mollusk-specific neuropeptides

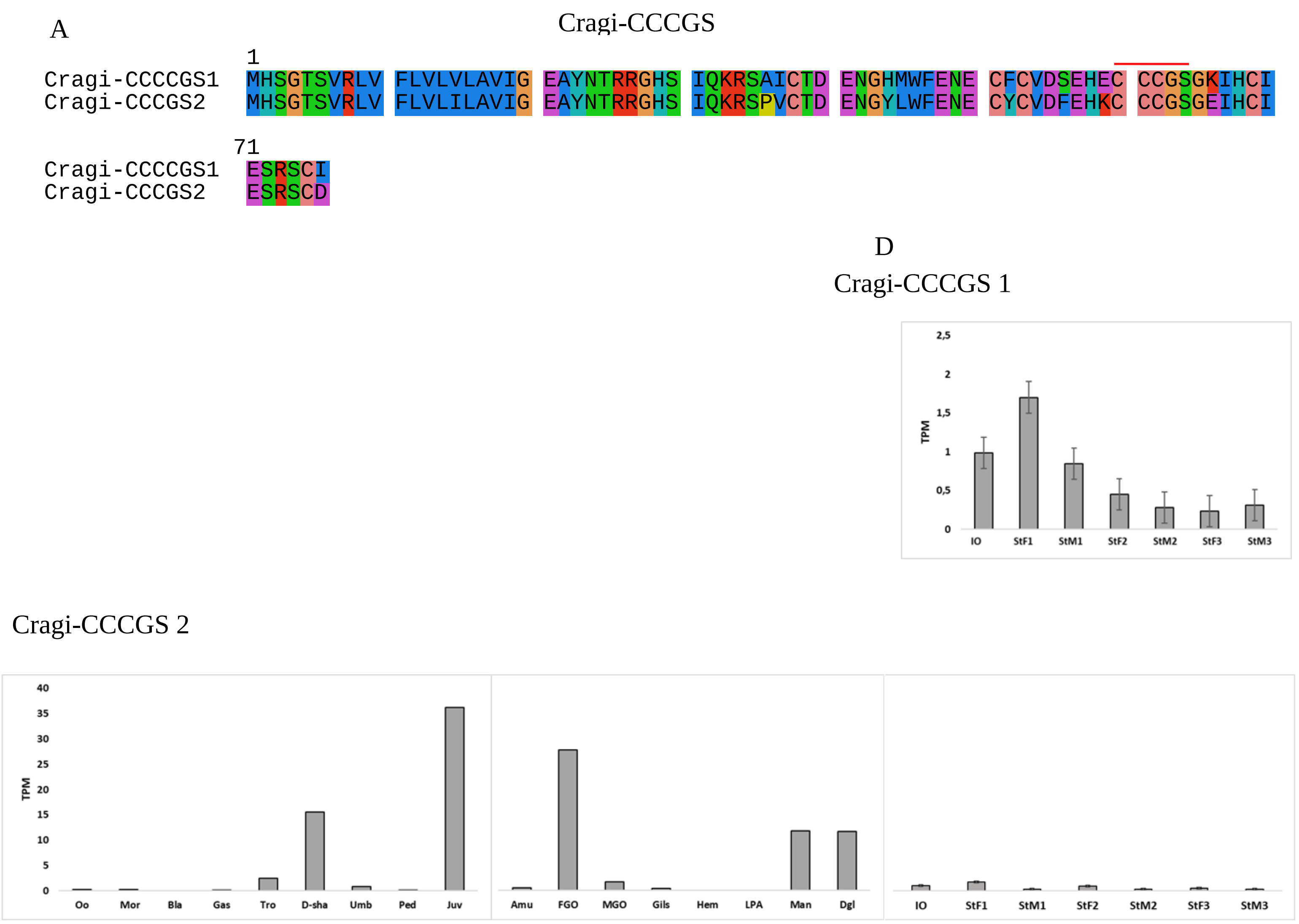

A : Alignment of *C. gigas Cragi-CCCGS1* (CGG_contig_25260, CGG_contig_16024), and *Cragi-CCCGS2* (CGG_contig_26223, CHOYP_contig_014739).
B : Expression pattern of Cragi-CCCGS2 encoding gene (CHOYP-contig-014739) during development.
C : Expression pattern in adult tissues.
D : Expression pattern of Cragi-CCCGS 1 (CGG_contig_25260) and Cragi-CCCGS 2 (CGG_contig_26223) encoding genes in the visceral ganglia during the cycle of reproduction.

Cragi-Clionin

A

```
                  1
C._gigas          --MNTCIIAV I---LALTIS TSMVKSSPIF ----PVVELS SMHPRDACLF ICSICFHS--
A._californica    -MTSSMILAV L-AI-SLSCL LTAVTSSPLG P-VQPAISLS KMEPDHACLF MCNICFPDLE
S._officinalis    MVKPTAFFTV -----VLVIA AVFMKPANLY --PTPSVPLE EMDPRDACLF QCSNCFGD--
T._diomedea       -MSPFVCFAS LATIAMFAIL LPLTSATPLE PVIDPVIALE EMEAYHACLF ICHNCFVE--

                 61
C._gigas          EENSMLKCAN NFCLSDYGFN --GLGYLWIG KEC---AHWP SLEKFVAADN VLKN------
A._californica    DTGLLLDCSN KVCGPIMAGM CAMEKLVWLG HNCRQ---YD MVQKMWAPHG AL--------
S._officinalis    QYQPLLDCAN DVCPKVKSFS -------EVG YECSTILRHP NLGRRSLFKF ----------
T._diomedea       DKEKMMKCTN QVCGTLYEDK CALDQFIWLG HHCRH---YN MIESMWSSKK RTHDVKDQFN

               121
C._gigas          --
A._californica    --
S._officinalis    --
T._diomedea       LE
```

D

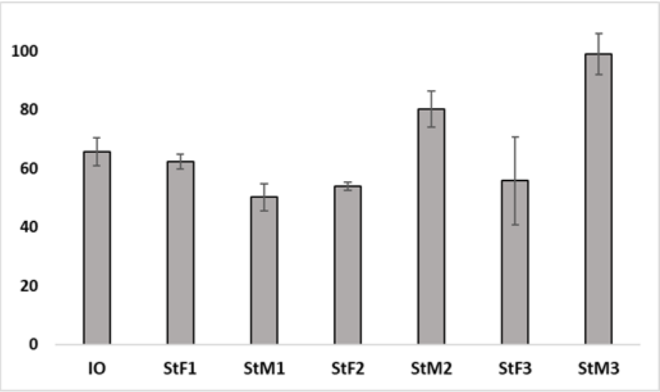

A : Alignment of *C. gigas* (CGG_contig_22908, XP_011412322.1), *A. californica* (XP_005099829.1), *S. officinalis* (Zatylny-Gaudin et al., 2016) and T. *diomedea* (I1SKI5) Clionin sequences.
D: Expression pattern of Cragi-Clionin encoding gene (CGG_contig_22908) in the visceral ganglia during the cycle of reproduction.

Cragi-FYFY

A

```
                 1
C._gigas1        MCAMKVLGVC FIVIFALQQR LFVRAETIVR YQQEQLETAV CE-ENAVCTE LMALP----R ----DNYFSL
C._gigas2        MYSLV-LTTT LIMVLTIAD- --GKDVFI-- ---TRSNSPI CP-PLAVCGN VFEFDRMMDD GSVEPERRHI
P._yessoensis    MCS----TTC WIAVLATF-V ACTRGTSITR YQH--FPLPM CS-EDTVCSE ISELPSIKEE ----NNYFSW
L._gigantea      ---------- -MATLTLSE- ----PIFLHR ---PNEPRPG CPSANTICST IYQDS----P ENIGDEYLVN

                71
C._gigas1        KSCSCPNGYT CPERPG-TST LPFGRGRWYG LCRPVSDIPE CSPGEVARKQ YNGVRDIGYL KYTQIHCLCP
C._gigas2        TNCFCNNSRV CPFNRE-N-M IYQSRTQQEV LCEPVRDLPR CRPGMVARRM YVDSMDFNDK SYYAIRCICP
P._yessoensis    MMCQCPGESV CPKSPG-KQT IEVTGSKWYG MCRPKSNIRS CSPGEVGEEY YMGVREIPFG TFTRVHCLCP
L._gigantea      SICSCQNGQS CPVDIDHNNT LQVDDGVWYG LCQPTKDFSE CKVNDVATRS FENSDKFQGH GYSELNCICT

               141
C._gigas1        GDGQIGMVPS TVWTKTR-RD DPRADSVFEF ECADQTH--- ---------- ------SGSP S-----RKRS
C._gigas2        LNLVPSSRPR VKATVYRNLQ FEGFDRIHNY KCNEEDV--- ---------- ------EEYK ----------
P._yessoensis    LLEYEEGQP- ------E-MD ASNPKSIYQL ICSGTGR--- ---------- ------LQAA MKRGRGGSRY
L._gigantea      SENKVVLENK IRSEDS--EY GPGVDK---- ICGEEVEKRG IDQGMSSFYK RSQDNGLSSF YKRGSGLSAF

               211
C._gigas1        RGRGGF-RKF Y--------F YK-------- ---------- ---------- ---------- ----------
C._gigas2        ---------- ---------- --K------- ---------- ---------- ---------- ----------
P._yessoensis    GGGKNG-RRF Y--------F YRK------- ---------- ---------- ---------- ----------
L._gigantea      YKRGNGLSSF YKRGHDLSAF YKRSRGLNSF YKRRSPFQHV SSFYKRDLGH VSSFYKRAPE NSLSSFYKRS

               281
C._gigas1        ---------- ---------- ---------- ---------- ---------- ---------- ----------
C._gigas2        ---------- ---------- ---------- ---------- ---------- ---------- ----------
P._yessoensis    ---------- ---------- ---------- ---------- ---------- ---------- ----------
L._gigantea      HDIGMNSFYK RSRDNGMSSF YKRSPNDLSN FYKRVPVGLR NFYKRSVGND MLGFYKRTPS SYDLSSFYKR

               351
C._gigas1        ---------- ---------- ---------- ---------- ---------- ---------- ----------
C._gigas2        ---------- ---------- ---------- ---------- ---------- ---------- ----------
P._yessoensis    ---------- ---------- ---------- ---------- ---------- ---------- ----------
L._gigantea      ASGNNALSSF YKRARENDQL SGFYKRAAGN DDLSSFYKRS SGNDDLASFY KRAAGNDDLS SFYKRAAGND

               421
C._gigas1        ---------- ---------- ---------- ---------- ---------- ---------- ----------
C._gigas2        ---------- ---------- ---------- ---------- ---------- ---------- ----------
P._yessoensis    ---------- ---------- ---------- ---------- ---------- ---------- ----------
L._gigantea      NLSSFYKRAA GNDGLSNFYK RAAGNDGLSS FYKRAAGNDN LSNFYKRAAG NDDLSSFYKR AAGNNDLSSF

               491
C._gigas1        ---------- ---------- ---------- ---------- ---------- ---------- ----------
C._gigas2        ---------- ---------- ---------- ---------- ---------- ---------- ----------
P._yessoensis    ---------- ---------- ---------- ---------- ---------- ---------- ----------
L._gigantea      YKRAAGNDDL SGFYKRAAGN DNLYNFYKRA PVASQLSNFY KRGLSSNDLV AFYKRALGNG VSSFYKRANS

               561
C._gigas1        ---------- ---------- ---------- ---------- ---------- ---------- ----------
C._gigas2        ---------- ---------- ---------- ---------- ---------- ---------- ----------
P._yessoensis    ---------- ---------- ---------- ---------- ---------- ---------- ----------
L._gigantea      DLSSFYKRAV DDVSNFYKRS GEDDQTNDNS LSSFYKRSLN GGQNSFYKRS PLDHDVSHFY KRGLDDVSSF

               631
C._gigas1        ---------- ---------- ---------- ---------- ---------- ---------- ---
C._gigas2        ---------- ---------- ---------- ---------- ---------- ---------- ---
P._yessoensis    ---------- ---------- ---------- ---------- ---------- ---------- ---
L._gigantea      YKRNANRLQS FYKRARDNTS NFYKRSPEQQ SSFYKRAPKD NVSSFYKRDS SDNKDVAFYK RSV
```

B

Cragi-FYFY 1

C

D

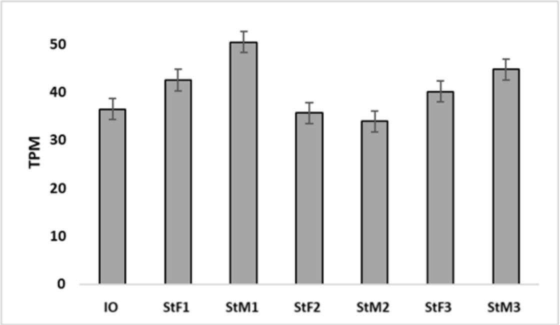

Cragi-FYFY 2

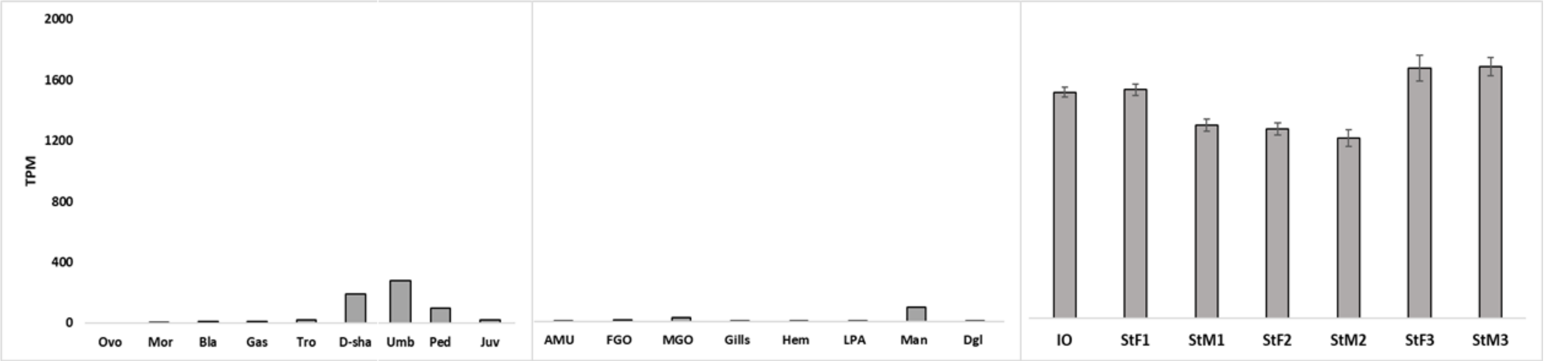

A : Alignment of *C. gigas*1 (CGG_contig_04983), *C. gigas*2 (CGG_contig_26082), *P. yessoensis* (KP79_PYT20341) and *L. gigantea* (XP_009065476.1) FYFY sequences.
B : Expression pattern of Cragi-FYFY2 encoding genes (CHOYP-contog-025391) during development.
C : Expression pattern in adult tissues.
D : Expression pattern of Cragi-FYFY1(CGG_contig_04983) and Cragi-FYFY2 (CGG_contig_26082) encoding genes in the visceral ganglia during the cycle of reproduction.

Cragi-GNamide

A

```
                 1
C._gigas         ---MYTHRLT VLLLLICLSL GHAQWAQTFG WGGAGNGKRS AWSTSSNSEK YDCSQNNENI LNVISSLVQL
P._yessoensis    MNSLPYVTVS MLLLLSHCDL GHAQWAQTFG WGGAGNGKRS GITDPTT--- --CN-MDLQL VKLMSTLIQH

                71
C._gigas         EVQRLNYCEN KRRMLTQ---
P._yessoensis    ELKRLKTCSE QKIPQDLLDI
```

D

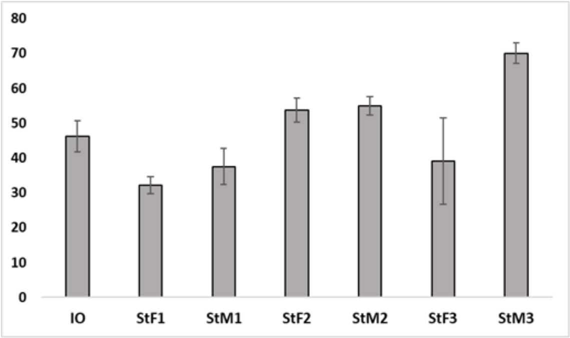

A : Alignment of *C. gigas* (CGG_contig_01838) and *P. yessoensis* (MH045254) sequences.
D : Expression pattern of Cragi-GNamide encoding gene (CGG_contig_01838) in the visceral ganglia dduring the cycle of reproduction.

Cragi-GRWRN

A

```
                 1
C._gigas         MVSKECMSLT SLL-VVVLLA SSVVCKDEHY HDNLSDRDVN KRISIFNWRL LYKLYKLKPG LFFR--SRTG
C._virginica     MSPMETLSLR PLIVVVMLIA STAVCQDEKI PEDTTNQGET -KRVFFNWRI LSKIHRERPG FFSISRSRTG
P._yessoensis    MNVRHR---- ---------- ---------- ------NGIT NVLMSMCFLI FFYDVMALPG KQV---SDGG

                71
C._gigas         RAS----SIT QENDGLQKSP ESI-NTPKQG ESPYVPL--P PYLFTSSHLL ANDENEAPKV KRNIGRWRNT
C._virginica     RSS----DFT LENDVNALPL KLLLQSVDTE TLPDFQI--S PDLFPNDNT- -PTDNSLTKE KKNLGRWRNI
P._yessoensis    RKHITSGDFD SL-NTEEIPS ELFLDINDDD GIDDLEHQYE VQQFDDPRY- -VTVEEPGVG KRFLGRWRNL

               141
C._gigas         NWIYSIRPE- ------RRRF SSPYRKK--- ---------- ---------- ---------- ---
C._virginica     NWIYSIRPD- ------GPKF NYLFKTN--- ---------- ---------- ---------- ---
P._yessoensis    NWKLRQRELN KDDVPVGKRF FGRWMNRNYI LSKLEAERKN QVGKRFLGRW RNRNYLLEKM KDE
```

B C D

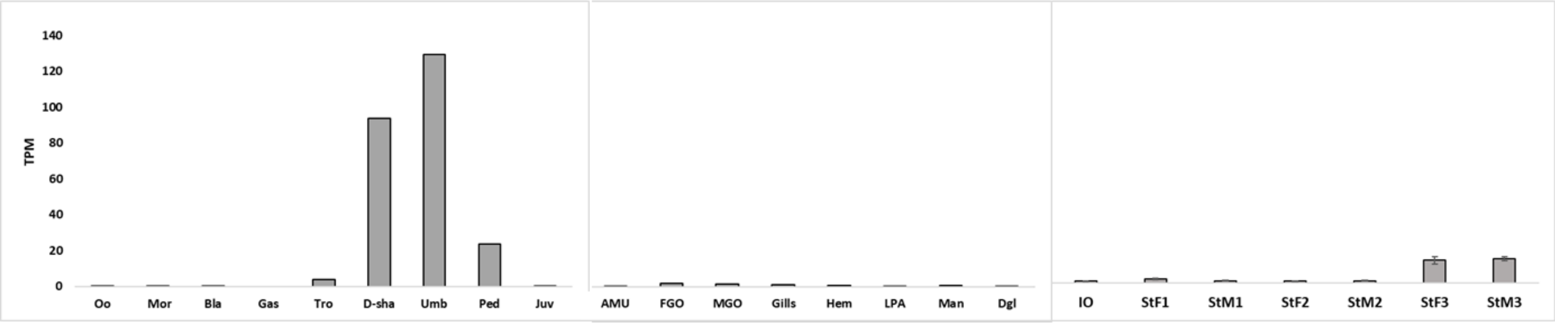

A : Alignment of *C. gigas* (CGG_contig_15181), *C. virginica* (XP_022337980) and *P. yessoensis* (XP_021372506) GRWRN sequences.
B : Expression pattern of Cragi-GRWRN encoding gene (CHOYP-contig-008923) during development.
C : Expression pattern in adult tissues.
D : Expression pattern of Cragi-GRWRN encoding gene (CGG_contig_15181) in the visceral ganglia during the cycle of reproduction.

Cragi-GSWN

A

```
                 1
C._gigas         MPRQLYTALF L---VLVALL LSSTPEVSAK SSCSYACMKT LFSCKRRS-- -----EGDCC
C._virginica     -MSRLYTFLA M---TLIAS- IFTTTEVGAK SSCSYDCMKS LFACKRRS-- -----EGDCC
L._gigantea      --MKRFTLLA L---LLVAIL YISQS--ESK CQGVTKCMVN LFTCKREQIS VN--IAADCC
A._californica   --MELTSPLL LVISSFLAVY VINFTDAGPL DPCVFQCMRG VLKCKREQSV VGLSQKVDCC
P._yessoensis    --MKAFCIL- -----LALAV FMASMTTTSG RSCYFMCLRD LILCKRTT-- -----GTGCC

                 61
C._gigas         NKFTECFHTC DLAA---PPC ----AGKRGS WNKRYQVSQG YED-RE-LYP YY--------
C._virginica     NKFTDCFLAC GLSA---PPC ----AGKRGS WNKRYEVPGR YED-GE-LYP YY--------
L._gigantea      KEYFECFNEC KPGSR--PPC KNTAPGKRGS WNKRYSSRYG SDILGD-IIS GY--------
A._californica   QKYKMCYLVC RPDAESVPPC -GGAKGKRGS WNKRTQAANT CNNNGHQHVP DFDDVVDLGQ
P._yessoensis    DKFLG---SC APGCG--VKN AKCKGTKRGS WNKRADYEE- -NPLEE-YYP MI--------

                 121
C._gigas         ---
C._virginica     ---
L._gigantea      ---
A._californica   RFY
P._yessoensis    ---
```

D

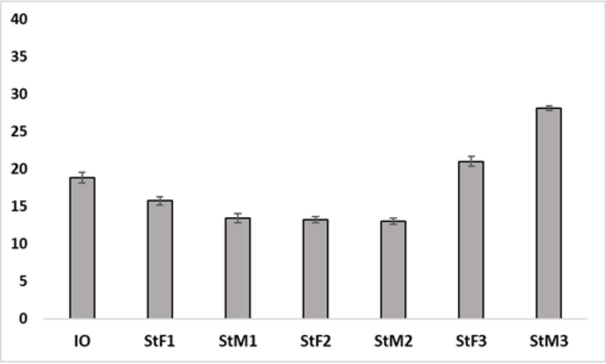

A : Alignment of *C. gigas* (CGG_contig_26157), *C. virginica* (XR_002636430.1), *L. gigantea* (XM_009047650.1), *A. californica* (XM_013087810.1) and *P. yessoensis* (XR_002464777.1) GSWN sequences.
D : Expression pattern of Cragi-GSWN encoding gene (CGG_contig_26157) in the visceral ganglia during the cycle of reproduction.

Cragi-GWE

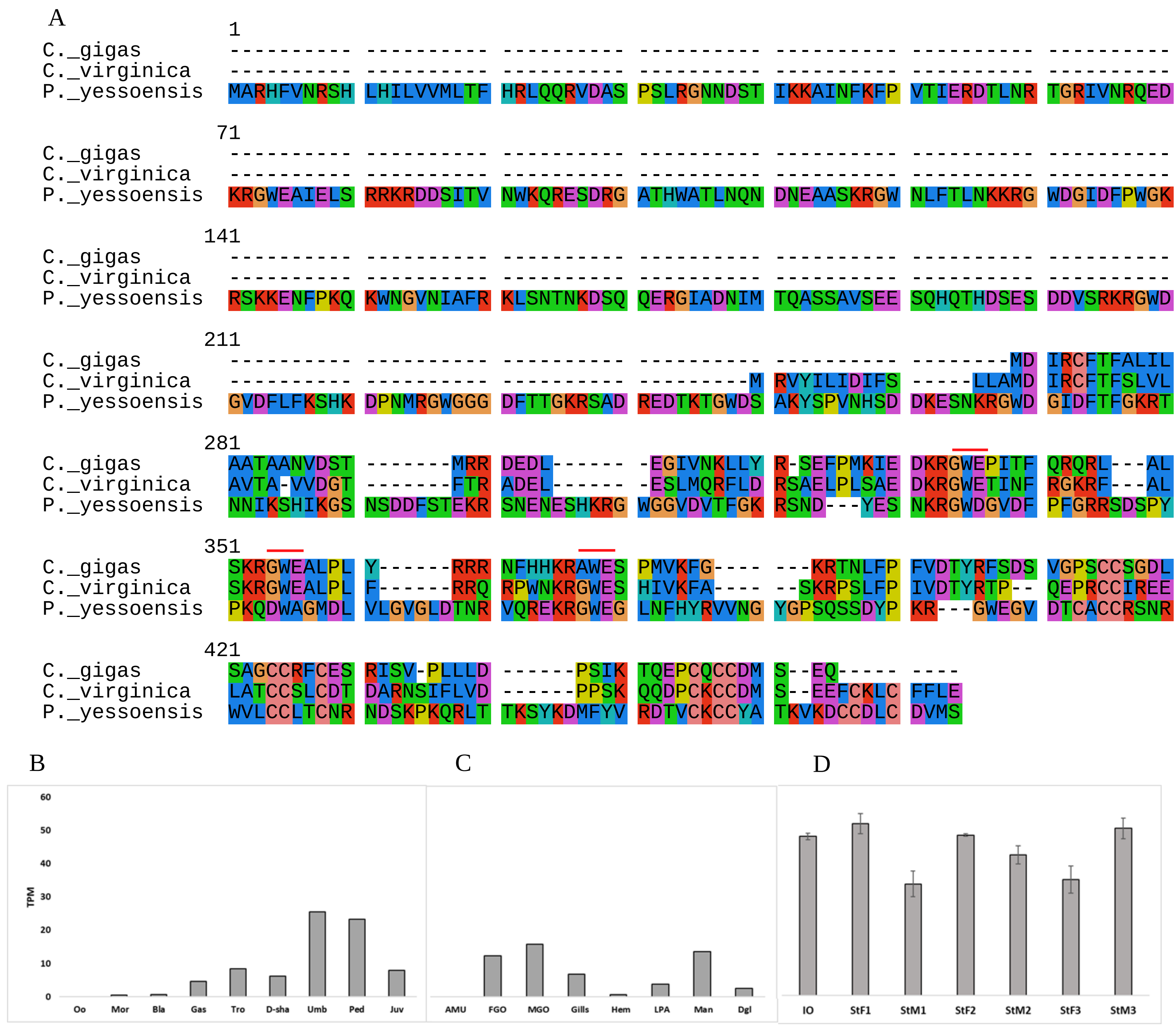

A : Alignment of *C. gigas* (CGG_contig_24907), *C. virginica* (XP_022318468.1) and *P. yessoensis* (MH045253) GWE sequences.
B : Expression pattern of Cragi-GWE encoding gene (CHOYP-contig-049345) during development.
C : Expression pattern in adult tissues.

Cragi-IWMPxxGYxx

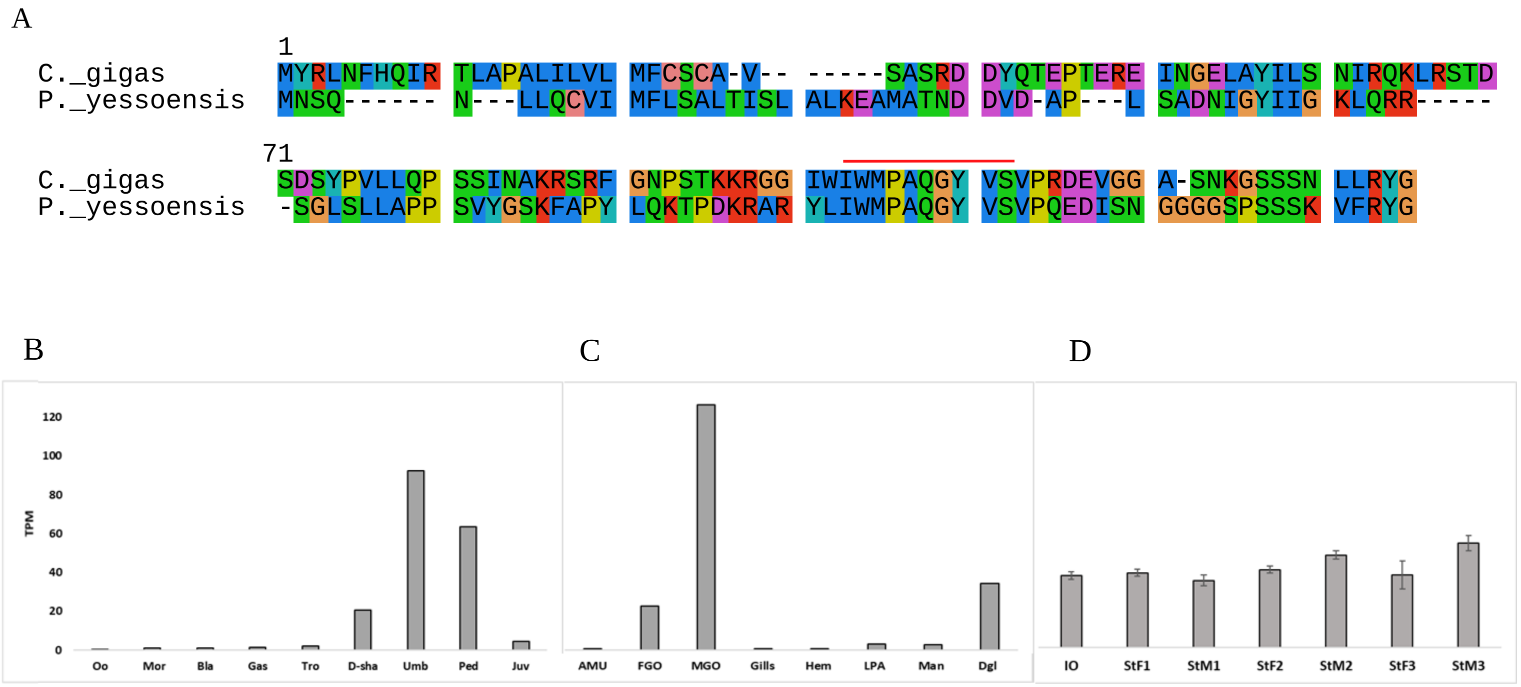

A : Alignment of *C. gigas* (CGG_contig_09620) and *P. yessoensis* (MH045262) IWMPxxGYxx sequences.
B : Expression pattern of Cragi-IWMPxxGYxx encoding gene (CHOYP-contig-025394) during development.
C : Expression pattern in adult tissues.
D : Expression pattern of Cragi-IWMPxxGYxx encoding gene (CGG_contig_09620)in the visceral ganglia during the cycle of reproduction.

Cragi-LXRYamide

A

```
                 1
C._gigas         MMNWGLTVML LAALTCVF-- --KCEDGSDA EGWI------ -----VESTD YNSQ-VSLLK KLIESEMNKE
P._yessoensis    -MH---GQIM LPVILCLISM LSSFTDATDA SGEVPISSGN LLREILESRD FTDVEKEILK YIIFSDVK--

                71
C._gigas         RRQKQFEEEM ARLYMDSSPS ELRPVPRKKS YLWFRSKSSA NPVQTVQTRV SIGQSLHPPP EETNKPKGLF
P._yessoensis    ----DFVERK -EASAESRED SDSGIERKRS HLWAYRQNNI -PI---QTRV AFGRQLMRNS NGHGSNSNLL

               141
C._gigas         RYGKK
P._yessoensis    RYGK-
```

D

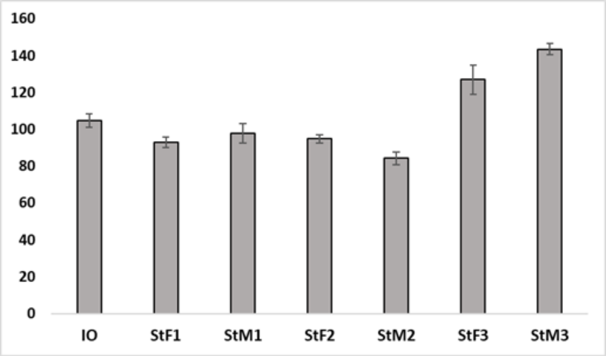

A : Alignment of *C. gigas* (CGG_contig_20802) and *P.yessoensis* (AXN93520.1) LXRYamide sequences.
D : Expression pattern of Cragi-LXRYamide encoding gene (CGG_contig_20802) in the visceral ganglia during the cycle of reproduction.

Cragi-RTLFamide

A

```
               1
C._gigas       MKTGVLFYVT IVCGISASVV CARNSQFKHL PVFPGFLNRP LSTSPHFDLQ NLYRTVDKRF NMDNRAFGED
L._gigantea    ---------- ---------- MMMIKHWNRL -IFCGLVVDH LHKSAFY-LQ S------KKY NIDQETYGED

              71
C._gigas       DRFNHFMDWR TLFGKRS--- ---------- ----FGGPDK IGFHNMESPF KSYYQNWRER FFPKKKGANK
L._gigantea    PYFSGVLATH FVYGLQGNNT RYIRANAGCK HFDVYAGPEN VPVSRFSFDA KVSERDWRTT FLPQFRACVE

             141
C._gigas       --
L._gigantea    AG
```

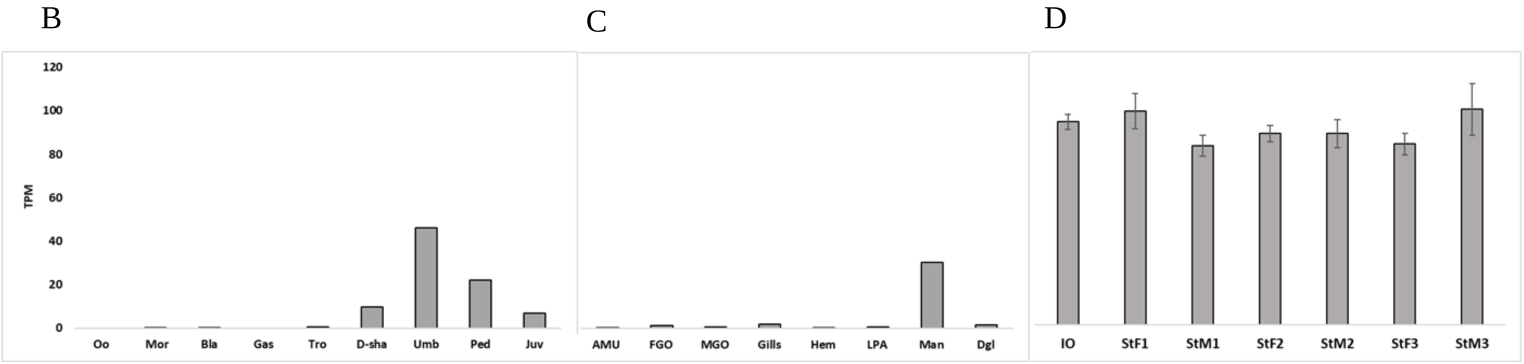

A : Alignment of *C. gigas* (CGG_contig_04103) and *L. gigantea* (XM_009063916.1) RTLFamide sequences.
B : Expression pattern of Cragi-RTLFamide encoding gene (CHOYP_contig_039290) during development.
C : Expression pattern in adult tissues.
D : Expression pattern of Cragi-RTLFamide encoding gene (CGG_contig_04103) in the visceral ganglia during the cycle of reproduction.

Cragi-SLRFamide

A

```
                 1
C._gigas         MNISSAAMLR VFVVALWAIA ILCQ----NT NGLTALENIL A-----EETK DDYLRDVDDD SVLDQRTREE
C._virginica     MNILSNSVLR LFVVALWAIA ILCR----NT NGLTALENIL A-----EETK DDYLRDVDDD SALDQRTREE
P._yessoensis    MEV------T TYKVAIAVLM FVCLMISDSQ CGIP-LNSFL SASNGKLERD ADDVDNFDEE TANIQGVRGQ

                71
C._gigas         DLKKLILKKL RFRELPNDSS SEVLNSLVKR VPDSFRYGDS LVDKVAALLR I-KM-RPTKS PQVRMPSLRF
C._virginica     DLKKLILKKL RFRELPNDSS SEVLSNLVKR VPDSYRFGES LVDKVAALLR F-KM-RPTKS PQVRMPSLRF
P._yessoensis    DLRKLILKKL RFRDLDDQSP PSLLESIQFP VK---RDQDN LVDKVAALLS GLKVRQISDS PSVRMPSLRF

               141
C._gigas         G
C._virginica     G
P._yessoensis    G
```

B C D

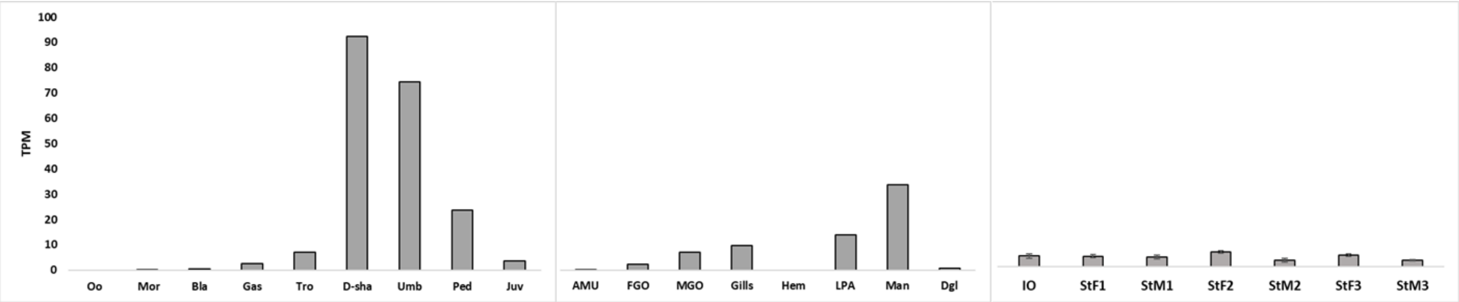

A : Alignment of *C. gigas* (CGG_contig_15407), *C. virginica* (XP_022327616.1) and *P. yessoensis* (XP_021354453.1) SLRFamide sequences.
B : Expression pattern of Cragi-SLRFamide encoding gene (CHOYP-contig-005956) during development.
C : Expression pattern in adult tissues.
D : Expression pattern of Cragi-SLRFamide encoding gene (CGG_contig_15407) in the visceral ganglia during the cycle of reproduction.

Cragi-WGAGamide

A

```
                1
C._gigas       -MWNFATVKC LV------FI LTLPGFLA-- --RIWDY--- --E-YMDRRN ERPPFRGLPD ILRELYHKHS
C._virginica   MMWSLGTFMC LF------FV ITLPGCLA-- --DIWNH--- --G-YRNKNI DRPSFLGLPD LLRGAWTRQS
P._yessoensis  --MQPNIYVC SYVAVVVVIL STIAHCYPSK QIDLFTDSLP DDSYFFEDRS DRRASLAAPV LS-----LSS

               71
C._gigas       QNL-PPNRHI ATVKRGDRYS INDLIAYLKG MVGSDEIYHT GRSTYVRWGA GG--
C._virginica   RDP-SPEHRS PTVKRGDRYS INDLIAYLKG MVGSDEIYHT GRSTYVRWGA GG--
P._yessoensis  RTYHPLQQLI KRGYQNKRYS LNDLIARLRA MVGSDEIRHS GRSSYLRFGA GGKR
```

D

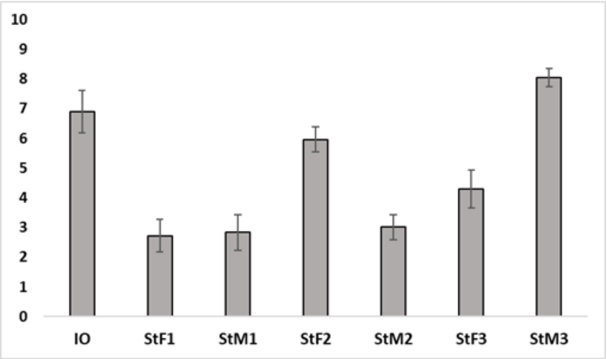

A : Alignment of *C. gigas* (CGG_contig_15208), *C. virginica* (XP_022335259.1) and *P. yessoensis* (A0A210QE07) WGAGamide sequences.
D : Expression pattern of Cragi-WGAGamide encoding gene (CGG_contig_15208) in the visceral ganglia during the cycle of reproduction.
